# CLASHub is an integrated database and analytical platform for microRNA-target interactions

**DOI:** 10.1038/s41467-026-72902-x

**Published:** 2026-05-08

**Authors:** Lu Li, Peike Sheng, Nicholas M. Hiers, Tianqi Li, Acadia L. Grimme, Yuzhi Wang, Conner M. Traugot, Olivia M. D’Agati, Mingyi Xie

**Affiliations:** 1 Department of Biochemistry and Molecular Biology, University of Florida, Gainesville, FL USA; 2 UF Health Cancer Institute, University of Florida, Gainesville, FL USA; 3 Laboratory of Cellular and Developmental Biology, NIDDK Intramural Research Program, Bethesda, MD USA; 4 UF Genetics Institute, University of Florida, Gainesville, FL USA; 5 Present Address: Department of Molecular, Cellular and Developmental Biology, Yale University, New Haven, CT USA

**Keywords:** miRNAs, Computational platforms and environments

## Abstract

MicroRNAs (miRNAs) are short RNAs that regulate gene expression, critical for development and disease. Residing in Argonaute (AGO) proteins, miRNAs target messenger RNAs via complementary base-pairing. Current miRNA-target databases rely on indirect data from AGO crosslinking immunoprecipitation (AGO-CLIP). In contrast, CLASH (Crosslinking, Ligation, and Sequencing of Hybrids) employs proximity ligation within AGO complexes, providing direct miRNA-target interaction evidence. Existing CLASH datasets remain limited to a few human and mouse samples. Here, we present CLASHub, which integrates CLASH-defined interactions with gene and miRNA expression data from human, mouse, *Drosophila*, and *C. elegans*, spanning 25 cell types and tissues, including 91 new CLASH datasets generated from 17 cell types/tissues. The datasets also include samples with knockout of *ZSWIM8*, an essential component in target-directed miRNA degradation (TDMD), providing insights into miRNA turnover mechanisms. CLASHub features a user-friendly Analyzer interface for CLASH, RNA-seq, miRNA-seq, and cumulative fraction curve analyses. Leveraging these tools, we uncover a TDMD trigger in the *ATP6V1G1* 3′ UTR for miR-335-3p degradation, as well as multiple targets of miR-18a-5p. Thus, CLASHub is an online platform that enables cell/tissue-specific exploration of miRNA-target interactions, supporting miRNA and broader RNA biology research. The platform is publicly accessible at https://clashub.rc.ufl.edu/.

## Abstract (web summary)

MicroRNAs control gene expression, but their direct RNA targets and turnover mechanisms remain difficult to study. Here, the authors present CLASHub, an integrated database and analysis platform that maps microRNA–target interactions and reveals new insights into microRNA degradation and targeting.

## Introduction

MicroRNAs (miRNAs) are small non-coding RNA molecules, 20–24 nucleotides in length, that play pivotal roles in post-transcriptional gene regulation in eukaryotic cells^1^. They function by guiding the miRNA effector protein Argonaute (AGO) to complementary sequences within target RNAs—most commonly within the 3ʹ untranslated regions (3′ UTRs) of messenger RNAs (mRNAs)—to induce mRNA degradation or inhibit translation, thereby fine-tuning gene expression^2^. While miRNA-target sites can also be found in coding sequences (CDSs) and 5′ UTRs, these are generally less frequent or less effective than those in the 3′ UTR^3,4^. Through this regulatory mechanism, miRNAs control a multitude of cellular processes such as development, differentiation, proliferation, and apoptosis^5–7^. Consequently, aberrations in miRNA expression or function can disrupt these critical processes, contributing to the pathogenesis of various diseases, including cancer, neurodegenerative disorders, and cardiovascular conditions^8^.

Understanding miRNA-target interactions is fundamental to unraveling the complexities of gene regulatory networks. High-throughput sequencing approaches, particularly crosslinking immunoprecipitation (CLIP)-based techniques such as HiTS-CLIP and PAR-CLIP, have provided valuable insights into miRNA-binding sites genome-wide^9–12^. These methods rely on ultraviolet (UV) light-induced crosslinking of RNA–protein complexes, immunoprecipitation of AGO-bound RNAs, followed by sequencing to identify miRNA-interacting RNA fragments. However, CLIP-based methods identify AGO binding sites on target RNAs but do not directly reveal which specific miRNAs are engaged at those sites. To overcome this limitation, the CLASH (crosslinking, ligation, and sequencing of hybrids) approach was developed, which resembles AGO-CLIP protocols but with an additional miRNA-target proximity ligation step to physically join miRNAs directly to their interacting target RNAs^13,14^. These resultant miRNA-target chimeras or hybrids are then sequenced to generate direct evidence of specific miRNA-target interactions and their exact binding sites. Thus, CLASH significantly enhances the accuracy and resolution of miRNA regulatory network analyses.

Despite advances in experimental techniques, challenges remain in the comprehensive identification and analysis of miRNA-target interactions across diverse species and cellular contexts. Existing databases such as miRTarBase^15^, TargetScan^16^, and miRDB^17^ compile predicted and experimentally validated miRNA targets but often lack cell-type-specific information and direct evidence of interactions. Moreover, few resources integrate data from multiple organisms or provide tools for analyzing how changes in miRNA expression influence target gene expression globally^18,19^. To address these challenges, CLASHub 1.0 (hereafter, CLASHub) was developed as a user-friendly platform that houses a rich database of CLASH-derived miRNA-target interactions. CLASHub aggregates and standardizes CLASH data from diverse studies, incorporating both newly generated and previously published datasets. The database includes CLASH data from four species—*Homo sapiens* (human), *Mus musculus* (mouse), *Drosophila melanogaster* (fruit fly), and *Caenorhabditis elegans* (nematode)—spanning a variety of cell types and tissues. In addition to CLASH-derived miRNA-target interaction data, CLASHub provides gene and miRNA expression profiles obtained from publicly available datasets, allowing for an informed understanding of miRNA-target interactions with RNA abundance.

Beyond serving as a comprehensive database, CLASHub provides powerful analytical tools that extend its functionality. For CLASH analysis, users can upload raw sequencing files to systematically identify miRNA-target RNA hybrids. The web portal returns a detailed table specifying miRNA-target pairs, genomic coordinates, base-pairing patterns, target conservation scores, RNA types (e.g., mRNA, ncRNA), transcript location (e.g., 3ʹ UTR, CDS), and hybrid abundance. The integrated RNA-sequencing (-seq) and miRNA-seq analysis modules allow users to quantify global gene expression and miRNA changes, facilitating functional validation of miRNA targets identified by CLASH. Furthermore, direct evaluation of the global impact of specific miRNAs on target gene expression can be achieved by uploading differential gene expression results. These results can then be used to produce cumulative fraction curves that detail changes in miRNA-target repression, utilizing target predictions from both CLASH-derived and TargetScan datasets. Collectively, these combined analyses streamline discovery and validation, significantly enhancing the efficiency and depth of miRNA research.

Recently, there have been many advances in the subject of miRNA turnover across various animal and cellular models, primarily through a mechanism termed target-directed miRNA degradation (TDMD)^20–27^. In TDMD, specific target RNAs contain specialized miRNA complementary binding sites - referred to as TDMD triggers within 3ʹ UTRs or non-coding RNAs, that induce rapid degradation of interacting miRNAs^28^. This process is thought to be catalyzed by trigger-mediated AGO conformational change and subsequent recruitment of a Cullin-RING E3 ubiquitin ligase containing ZSWIM8, Dora, or EBAX-1 in vertebrates, *Drosophila*, and *C. elegans*, respectively^23,24,29,30^. The ZSWIM8-containing complex then catalyzes AGO ubiquitination for proteasomal decay and subsequent miRNA degradation, thereby reducing miRNA abundance and activity. We have recently demonstrated the utility of CLASH data in the screening and identification of TDMD triggers, having validated 7 of the 11 currently accepted endogenous triggers in animals^20,26,31^. Given the platform’s comprehensive integration of diverse datasets, CLASHub is well-suited to investigate specific miRNA turnover processes like TDMD. Notably, the platform includes newly added datasets from *ZSWIM8*-knockout cells and tissues, which were not previously available (Supplementary Data 1–4)^13,26,28,32–35^. Leveraging CLASHub, we identified a TDMD trigger for miR-335-3p degradation and several canonical targets for miR-18a-5p. These findings highlight its value as a resource for discovering miRNA regulatory interactions.

## Results

### CLASHub databases

**Fig. 1:**
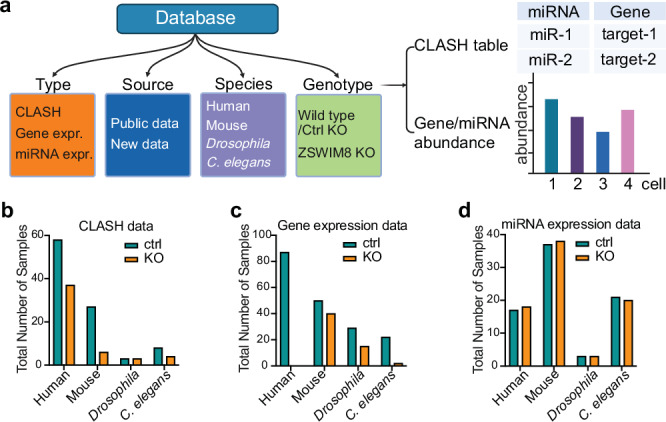
Overview of the datasets integrated in CLASHub. **a** Schematic representation of the CLASHub database composition. The platform integrates three primary data types (orange)—CLASH data, gene expression (expr.) data, and microRNA expression (expr.) data—sourced from both publicly available studies and newly generated datasets in this work (blue). Data span four species (purple): human, mouse, *Drosophila*, and *C. elegans*, and include multiple experimental genotypes (green): wild-type, non-targeting controls, and *ZSWIM8*-knockout samples. The database enables retrieval of CLASH hybrid tables and associated RNA and miRNA abundance information from diverse biological contexts. Bar plots summarizing the number of datasets for CLASH (**b**), gene expression (**c**), and miRNA expression (**d**) across species, with or without knockout of *ZSWIM8* orthologs. Source data are provided as a Source Data file.

CLASHub integrates three main data types for studying miRNA regulation: CLASH data, gene expression data (RNA-seq), and miRNA expression data generated using Accurate Quantification of miRNA by Sequencing (AQ-seq), which improves the accuracy of miRNA quantification by minimizing adapter ligation bias during small RNA library preparation^36^. These datasets were derived from publicly available sources and newly generated data in this study (Fig. 1a). In total, CLASHub aggregates and standardizes CLASH data from diverse studies, incorporating 55 publicly available and 91 newly generated datasets from 25 cell lines/tissues. Among the 17 cell/tissue types covered by newly generated CLASH datasets in this study, 15 have not been profiled by CLASH in prior reports. In contrast to previously reported CLASH data, which mainly focus on wild-type samples, our newly generated datasets significantly expand on *ZSWIM8*-knockout conditions. These datasets span four species—human, mouse, *Drosophila*, and *C. elegans*—and include 52 control samples (wild-type or non-targeting sgRNA) and 39 knockout samples targeting ZSWIM8 orthologs: *ZSWIM8* in humans, *Zswim8* in mice, *Dora* in *Drosophila*, and *ebax-1* in *C. elegans* (Supplementary Fig. 1a–e). For the *ZSWIM8*-knockout cell lines generated in this study, we functionally validated the loss of ZSWIM8 activity by confirming the increase of miR-7, a known TDMD substrate, via Northern blot analysis (Supplementary Fig. 1f). All databases are built on an abundant number of datasets and details of cell lines, tissues, and sample sources are summarized in Fig. 1b–d, Supplementary Fig. 1, and Supplementary Data 1–4.

**Fig. 2:**
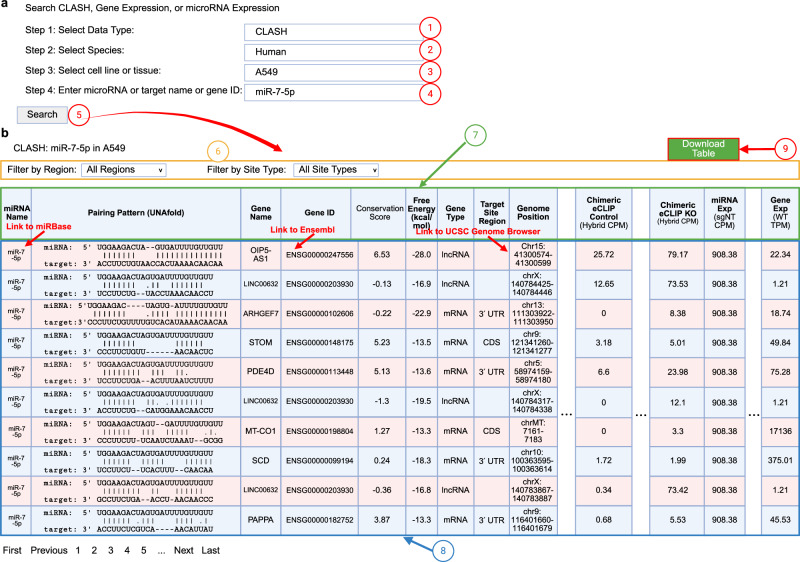
User interface for querying miRNA-target hybrids in the CLASH database. **a** The CLASHub web interface guides users to retrieve CLASH data by selecting (1) data type, (2) species, (3) sample type, and (4) miRNA or gene ID in the query fields, and perform the (5) Search function. **b** The example CLASH output table summarizes detected miR-7 hybrids in A549 cells. Filtering options allow users to refine results by target site region and site type (6). Each row includes comprehensive information regarding the hybrids, such as miRNA name, predicted pairing pattern, etc. (7). Interactive links allow users to directly open miRBase (via miRNA name), Ensembl (via gene ID), and UCSC Genome Browser (via genome position) for each hybrid (8). The Download Table button allows users to retrieve the data table for offline analysis (9). Created in BioRender. https://BioRender.com/2pvvjsd.

To enable convenient access to high-confidence miRNA-target interactions, the CLASH databases feature a user-friendly web interface that allows users to search CLASH data in five intuitive steps (Fig. 2a, #1–#5). Users can select data type (CLASH), followed by species (e.g., Human), and a specific cell line or tissue (e.g., A549). Alternatively, users can select the All Human Sources option, which merges all available cell lines for that species into a single searchable dataset, enabling users to identify miRNA-target interactions across all available human CLASH datasets. Users then enter a miRNA name, gene name, or gene ID of interest (e.g., miR-7-5p). After clicking Search, users are directed to a result page that displays all detected hybrids involving the queried miRNA or gene (Fig. 2b).

The new CLASH data in this database were generated using antibodies specific to different AGO proteins: pan-AGO (AGO1-4) in human and mouse, Ago1 in *Drosophila*, and ALG-1/ALG-2 in *C. elegans*. Additionally, two CLASH methodologies have been utilized, namely qCLASH^26,33^ and chimeric eCLIP^32^ (detailed in the “Methods” section). While qCLASH provides a streamlined, bead-based workflow capable of processing low-input samples, chimeric eCLIP offers the advantage of size selection of hybrids upon SDS-PAGE and transferring of crosslinked RNA–protein complex to the nitrocellulose membrane to reduce non-hybrid background. Another key difference between the two protocols lies in a presumably more efficient 5ʹ adapter ligation step at the cDNA level in chimeric eCLIP^37^. When comparing the normalized unique hybrid yield (Counts Per Million, CPM), chimeric eCLIP libraries with hybrid size selection recovered significantly more hybrids than the gel-free qCLASH method (Supplementary Fig. 1g). Nonetheless, both CLASH datasets yielded abundant hybrid reads and are presented in the CLASHub database in separate columns.

As shown in Fig. 2b (#6–#8), the result table summarizes each miRNA-target hybrid with key features including the miRNA name, base-pairing pattern (predicted by UNAfold^38^), target gene name and ID, conservation score, free energy of the predicted duplex, gene type, target site region (e.g., 5′ UTR, CDS, and/or 3′ UTR), genome position, and target site type (e.g., 8mer, 7mer-m8, 7mer-A1, 6mer, offset 6mer, or non-canonical), which reflect different levels of binding affinity and repression efficacy. Each hybrid is also annotated with the number of replicates in which it was observed under wild-type (WT) and *ZSWIM8*-knockout (KO) conditions, along with corresponding normalized abundance values. Finally, if paired RNA-seq and miRNA-seq data are available, the levels of miRNA and target RNA are also presented (Fig. 2b, #7), allowing users to gauge the relationship between individual RNA abundance and hybrid abundance. To facilitate targeted data exploration, the interface includes filtering options that allow users to refine results based on specific Target Site Region and Target Site Type (Fig. 2b, #6). Additionally, clicking individual cells in the header row allows the users to rank the hybrids based on the specific column of interest (Supplementary Fig. 2).

To illustrate the platform’s utility, we queried miR-7-5p, a highly conserved and neuron-enriched miRNA implicated in tumor suppression and neural development^39–41^ (Fig. 2b, #8). Among the interactions identified in A549 cells, one prominent hybrid involves *OIP5-AS1* (also known as *Cyrano*), a long non-coding RNA previously validated as a TDMD trigger for miR-7 in the mammalian brain^42^. Loss of the *Cyrano* miR-7 binding site has been shown to dramatically elevate miR-7 levels—by 2- to 40-fold—in both cultured cells and murine neuronal tissues, such as the cerebellum^42^. In our dataset, this hybrid is reproducibly detected in three replicates of both control and KO conditions in the chimeric eCLIP data, with a clear increase in normalized abundance upon *ZSWIM8*-knockout (from 25.72 to 79.17, Fig. 2b), consistent with increased miRNA abundance after loss of TDMD. Another notable example is the hybrid between miR-7-5p and *LINC00632*, a locus encompassing ciRS-7, a circular RNA previously described as an efficient miRNA sponge for miR-7^43^. Detection of these interactions demonstrates the ability of CLASHub to reveal non-coding RNA interactions and adds further support to the functional relevance of circular RNAs in miRNA regulation.

Importantly, each row in the results table provides interactive links to external resources, enabling seamless transition to explore detailed information about miRNA, gene, and genomic context (Fig. 2b, #7). Specifically, clicking on the miRNA name, gene ID, or genome position directs the user to the corresponding entries in miRBase (Supplementary Fig. 3a), Ensembl (Supplementary Fig. 3b), and the UCSC Genome Browser (Supplementary Fig. 3c), respectively. To illustrate these link-out functionalities, Supplementary Fig. 3 shows representative examples of the landing pages when querying hsa-miR-7-5p, ENSG00000247556 (*OIP5-AS1*), and chr15:41300574–41300599, respectively. Finally, users can also download the full queried table with a single click (Fig. 2b, #9).

To facilitate interpretation of miRNA-target interactions, CLASHub also integrates expression modules that provide both gene and miRNA abundance profiles across diverse biological contexts, based on RNA-seq and miRNA AQ-seq datasets. 245 RNA-seq datasets from 44 cell lines/tissues and 157 miRNA-seq datasets from 25 cell types were included. This dual capability enables researchers to assess whether a given miRNA-target hybrid occurs in the context of appreciable expression of both the miRNA and its predicted target transcript, information that is typically absent in most existing miRNA-target databases.

**Fig. 3:**
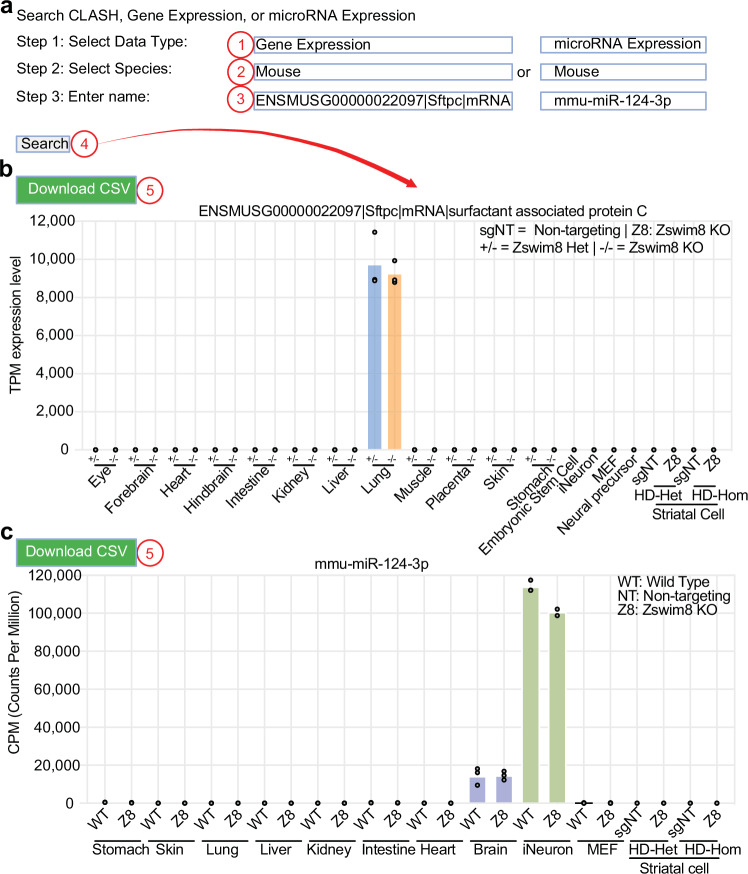
CLASHub gene and miRNA expression database interface and example outputs. **a** User interface for exploring RNA-seq data and miRNA-seq data across tissue and cell types. Users (1) select the Gene Expression or miRNA expression in the data type, (2) specify the species, and (3) enter a gene name, Ensembl gene ID, or miRNA name. (4) Clicking the Search retrieves expression profiles. **b** Example output bar plots showing transcript abundance in transcripts per million (TPM) across mouse tissues and cell types. **c** Example output bar plot showing miRNA abundance in counts per million (CPM) across mouse tissues and cell types. Each dot represents one independent RNA-seq (**b**) or miRNA-seq (**c**) library; For (**b**), *n* = 3 biological replicates per condition for all tissues and for induced neurons (iNeurons) and mouse embryonic fibroblasts (MEFs); *n* = 2 for embryonic stem cell, neural precursor, and each striatal Huntington’s disease heterozygous (HD-Het) or homozygous (HD-Hom) group. For (**c**), *n* = 3 biological replicates per condition for all tissues (Stomach, Skin, Lung, Liver, Kidney, Intestine, Heart, Brain) and for neuron non-targeting control (sgNT); *n* = 2 for Neuron Z8 and each Striatal HD-Het/HD-Hom group; *n* = 6 for MEF sgNT and MEF Z8 (combined from two publicly available datasets). No statistical testing was performed. (5) The Download CSV button allows exporting the data table for offline analysis. Source data are provided as a Source Data file.

Figure 3 illustrates how users can query gene and miRNA expression data. Users first select either Gene Expression or microRNA Expression from the Data Type dropdown menu and enter a gene name, Ensembl gene ID (e.g., *Sftpc*), or a microRNA name (e.g., mmu-miR-124-3p) (Fig. 3a). Clicking Search generates a bar plot displaying transcript or miRNA abundance across tissues and cell types, quantified as transcripts per million (TPM) or counts per million (CPM). For example, querying the surfactant protein C gene shows marked enrichment in lung samples (Fig. 3b), and mmu-miR-124-3p is highly enriched in brain and iNeuron samples, with negligible expression in non-neuronal tissues (Fig. 3c).

In both modules, users can export results by clicking the Download CSV button (Fig. 3b, c, #5). When combined with CLASH hybrid data, these expression profiles provide essential context for evaluating the biological relevance of identified miRNA-target interactions and prioritizing candidates for functional validation.

### CLASHub Analyzer modules

**Fig. 4:**
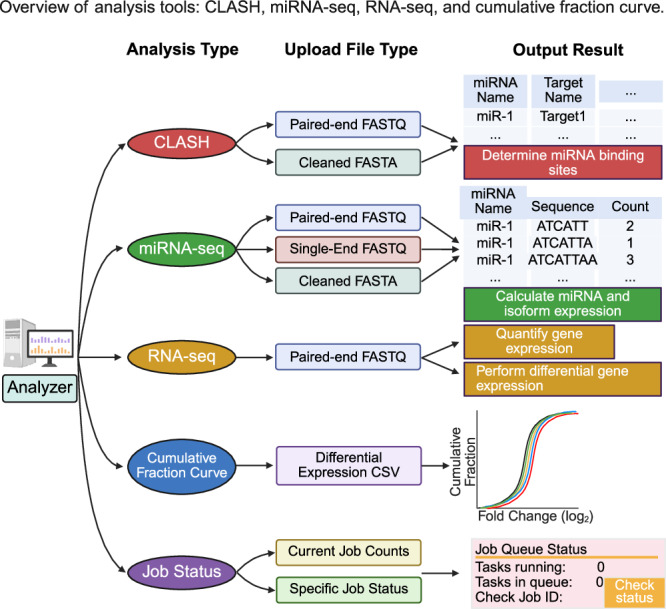
Overview of analysis tools provided by CLASHub. Schematic representation of the five main modules available in the CLASHub Analyzer interface, including CLASH (red), miRNA-seq (green), RNA-seq (orange), cumulative fraction curve generation (blue), and job status monitoring (purple). Users can select the desired analysis type from the left menu. Each module specifies compatible input file formats and outlines the corresponding output content. Created in BioRender. https://BioRender.com/mmat5zk.

**Fig. 5:**
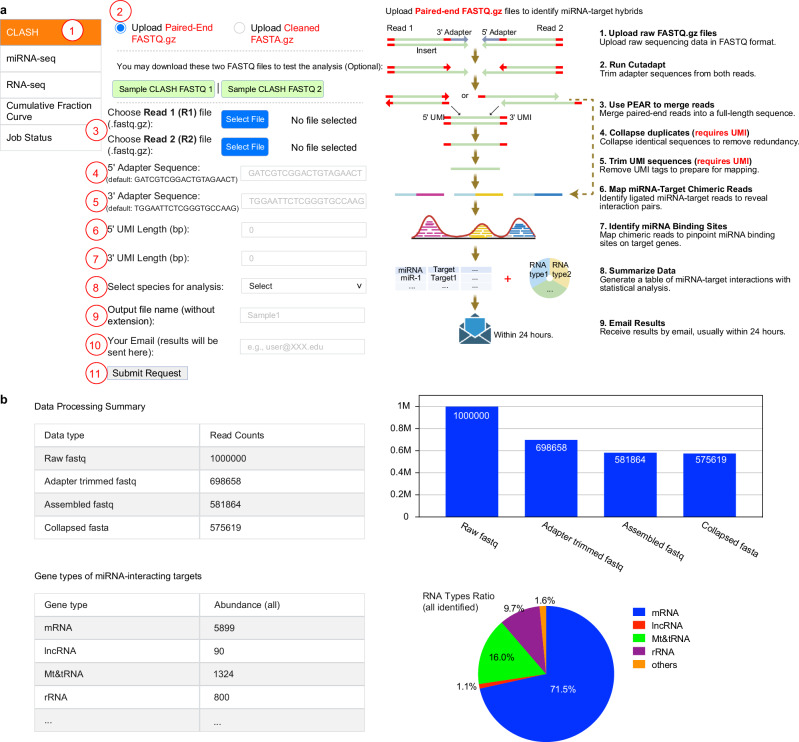
CLASH analysis interface and automated processing pipeline in CLASHub. **a** Interface for initiating CLASH analysis. Users (1) select the CLASH module, (2) select input file type, (3) select local sequencing files for upload, (4 and 5) input adapter sequences, (6–7) define the length of 5′ and 3′ Unique Molecular Identifiers (UMIs), (8) select species, (9) specify output filename, and (10) enter email address, then (11) submit the request. The backend bioinformatic pipeline to process paired-end FASTQ data is shown on the right. Created in BioRender. https://BioRender.com/o8pfkw8. **b** Example output report, displaying processed read counts, RNA-type summary, and RNA composition chart.

Other than summarizing the existing data in databases described above, CLASHub also offers Analyzer modules to analyze new CLASH, miRNA‑seq, and RNA‑seq data provided by the users, to generate a cumulative fraction curve, and to check job status, all accessible via the left menu (Fig. 4). Together, these enable comprehensive miRNA‑network studies across the four model species supported in this resource: human, mouse, *Drosophila*, and *C. elegans*. To perform analysis, users can upload raw or pre‑processed sequencing data, choose the species, input adapter sequences, and output name. The backend custom scripts will automatically process the data and send results to the users by e‑mail. Additionally, the Job Status module allows users to monitor current job counts and query specific job statuses using a unique Job ID, enabling convenient verification of whether a job has completed, is in progress, or remains pending in the queue. All pipelines are fully automated, standardized, and optimized for high‑throughput workflows, and all scripts are available at https://github.com/UF-Xie-Lab/CLASHub; Sample input files for testing individual CLASHub Analyzer modules are available (e.g., Fig. 5a: Sample CLASH FASTQ 1 and 2). The following sections describe each module in detail.

The first Analyzer module is for CLASH analysis, which enables miRNA-target interaction discovery. The CLASH analysis pipeline in CLASHub identifies miRNA-target chimeric hybrids from raw paired-end FASTQ files or pre-processed single-end FASTA files. For paired-end data, uploaded files undergo an automated sequential processing pipeline including adapter trimming (Cutadapt v2.10^44^), read merging (PEAR v0.9.6^45^), deduplication (fastx_collapser v0.0.14), and Unique Molecular Identifier (UMI) trimming (Fig. 5a). Users can specify the 5′ and 3′ UMI lengths in base pairs; when both are set to zero, the deduplication and UMI-trimming steps are automatically skipped. Single-end input files directly proceed to mapping without these initial preprocessing steps (Supplementary Fig. 4a).

Chimeric miRNA-target reads are identified using Hyb^46^, aligned to Ensembl reference transcriptomes using Bowtie2 v2.5.3^47^, and mature miRNA sequences from miRBase Release 22.1. Hybrid free energies (Δ*G*) are calculated using UNAfold v3.8. miRNA-binding sites in the target are annotated with a custom CLASHub.py script, and conservation scores are assessed using phyloP from the UCSC Genome Browser^48^. The outputs include a detailed hybrid interaction table [miRNA-target pairs, genomic locations, base-pairing patterns, Δ*G* values, conservation scores, and transcript annotations (3ʹ UTR or CDS), and Piranha-calculated peak *P*-values, a confidence label to identify high-confidence binding sites] (Supplementary Data 5), as well as an HTML summary report with processing statistics and RNA-type composition (mRNAs, lncRNAs, rRNAs) (Fig. 5b), which also presents a comparative quality assessment visualizing seed type and free energy distributions for high-confidence targets versus excluded background RNAs (Supplementary Fig. 4b). Additionally, CLASHub automatically generates BigWig (bw) files from the mapped non-hybrid reads for visualization. Users may load the bw file into the Integrative Genomics Viewer (IGV) to inspect read coverage at any site of interest. As shown in Supplementary Fig. 4c, the miR-7 binding site on *OIP5-AS1* (*Cyrano*) can be inspected in the human genome. Results are delivered to users by email, typically within 24 h, enabling high-resolution, cross-species exploration of the miRNA-target interaction landscapes.

The second Analyzer module is for miRNA-seq analysis and isoform profiling. The miRNA-seq module of CLASHub quantifies miRNA expression and isoform-specific abundances from user-uploaded sequencing data in formats of paired-end FASTQ, single-end FASTQ, or pre-processed, cleaned FASTA. Paired-end and single-end FASTQ files (Supplementary Figs. 5a and 6a) undergo preprocessing steps similar to those applied in the CLASH analysis, which convert the input files to clean FASTA files (Supplementary Fig. 6b). To support diverse library preparation protocols, users can specify the 5′ and 3′ UMI lengths in base pairs (Supplementary Fig. 5a, Steps 8 and 9). For AQ-seq libraries with 4-nt UMIs at both ends, users enter 4 for each; for standard small RNA-seq libraries lacking UMIs (e.g., Illumina TruSeq or NEBNext kits), users set both values to 0, which instructs CLASHub to automatically skip the deduplication and UMI-trimming steps and proceed directly to miRNA quantification after adapter trimming.

The CLASHub.py script quantifies miRNA levels by perfectly matching the first 18 nucleotides of each trimmed read to the 5′ end of mature miRNA sequences (miRBase Release 22.1), enabling accurate estimation of total miRNA and isoform abundances. The output includes two tables: one for total miRNA abundance (Supplementary Data 6) and one for isoform-level counts that provides the detailed abundance of each miRNA isoform to assess miRNA processing events like tailing/trimming (Supplementary Data 7). A comprehensive HTML report summarizes sequencing read processing metrics, filtering statistics, and graphical visualizations of read distribution along the processing pipeline (adapter trimming, UMI collapse, and read alignment) (Supplementary Fig. 5b).

The third Analyzer module is the RNA-seq module for gene expression quantification and differential expression analyses. CLASHub’s RNA-seq module quantifies gene expression and identifies differentially expressed genes from paired-end FASTQ data. All input reads undergo adapter trimming and genome mapping using HISAT2^49^. For gene quantification, StringTie^50^ is used to calculate normalized transcripts per million (TPM), while gene-level raw counts are extracted via the prepDE.py3 script. Consequently, the workflow outputs both TPM abundance tables and raw count matrices across samples (Supplementary Fig. 7a, Supplementary Data 8–9), alongside comprehensive HTML reports detailing analysis summaries and visualizations (Supplementary Fig. 7b).

For differential expression analysis, users upload paired-end FASTQ files from the Control and Treatment groups. Differential expression is computed using DESeq2^51^ based on the generated raw counts (Supplementary Fig. 8). Results are provided as a differential expression results table containing gene ID, log_2_ fold-changes, and adjusted *P*-values (Supplementary Data 10). The workflow also generates TPM, raw count matrices (similar to Supplementary Data 8–9), and HTML reports (Supplementary Fig. 7b) for all analyzed samples.

Furthermore, to distinguish transcriptional from post-transcriptional regulation and reduce false positives, the RNA-seq Analyzer module incorporates an optional Exon-Intron Split Analysis (EISA) module^52^ (Supplementary Fig. 7a, #10; Supplementary Fig. 8, #23). When enabled, this module performs separate quantification of exonic and intronic reads and classifies each gene’s regulation type (post-transcriptional, transcriptional, or ambiguous) in the EISA output table, as detailed in the “Methods” section.

**Fig. 6:**
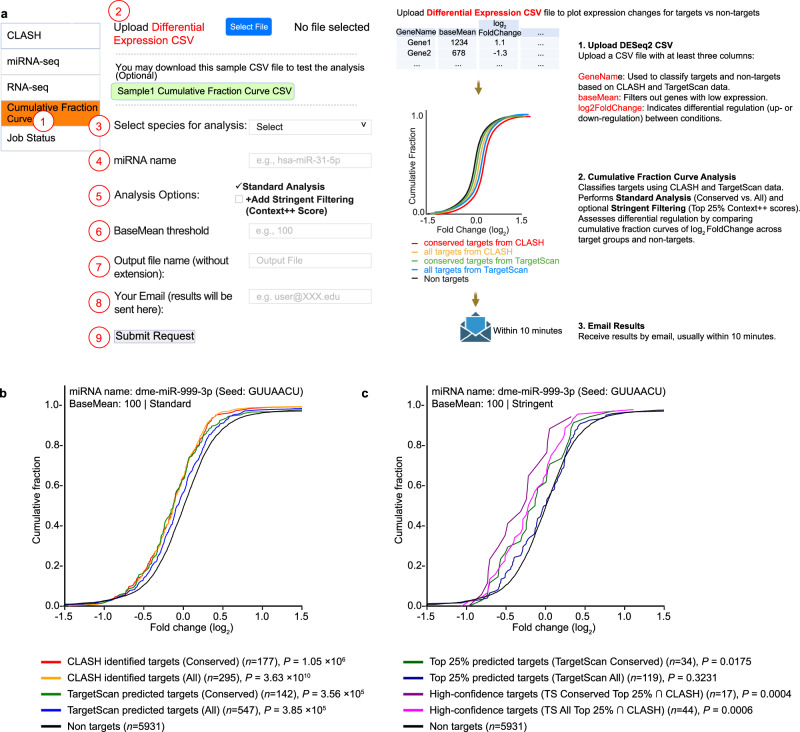
Cumulative fraction curve analysis module. **a** Interface for generating cumulative fraction curves. Users (1) select the module, (2) upload a differential gene expression CSV file, (3) specify species, (4) enter miRNA name, (5) select analysis options (Standard Analysis and/or Stringent Filtering), (6) define optional miRNA count BaseMean threshold, (7) specify output filename, (8) provide email, and (9) submit the request. The backend bioinformatic pipeline to generate the plot is shown on the right. Created in BioRender. https://BioRender.com/2swponf. **b** Example output of the Standard Analysis showing cumulative fraction curves for dme-miR-999-3p targets classified by conservation status (Conserved vs. All) from CLASH and TargetScan datasets. **c** Example output of the Stringent Filtering analysis for the same miRNA, displaying cumulative fraction curves for the top 25% of high-efficacy targets based on TargetScan Context++ scores and overlaps with CLASH data. For both (**b**) and (**c**), *n* denotes the number of genes in each category included in the analysis after BaseMean filtering and overlap with the uploaded differential expression dataset; *P*-values were calculated using two-sided Mann–Whitney *U* tests comparing each target group with non-target genes.

The final Analyzer module is a cumulative fraction curve generator, which evaluates whether miRNA expression changes affect target gene expression relative to non-targets. Users upload differential gene expression CSV files, which can be generated via the RNA-seq differential expression pipeline in the RNA-seq Analyzer module, containing essential columns: GeneName, BaseMean (default threshold: 100), and log_2_FoldChange. Inputs also include species, miRNA name, output filename, and email address (Fig. 6). This module simultaneously compares the distribution of expression changes across multiple target types (CLASH-identified targets, TargetScan-predicted targets, etc.) via a cumulative fraction curve plot and automatically performs Mann–Whitney *U* tests to quantify statistical differences in expression shifts between each target group and non-target genes. The module allows users to choose between a Standard Analysis (grouping targets by conservation status) and a Stringent Filtering mode, which narrows the analysis to the top 25% of high-efficacy targets based on TargetScan Context++ scores^53^. This combined display of CLASH-validated and TargetScan-predicted targets within the same plot robustly supports interpretation of miRNA-mediated regulation by allowing direct comparison of their expression against non-target transcripts.

To illustrate this functionality, Fig. 6b shows the Standard Analysis results for dme-miR-999-3p target genes. This plot shows the overall reduction in miR-999 target expression in cells with high levels of miR-999 compared to the control condition^26^. Furthermore, Fig. 6c demonstrates the Stringent Filtering capability. By focusing on the top 25% of predicted targets with the most favorable context scores, this mode reveals a more pronounced repression pattern. In addition to graphical visualization, the output also includes a downloadable table (Supplementary Data 11) that annotates each gene in the user-provided differential expression file with its target classification (e.g., conserved or all targets from CLASH/TargetScan, top 25% Context++ subsets, high-confidence overlaps, or non-targets). This enables an easy review of how each gene contributes to the observed distributions and facilitates downstream analyses or reporting.

### Insights into miR-335-3p regulation by TDMD using CLASHub

To demonstrate the integrated capabilities of CLASHub as both a curated miRNA-target interaction database and an analysis platform, we applied it to explore miR-335-3p, a miRNA known to be regulated by TDMD^25,27^. Prior studies have consistently shown that miR-335-3p is among the most strongly upregulated miRNAs when *ZSWIM8* is knocked out, indicating its potential regulation by TDMD. Motivated by these findings, we leveraged both the CLASHub database and analysis modules to investigate miR-335-3p expression, potential TDMD triggers, and binding site conservation across species.

**Fig. 7:**
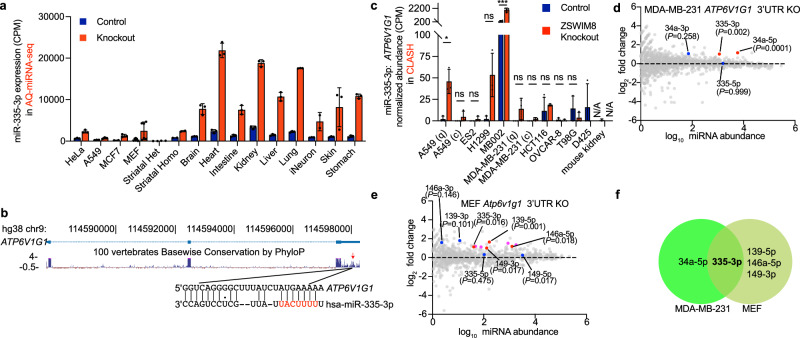
Insights into miR-335-3p regulation and trigger discovery using CLASHub. **a** miR-335-3p levels in *ZSWIM8*-knockout (orange) and control (blue) samples across human cell lines, mouse embryonic fibroblasts (MEFs), and mouse tissues. Expression values are shown as counts per million (CPM) from AQ-seq (Accurate Quantification by sequencing) datasets. Each data point represents one independent AQ-seq library; bars show mean ± SD. Sample sizes were *n* = 3 biological replicates per condition for most groups, except MEF (*n* = 6 per condition), MCF7 (*n* = 2 control, *n* = 3 knockout), Striatal Het and Striatal Homo (*n* = 2 per condition), and iNeuron (*n* = 3 control, *n* = 2 knockout). No statistical testing was performed. **b** UCSC Genome Browser view of the *ATP6V1G1* 3′ UTR on chromosome 9. The miR-335-3p binding site is denoted with a red arrow. PhyloP conservation track highlights conserved regions with blue bars. **c** Normalized abundance (CPM) of miR-335-3p:*ATP6V1G1* chimeric reads across nine human cell lines and mouse kidney, comparing control (blue) and *ZSWIM8*-knockout (orange) conditions. Each point represents one independent CLASH library from a separately cultured cell population; bars show mean ± SD. Biological replicates per condition: *n* = 3 for A549 (qCLASH), A549 (chimeric eCLIP), ES2, H1299, MDA-MB-231 (qCLASH), MDA-MB-231 (chimeric eCLIP), OVCAR8 and T98G; *n* = 4 for MB002; *n* = 5 (control) and *n* = 3 (knockout) for HCT116. D425 and the mouse kidney lack matched knockout libraries (N/A) and were not tested. Each cell line was analyzed independently by an unpaired two-tailed Welch’s *t*-test in GraphPad Prism. *P < 0.05, **P < 0.01, ***P < 0.001; ns not significant. Exact *P*-values for significant comparisons: MB002, *P* = 0.0004; A549 (q), *P* = 0.0263. Differential expression of mature miRNAs in **d** MDA-MB-231 *ATP6V1G1* 3′ UTR knockout cells (*n* = 3 biological replicates per group) and **e** MEF *Atp6v1g1* 3′ UTR knockout cells (*n* = 4 biological replicates per group) compared with controls. Each point represents a mature miRNA, plotted by log_10_ abundance (*x*-axis) and log_2_ fold change (*y*-axis). Differential expression was analyzed using the DESeq2 Wald test (two-sided), and adjusted *P*-values were calculated using the Benjamini–Hochberg method for multiple testing. miRNAs significantly upregulated in knockout samples (adjusted *P*-value < 0.02) are shown in red; corresponding passenger strands are indicated in blue if not significantly changed; miRNAs with concurrent upregulation of both 5p and 3p strands are shown in magenta. **f** A Venn diagram showing overlap of significantly upregulated miRNAs between MDA-MB-231 and MEF cells, upon *ATP6V1G1*/*Atp6v1g1* trigger knockout. Source data are provided as a Source Data file.

Using CLASHub’s miRNA expression datasets, miR-335-3p expression was analyzed across samples from human cell lines to mouse tissues. In *ZSWIM8*-knockout human cells (e.g., HeLa and A549) and *Zswim8*-knockout mouse samples, which include cells such as MEF and tissues like the brain, kidney, lung, and stomach, miR-335-3p expression was 3- to 12-fold higher compared to wild-type or non-targeting knockout controls (Fig. 7a). The consistent response across diverse human and mouse samples highlights miR-335-3p’s universal sensitivity to ZSWIM8/Zswim8 depletion.

We next queried the CLASHub database to systematically identify potential TDMD-inducing chimeric hybrids of all *ZSWIM8*-sensitive miRNAs, including miR-335-3p^20,24,25,27,54^. Candidates were required to contain a canonical seed match and at least eight contiguous nucleotides of complementarity at the miRNA 3′ end (allowing G:U wobble pairs), consistent with established TDMD structural criteria^20,55^ (Supplementary Data 12). We further refined the candidate list by retaining only those hybrids with a 7mer or 8mer seed match located within the 3′ UTR of the target transcript. This filtering strategy yielded seven high-confidence miR-335-3p TDMD candidate hybrids (Supplementary Table 1). Among these, miR-335-3p:*ATP6V1G1* interaction stood out based on several features: First, its base-pairing pattern includes a contiguous 7-nucleotide seed match and 10 consecutive nucleotides of complementarity at the miRNA 3ʹ end (including G:U wobble pairings), a configuration characteristic of TDMD-inducing sites (Fig. 7b and Supplementary Fig. 9a). Second, it exhibited a highly favorable predicted free energy (−23.4 kcal/mol in human; −24.4 kcal/mol in mouse), indicating strong thermodynamic stability. Third, *ATP6V1G1* hybrids were broadly detected across 9 cell lines and 1 tissue, whereas other candidate hybrids were observed in only one to four cell lines/tissues, suggesting that *ATP6V1G1* represents a more robust and broadly relevant TDMD trigger for miR-335-3p. Fourth, miR-335-3p:*ATP6V1G1* exhibited the highest mean hybrid abundance upon *ZSWIM8*-knockout (211.44 CPM), far exceeding all other candidates, and showed a substantial increase from control conditions (27.52 CPM), a pattern consistent with TDMD-mediated regulation (Supplementary Table 1). Statistical testing revealed significant increases in miR-335-3p:*ATP6V1G1* abundance upon *ZSWIM8*-knockout in A549 (qCLASH) and MB002, with a similar trend observed in H1299 (*P* = 0.069). In the remaining cell lines and mouse kidney, the hybrid was detected but at lower levels, and differences did not reach statistical significance, likely reflecting limited chimeric read recovery in those samples (Fig. 7c). Finally, PhyloP conservation analysis across vertebrates demonstrated significant conservation at the seed and 3′ end pairing regions. Notably, while our manuscript is in revision, the miR-335-3p:*ATP6V1G1* interaction was reported by the Bartel group, further supporting its potential relevance as a TDMD trigger^56^.

To experimentally validate *ATP6V1G1*’s involvement in miR-335-3p TDMD, we employed CRISPR/Cas9 to remove the potential TDMD trigger sequence. Three homozygous knockout clones (M1: 108 bp deletion with 1 bp insertion, M5: 118 bp deletion with 6 bp insertion, and M8: 108 bp deletion) were generated in human MDA-MB-231 cells (Supplementary Fig. 9b), and four homozygous knockout clones (b5, b6, b11, and b17, all with identical 65 bp deletions) were generated in mouse MEF cells (Supplementary Fig. 9c). When analyzing the miRNA-seq data in *ATP6V1G1/Atp6v1g1* trigger knockout cells, we applied stringent thresholds (baseMean >40, adjusted *P*-value < 0.02, log_2_ fold change >1) to reduce false positives in low-abundance miRNAs. Specifically, in MDA-MB-231 cells, miR-335-3p and miR-34a-5p were the most prominently elevated (Fig. 7d, red dots), while their corresponding passenger strands were not significantly upregulated (Fig. 7d, blue dots). In MEF cells, miR-335-3p, miR-139-5p, miR-146a-5p, and miR-149-3p showed marked increases (Fig. 7e, red dots), with their passenger strands not significantly upregulated (Fig. 7e, blue dots). Notably, miR-195a and miR-497 exhibited concurrent upregulation of both 5p and 3p strands, suggesting that their apparent changes may be due to increased transcription rather than loss of TDMD (Fig. 7e, magenta dots). Overall, miR-335-3p was the only miRNA upregulated in both human and mouse trigger knockout models, reinforcing the specific role of *ATP6V1G1/Atp6v1g1* as a TDMD trigger (Fig. 7f, Supplementary Data 13–14).

### Identification and validation of miR-18a-5p targets using CLASHub

To further demonstrate the reliability of the miRNA-target interactions identified by CLASHub, we sought to identify high-confidence miR-18a-5p candidate targets that have not been previously reported. All miR-18a-5p CLASH hybrids detected across human cell lines and tissues in the CLASHub database were systematically filtered using the following criteria: First, the hybrid must be detected in at least four independent cell lines or tissues; Second, the interaction must contain an 8mer seed match; Third, the binding site must be located within the 3ʹ UTR of the target mRNA, where miRNA-mediated repression is typically most effective; Lastly, the PhyloP conservation score must be greater than zero, indicating evolutionary constraint at the binding site. This filtering yielded 55 candidate miR-18a-5p targets (Supplementary Data 15). To prioritize interactions, previously validated or published miR-18a-5p targets were excluded, and seven candidates—*ATXN1L*, *FAM3C*, *NEDD4*, *PDE4D*, *RNF4*, *ZBTB4*, and *ZNF367*—were selected for experimental validation in HEK293T cells (Supplementary Table 2).

**Fig. 8:**
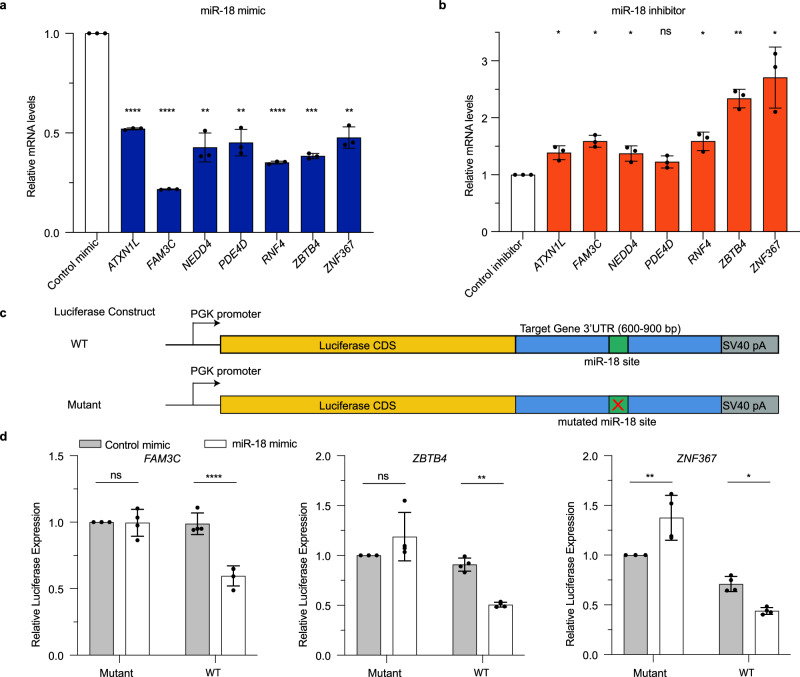
miR-18a-5p regulates the expression of predicted target genes in HEK293T cells. **a** Relative mRNA levels of *ATXN1L*, *FAM3C*, *NEDD4*, *PDE4D*, *RNF4*, *ZBTB4*, and *ZNF367* in HEK293T cells transfected with miR-18a-5p mimic (blue bars) compared to the negative control mimic (Control mimic, white bar), measured by RT-qPCR. Cells were transfected with 22 nM mimic and harvested 48 h post-transfection. mRNA levels were normalized to β-Actin and are expressed relative to the respective control group (set to 1). Data represent the mean ± SD of three independent biological replicates (*n* = 3). Exact *P*-values (paired two-tailed Student’s *t*-test): *ATXN1L*, *P* < 0.0001; *FAM3C*, *P* < 0.0001; *NEDD4*, *P* = 0.0053; *PDE4D*, *P* = 0.0049; *RNF4*, *P* < 0.0001; *ZBTB4*, *P* = 0.0002; *ZNF367*, *P* = 0.0035. **b** Relative mRNA levels of the target genes in HEK293T cells transfected with miR-18a-5p inhibitor (red bars) compared to the negative control inhibitor (Control inhibitor, white bar), measured by RT-qPCR. Cells were transfected with 44 nM inhibitor and harvested 48 h post-transfection. mRNA levels were normalized to β-Actin and are expressed relative to the respective control group (set to 1). Data represent the mean ± SD of three independent biological replicates (*n* = 3). Exact *P*-values (paired two-tailed Student’s *t*-test): *ATXN1L*, *P* = 0.0322; *FAM3C*, *P* = 0.0104; *NEDD4*, *P* = 0.0412; *PDE4D*, *P* = 0.0666 (ns); *RNF4*, *P* = 0.0243; *ZBTB4*, *P* = 0.0050; *ZNF367*, *P* = 0.0312. **c** Schematic representation of the luciferase reporter constructs used for validation. The wild-type (WT) construct contains the full-length 3′ UTR (600–900 bp) of the target gene fused downstream of the Firefly luciferase coding sequence (CDS), driven by the phosphoglycerate kinase (PGK) promoter. The mutant construct harbors specific nucleotide substitutions within the predicted miRNA seed-binding region (indicated by the red cross) to disrupt miRNA interaction. An SV40 poly(A) signal (SV40 pA) is included downstream of the 3′ UTR to ensure proper transcript processing. **d** Dual-luciferase reporter assays verifying the direct targeting of *FAM3C*, *ZBTB4*, and *ZNF367* by miR-18a-5p. HEK293T cells were co-transfected with luciferase reporter plasmids containing either wild-type (WT) or mutant 3′ UTR sequences of the indicated genes, together with miR-18a-5p mimic (white bars) or negative control mimic (gray bars). The mutant constructs contained point mutations in the predicted miR-18a-5p seed-binding regions. Luciferase activity was assessed 48 h post-transfection. Firefly luciferase activity was normalized with the Renilla luciferase activity. Data are presented as mean ± SD of four independent biological replicates (*n* = 4). Statistical analysis was performed using two-way ANOVA with Šídák’s multiple comparisons test. Exact *P*-values for mimic vs. control comparisons within each construct: *FAM3C* WT, *P* < 0.0001; *FAM3C* Mut, *P* = 0.9923 (ns); *ZBTB4* WT, *P* = 0.0014; *ZBTB4* Mut, *P* = 0.1098 (ns); *ZNF367* WT, *P* = 0.0154; *ZNF367* Mut, *P* = 0.0017. *P < 0.05, **P < 0.01, ***P < 0.001, ****P < 0.0001; ns not significant. Source data are provided as a Source Data file.

To assess whether modulating miR-18a-5p levels could alter the mRNA abundance of these targets, HEK293T cells were transfected with a miR-18a-5p mimic, which resulted in significant downregulation of all seven candidate genes compared to the negative control mimic (Fig. 8a). Conversely, inhibiting endogenous miR-18a-5p with an antisense inhibitor led to a reciprocal upregulation of *ATXN1L*, *FAM3C*, *NEDD4*, *RNF4*, *ZBTB4* and *ZNF367*, while the increase in *PDE4D* levels did not reach statistical significance (Fig. 8b). These data indicate that miR-18a-5p functions as a repressor for these genes, consistent with the CLASH-identified interactions.

To determine whether this regulation is mediated through direct binding to the 3ʹ UTRs as predicted, dual-luciferase reporter assays were performed on three representative targets: *FAM3C*, *ZBTB4*, and *ZNF367*. Luciferase reporter vectors were generated containing either the wild-type (WT) 3ʹ UTRs or mutant (Mut) 3ʹ UTRs in which the predicted miR-18a-5p seed-binding sites were replaced with their reverse complement sequences (Fig. 8c; see “Methods” section). Co-transfection of the miR-18a-5p mimic with the WT reporters significantly suppressed luciferase activity for all three genes (Fig. 8d). Importantly, this suppressive effect was abrogated or reversed in the mutant reporters (Fig. 8d), confirming that miR-18a-5p directly regulates *FAM3C*, *ZBTB4*, and *ZNF367* via the specific binding sites identified in the CLASHub dataset. Collectively, these results provide strong experimental support for the accuracy of the miRNA-target interactions cataloged in CLASHub.

## Discussion

Multiple high-throughput experimental methods, such as HiTS-CLIP, PAR-CLIP, CLASH, and Chimeric eCLIP, have been developed to identify miRNA-binding sites^11–13,32^. However, existing studies are often limited to a narrow range of cell lines and tissues, including human cell lines such as HCT116, HEK293T, Huh-7.5, TIVE-EX-LTC, as well as mouse cell lines like HE2.1B, 3T12, and cortex tissues, among others^13,32–35^. In this study, beyond the incorporation of publicly available CLASH data, additional datasets from a diverse range of cancer cell lines and tissues were utilized or generated, including human cell lines (e.g., A549, D425, ES2, HepG2, H1299, MB002, MDA-MB-231, OVCAR8, T98G, U87MG, and 501Mel), mouse samples (e.g., MEF, striatal cells, heart tissue, and kidney tissue). The expanded scope of CLASH data includes both control samples (e.g., wild type or non-targeting sgRNA) and *ZSWIM8*-knockout samples, which are essential for studying TDMD^20,23,25,27,57–59^. Furthermore, gene expression and miRNA expression data from publicly available datasets have been re-analyzed and integrated into the CLASHub database. These datasets provide valuable insights and enable researchers to efficiently explore miRNA-target interactions across various experimental conditions.

In addition to its database functionality, CLASHub offers an Analyzer interface, a graphical user interface that simplifies miRNA-related data analysis. Unlike previous tools, such as Hyb, which identify miRNA-target hybrids from CLASH FASTQ files but lack advanced data processing^46^, the CLASHub Analyzer automates the entire analytical workflow. A recently developed Hybkit provides a powerful Python-based toolkit for hybrid data analysis, but it requires coding skills and is less accessible to non-programmers^60^. In contrast, CLASHub allows users to upload their own FASTQ or FASTA files, and the platform processes the data, providing results via email. These results include miRNA-target hybrid abundances, miRNA-binding site conservation scores, and miRNA-target pairing patterns. Beyond CLASH analysis, the platform also supports miRNA-seq and RNA-seq analyses, as well as cumulative fraction curve analyses based on miRNA-regulated differential gene expression. By offering these integrated features, CLASHub significantly reduces the time and computational expertise required for miRNA-related analyses, allowing researchers to focus on biological interpretation. Besides, researchers lacking bioinformatic expertise but needing a tool to analyze their raw sequencing data can benefit from RNA-seq and differential expression analyses, which are broadly applicable.

### CLASHub enables the discovery of TDMD triggers and canonical miRNA targets

miR-335-3p has been previously reported to play critical roles in memory function in the brain^61^. Additionally, it functions as a tumor suppressor in several cancers and targets LRRK2 to reduce inflammation, potentially mitigating Parkinson’s disease progression^62^. These findings underscore the significant roles of miR-335-3p in neurological and cancer-related pathways. Furthermore, prior studies have demonstrated that miR-335-3p is regulated by *ZSWIM8*-mediated TDMD in various mouse tissues^27^; however, the specific trigger responsible for miR-335-3p degradation has remained unclear.

Using the CLASHub platform, a potential trigger for miR-335-3p degradation was identified in the 3ʹ UTR of *ATP6V1G1*. This finding was further validated through the CLASHub platform’s Analyzer function, where miRNA-seq analysis was employed to assess miRNA abundance changes in *ATP6V1G1* trigger knockout samples. These analyses confirmed that the identified *ATP6V1G1* trigger plays a role in miR-335-3p degradation. Notably, miR-335-3p abundance increased approximately two-fold upon *ATP6V1G1/Atp6v1g1* deletion in MDA-MB-231 and MEF cells (Fig. 7d, e), whereas *ZSWIM8*-knockout in MEF cells resulted in a ~7-fold upregulation (Fig. 7a), suggesting that additional TDMD triggers likely contribute to miR-335-3p turnover.

Beyond our characterization of *ATP6V1G1*/*Atp6v1g1*, the CLASHub database is further evidenced by its ability to independently capture other TDMD triggers that were reported concurrently with this study. For instance, our systematic search identified *Plagl1* and *Malat1*, which were recently described as triggers for the miR-322/503 cluster in mice^56,57^, as well as the *tts-2* lncRNA, a potent trigger for the miR-37/38 family in *C. elegans*^58^ (Supplementary Data 12). The fact that CLASHub harbors clear evidence for these diverse regulatory elements underscores its utility as a rich resource for uncovering conserved mechanisms of miRNA turnover.

In addition to TDMD, CLASHub also demonstrates high precision in identifying canonical miRNA targets. Our experimental validation of miR-18a-5p targets—including *FAM3C*, *ZBTB4*, and *ZNF367*—confirms that the platform effectively filters false positives and captures biologically relevant repression events (Fig. 8). This dual capability to resolve both miRNA turnover (via TDMD) and target repression mechanisms establishes CLASHub as a versatile tool for comprehensive RNA regulation studies.

The development of CLASHub provides a practical framework for simplifying RNA research workflows. It facilitates both exploratory and in-depth analyses, addressing limitations in existing tools and providing a more comprehensive platform for researchers. This study demonstrates the potential of CLASHub to serve as a stepping stone toward a deeper understanding of miRNA landscapes in various biological contexts. Looking forward, we recognize the potential of Large Language Models (LLMs) to revolutionize how researchers interact with complex biological data. While CLASHub currently relies on structured queries, we envision integrating LLM-driven interfaces in future updates. This would allow users to perform natural language–based exploratory analyses (e.g., find high-confidence repressive targets for miR-7 and distinguish them from potential TDMD triggers) and synthesize insights across our multi-dimensional datasets, thereby significantly lowering the technical barrier for discovering miRNA regulatory networks. To further enhance CLASHub’s utility, community feedback and suggestions for new features are welcome to guide its continuous development.

## Methods

### Cell culture and transfection

HEK293T cells were cultured in 6-well plates at a density of 5 × 10^5^ cells per well until they reached approximately 50% confluence. Transfections of miRNA mimic and inhibitor for mRNA expression analysis were performed using Lipofectamine RNAiMAX (Thermo Fisher Scientific) according to the manufacturer’s protocol. Specifically, for miRNA mimic experiments, cells were transfected with 50 pmol of hsa-miR-18a-5p mimic (Catalog #MC12973, mirVana™) or miRNA Mimic Negative Control #1 (Catalog #4464058) to a final concentration of 22 nM. For miRNA inhibitor experiments, cells were transfected with 100 pmol of hsa-miR-18a-5p inhibitor (Catalog #MH12973, mirVana™) or miRNA Inhibitor Negative Control #1 (Catalog #4464076) to a final concentration of 44 nM. Cells were harvested 48 h post-transfection for subsequent RNA extraction.

### Plasmid construction and dual-luciferase reporter assay

The 3ʹ UTR fragments of human *FAM3C*, *ZBTB4*, and *ZNF367* containing predicted miR-18a-5p binding sites were amplified and cloned into the pmirGLO Dual-Luciferase miRNA-Target Expression Vector using the NEBuilder HiFi DNA Assembly method (linearized with *Sac*I and *Xba*I). Mutant constructs with disrupted miR-18a-5p seed regions were generated via overlap extension PCR. The sequences of all oligonucleotides used for cloning and mutagenesis are listed in Supplementary Data 16. All constructs were verified by Sanger sequencing.

For the luciferase reporter assay, HEK293T cells were seeded in 24-well plates (1 × 10^5^ cells/well). Cells were co-transfected with 200 ng of the indicated wild-type or mutant plasmid and 12 pmol of miR-18a-5p mimic or negative control using Lipofectamine 3000 (Invitrogen). Luciferase activity was measured 48 h post-transfection using the Dual-Luciferase® Reporter Assay System (Promega, E1910). Firefly luciferase activity was normalized to Renilla luciferase activity to control for transfection efficiency.

### RNA extraction and quantitative real-time PCR (RT-qPCR)

Total RNA was extracted from cells 48 h post-transfection using TRIzol (cat# 15596018, Invitrogen), according to the manufacturer’s instructions. Then RNA was reverse transcribed to cDNA using the ABScript III RT Master Mix for qPCR with gDNA Remover (cat# RK20429, ABclonal). RT-qPCR was then performed to assess the expression levels of *ATXN1L*, *FAM3C*, *NEDD4*, *PDE4D*, *RNF4*, *ZBTB4*, and *ZNF367* using SsoAdvanced Universal SYBR Green Supermix (cat# 1725274, Bio-Rad). β-Actin was used as the endogenous control for normalization. Relative gene expression was calculated using the 2^−ΔΔCt^ method. All primers used for RT-qPCR are listed in Supplementary Data 16.

### Statistical analysis

Data are presented as mean ± SD. The RT-qPCR data represent three independent biological replicates (*n* = 3), while the Dual-Luciferase reporter assay data represent four independent biological replicates (*n* = 4). Statistical significance was determined using a paired two-tailed Student’s *t*-test for qPCR analysis, and Two-way ANOVA with Šídák’s multiple comparisons test for dual-luciferase reporter assays. CLASH chimeric read abundance comparisons between control and *ZSWIM8*-knockout conditions were analyzed by unpaired two-tailed Welch’s *t*-test, performed independently for each cell line. Cumulative fraction curve analyses used two-sided Mann–Whitney *U* tests. *P*-values are indicated as follows: *P < 0.05, **P < 0.01, ***P < 0.001, ****P < 0.0001, and ns denotes not significant.

### Generation of *ZSWIM8*-knockout cell lines

*ZSWIM8*-knockout (KO) cell lines (including A549, H1299, MDA-MB-231, ES2, OVCAR8, T98G, 501Mel, MB002, and HCT116) were generated using the CRISPR-Cas9 system. Single-guide RNAs (sgRNAs) were cloned into the pLentiCRISPR v2 vector. For *ZSWIM8* targeting, we used two sgRNA sequences validated in a previous study: sgZSWIM8-1: 5’-CAAGTGAGATGAGTACCATG-3’, sgZSWIM8-2: 5’-GTGATTGAGAACGTCAAGCG-3’^23^. For control cell lines, a non-targeting scramble sgRNA was used: sgNonTarget: 5’-ATCGTTTCCGCTTAACGGCG-3’. Lentiviral particles were produced in HEK293T cells, and target cells were transduced and selected with puromycin for 3–5 days to generate stable pools. Knockout efficiency was functionally validated by assessing the specific upregulation of miR-7 via Northern blotting (Supplementary Fig. 1). Note: A375 was used for validation only (Not included in CLASH dataset).

### Generation of ***ATP6V1G1/Atp6v1g1*** TDMD trigger knockout cell lines

To delete the miR-335-3p TDMD trigger region within the 3′ UTR of human *ATP6V1G1* and mouse *Atp6v1g1*, two pairs of sgRNAs flanking each trigger site were designed and cloned into the pLentiCRISPR v2 vector. For human MDA-MB-231 cells, the sgRNA guide sequences were: hATP6V1G1-TDMD-sg1: 5′-AACACTTTCTTGAAGGTCAG-3′ and hATP6V1G1-TDMD-sg2: 5′-GTTAGAACAGTGAATACTAG-3′. For mouse MEF cells, the sgRNA guide sequences were: mAtp6v1g1-TDMD-sg1: 5′-TCTCCTTTCTTACAGGTCAG-3′ and mAtp6v1g1-TDMD-sg2: 5′-AGGCTAGAGTACTCTAAGGC-3′. The same non-targeting scramble sgRNA described above was used as a control. Lentiviral particles were produced in HEK293T cells, and target cells were transduced and selected with puromycin as described above. Single-cell clones were then isolated and expanded for approximately one month to ensure clonal purity. Genomic deletions were confirmed by genotyping PCR using the following primers: for MDA-MB-231, 5′-CCTATGTCTTTTGGCTCAAGCAAC-3′ and 5′-TGGTTGTCACGGAGACAGGG-3′ (wild-type amplicon: 426 bp); for MEF, 5′-TGTCGCCTGTTTTCCTGTGT-3′ and 5′-GAGCCAGGAGGGTGAAGAG-3′ (wild-type amplicon: 375 bp). Deletions were further verified by Sanger sequencing (Supplementary Fig. 9b, c). Three homozygous knockout clones (M1, M5, and M8) were obtained from MDA-MB-231, and four homozygous knockout clones (b5, b6, b11, and b17) were obtained from MEF cells.

### CLASH experiment

CLASH experiments performed in this study followed protocols adapted from qCLASH^26,33^ or chimeric eCLIP^32^, as specified in Supplementary Data 1. Briefly, cells were UV crosslinked on ice at 254 nm with a total energy of 400 mJ/cm² to covalently link RNA–protein complexes.

For qCLASH, ~200 µL cell pellet was lysed in 1 mL cold lysis buffer on ice for 15 min, followed by incubation at 37 °C for 5 min with shaking at 1000 rpm. Lysates were centrifuged at 21,000 × *g* for 15 min at 4 °C. Subsequently, 3 mg of protein lysate underwent overnight immunoprecipitation at 4 °C using either 40 µL of Protein L beads (Thermo Fisher, PI88850) conjugated with approximately 80 µg anti-AGO2 4F9 antibody (human and mouse samples) or 200 µL of Protein A beads (Life Technologies, #10002D) pre-bound with 20 µg anti-*Drosophila* Ago1 antibody (Abcam, ab5070). Immunocomplexes were washed three times with 950 µL lysis buffer, treated with 150 µL of 15 ng/mL RNase A for 12 min at 22 °C, and sequentially washed three times with 950 µL of each of the following buffers: 1× PXL, 5× PXL, High Stringency Buffer, High Salt Buffer, and PNK buffer (buffer compositions and reaction mixes are listed in Supplementary Data 17). RNAs on beads were phosphorylated with 80 µL T4 PNK mix at 16 °C for 40 min, washed three times with 950 µL PNK buffer, and subjected to intermolecular ligation using 500 µL Intermolecular Ligation Mix overnight at 4 °C. Following ligation, beads underwent three washes with 950 µL 1× PNK buffer, were treated with 80 µL dephosphorylation mix at 16 °C for 40 min with intermittent shaking (15 s every 1 min at 1200 rpm), washed twice with 950 µL 1× PNK-EGTA and three times with 950 µL 1× PNK buffer, and incubated overnight at 16 °C in 80 µL 3′-adapter ligation mix with intermittent shaking (15 s every 1 min at 1400 rpm) (adapter sequences are listed in Supplementary Data 17). After washing three times with 950 µL 1× PNK buffer, protein complexes were eluted by incubation with 100 µL protein elution buffer at room temperature for 15 min with continuous shaking at 1400 rpm. Eluted AGO complexes were digested with 50 µL proteinase K mix at 37 °C for 20 min. After reaction, RNA was extracted by Phenol:Chloroform:Isoamyl Alcohol (PCA) with ethanol precipitation, phosphorylated with 4.5 µL T4 PNK mix at 16 °C for 40 min. The reaction was extracted again with PCA and precipitated with ethanol, and resuspended into 10 µL H₂O. For 5′ adapter ligation, RNA was mixed with 10 µL of 5′ adapter ligation mix overnight at 16 °C. RNA was then purified with PCA and precipitated with ethanol, resuspended in 11 µL H₂O, mixed with 1 µL 10 µM RTP and 1 µL 10 µM dNTPs, incubated at 65 °C for 5 min, and subjected to reverse transcription using RT mix.

For chimeric eCLIP, cell lysates were prepared in 1 mL ice-cold iCLIP lysis buffer supplemented with 20 μL Murine RNase Inhibitor (NEB, M0314) (buffer compositions and reaction mixes are listed in Supplementary Data 18). Samples were sonicated using a Bioruptor (low setting, 4 °C, 5 min, 30 s on/off cycles), treated with 10 μL Turbo DNase (Invitrogen, AM2239) and 20 μL 1:100 diluted RNase I, and incubated at 37 °C for 5 min at 1200 rpm. Lysates were cleared by centrifugation (21,000 × *g*, 10 min, 4 °C). Immunoprecipitation was performed with 40 µL Protein L magnetic beads conjugated with 80 µg anti-AGO2 4F9 antibody (human and mouse), incubated overnight at 4 °C with rotation. Beads were washed thrice each with 500 µL cold High Salt Wash Buffer and Wash Buffer, then once with 200 µL cold 1× PNK buffer. Phosphorylation on beads used 100 µL T4 PNK Minus Mix at 37 °C for 20 min, followed by one wash with 500 µL High Salt Wash Buffer and three washes with 500 µL Wash Buffer. Intermolecular ligation was conducted overnight at 4 °C using 180 µL Intermolecular Ligation Mix. Beads were then washed three times each with 500 µL cold High Salt Wash Buffer and Wash Buffer. FastAP treatment was performed at 37 °C for 10 min with 50 µL FastAP mix. To the beads with FastAP mix, 150 µL PNK mix was added, followed by 20 min incubation at 37 °C. This mixture was removed from the beads, followed by one wash with 500 µL High Salt Wash Buffer and three washes with 500 µL Wash Buffer. For 3′ linker ligation, beads were resuspended in 80 µL 3′ adapter ligation mix and incubated overnight at 16 °C with intermittent shaking (15 s every 1 min, 1400 rpm). Then four washes followed (once 500 µL cold Wash Buffer, once 500 µL High Salt Wash Buffer, and twice with 500 µL Wash Buffer). After 3′ linker ligation and washing, samples were processed by one of two approaches. In the first approach, samples were denatured in 7.5 µL 4× LDS buffer, 3 µL 1 M DTT, and 20 µL Wash Buffer at 70 °C for 10 min, separated on NuPAGE gels, transferred to nitrocellulose membranes (Amersham Protran 0.45 µm), and a region from 100–250 kDa was excised and digested with 150 µL Proteinase K SDS mix at 37 °C for 20 min, followed by an additional 20-min incubation at 50 °C. In the second approach, protein complexes on beads were directly digested with Proteinase K mix at 37 °C for 20 min without gel separation. In both cases, RNA was recovered by PCA extraction and ethanol precipitation and subsequently resuspended in 9 µL nuclease-free water. For reverse transcription, 1 µL of 10 mM dNTPs and 0.5 µL of RTP primer were added to the RNA, incubating the mixture at 65 °C for 3 min, then immediately placing it on ice. After adding 10 µL RT mix, the reaction was incubated at 55 °C for 20 min. Following reverse transcription, cDNA was treated with 2.5 µL ExoSAP-IT (Thermo Fisher, 78201.1) at 37 °C for 15 min. The reaction was subsequently mixed with 1 µL 0.5 M EDTA, treated with 3 µL 1 M NaOH at 70 °C for 10 min, and neutralized using 3 µL 1 M HCl. The cDNA was then purified by binding to 5 µL Silane beads (Invitrogen, 37002D) in the presence of 90 µL RLTW buffer and 108 µL 100% ethanol at room temperature for 10 min. The beads underwent two washes with 300 µL of 80% ethanol, followed by a final wash with 150 µL of 80% ethanol. Beads were air-dried for 5 min prior to the subsequent ligation step. For the 5′ adapter ligation, cDNA was initially denatured with 1.45 µL TT Elution Buffer, 0.5 µL RA5 DNA adapter (100 µM), and 0.8 µL DMSO at 70 °C for 2 min. Following denaturation, 7.55 µL of 5′ adapter ligation mix was added, and reactions were incubated overnight at room temperature with rotation. Post-ligation, cDNA was purified via a second silane bead cleanup (using 2.5 µL beads with 45 µL RLTW buffer and 45 µL ethanol), washed as described previously, and eluted into 22 µL Bead Elution Buffer.

For both qCLASH and chimeric eCLIP methods, cDNA libraries were PCR-amplified for 12 cycles using RPI1 and RPIX primers. Amplified libraries were size-selected on 8% polyacrylamide gels to enrich fragments between 180 bp and 400 bp. Sequencing was performed using Illumina NovaSeq platforms. Detailed reagent compositions and oligonucleotide sequences for qCLASH and chimeric eCLIP are provided in Supplementary Data 17–18.

### CLASH analysis

The CLASH Analyzer module allows users to upload paired-end FASTQ files or pre-processed, cleaned FASTA files to the web server. For paired-end data, adapters were trimmed with Cutadapt v2.10 using the default 5′ adapter GATCGTCGGACTGTAGAACT and 3′ adapter TGGAATTCTCGGGTGCCAAG^44^. The trimmed paired-end reads were then merged with PEAR v0.9.6^45^, and when UMIs are present (i.e., when either the 5′ or 3′ UMI length is set to a value greater than zero), PCR duplicates were collapsed using fastx_collapser v0.0.14, and UMIs were trimmed from both ends using Cutadapt with user-specified lengths to generate a cleaned FASTA file. When both UMI lengths are set to zero, the collapsing and UMI-trimming steps are bypassed, and reads proceed directly to length filtering. Hybrid miRNA–target reads were identified with Hyb^46^. Within Hyb, reads were mapped by Bowtie2 v2.5.3 to Ensembl reference transcriptomes (Release 115) together with mature miRNA sequences from miRBase Release 22.1^47^. UNAfold v3.8 was used to calculate miRNA–target hybrid free energies (Δ*G*) and base-pairing patterns. In parallel, the cleaned FASTA reads were aligned to the corresponding species’ genome using HISAT2 and sorted with SAMtools v1.21^49,63^. The resulting BAM files were converted to BED format using BEDTools v2.31.1^64^. To enable genome-browser visualization, BED files were further converted into bedGraph and subsequently into bigWig format using bedGraphToBigWig v2.10. These bigWig files allow users to inspect the CLASH read coverage around miRNA–target interaction sites directly in genome browsers such as the Integrative Genomics Viewer (IGV).

To assess target site confidence, BED files were analyzed with Piranha v1.2.1 for peak-calling^65^. Piranha was run with a bin size of 50 bp and a background threshold of 0.9 (i.e., the lowest 90% of bins were used to model background read distributions). Under these settings, Piranha fits a zero-truncated negative-binomial model to estimate background counts and assigns a *P*-value to each genomic bin; adjacent significant bins were merged into contiguous peaks. After peak calling, a custom Python script (CLASHub.py) compared each miRNA–target chimeric site with the Piranha peaks. Because bin-based statistical peak callers often generate peak boundaries that do not precisely match nucleotide-resolution binding sites, each Piranha peak was expanded by ±50 bp before overlap evaluation. This buffer accounts for bin-edge uncertainty and ensures that genuine miRNA-binding hotspots located near bin boundaries are not missed. The script assigned the best-supported peak (lowest *P*-value) to each site where available.

To minimize false positives for database presentation, interactions were classified as high-confidence if they mapped to Ensembl-annotated mRNA, lncRNA or lincRNA, and satisfied at least one of the following criteria: (1) canonical seed pairing (8mer, 7mer, 6mer, or offset 6mer); OR (2) significant Piranha peak support (*p* < 0.01) coupled with specific structural features of the miRNA-target base-pairing^66^, including a continuous seed match ≥4 nt, extensive 3′ pairing ≥10 nt, and a geometric offset (nucleotide number difference in unpair region in target and miRNA) between −4 and +6 nucleotides. Conservation scores for the binding sites were retrieved from phyloP via the UCSC Genome Browser^48^.

### CLASH database construction

In contrast to the Analyzer module, which processes user-submitted data on demand, the static CLASH database is built from a curated set of pre-processed datasets subjected to uniform quality thresholds. To ensure statistical robustness and minimize bias from low-coverage samples, only datasets exceeding 5000 unique miRNA–target hybrids per sample were included. Additionally, each represented cell line or tissue was required to have at least two independent biological replicates to ensure reproducibility. All datasets—both previously published and newly generated—were processed through the same standardized CLASHub Analyzer pipeline prior to database integration, ensuring consistent handling across studies and preventing cross-study batch effects. Only high-confidence miRNA–target interactions, as defined above in the “CLASH analysis” section, were retained for display, and a thermodynamic threshold (Δ*G* < − 11.1 kcal/mol) was applied to all retained interactions based on established standards^14^. Users seeking access to the complete, unfiltered set of miRNA–target hybrids can do so by processing raw data through the Analyzer module.

### miRNA sequencing experiments

miRNA libraries were prepared using a modified AQ-seq protocol^36^. Total RNA was extracted from MDA-MB-231 and MEF cells (either non-targeting sgRNA controls or *ATP6V1G1*/*Atp6v1g1* TDMD region knockouts) using TRIzol reagent (Thermo Fisher Scientific), followed by isopropanol precipitation. For each sample, 15 μg of total RNA was mixed with 1 μL of 3.33 nM synthetic spike-in RNA pool containing 30 distinct sequences, and the mixture was resolved on 15% urea–polyacrylamide gel. RNA fragments of ~18–30 nt were excised, eluted overnight in 800 μL of 0.3 M NaCl at 25 °C with shaking (1400 rpm), and ethanol-precipitated by adding 40 μL 3 M NaOAc (pH 5.2), 0.5 μL GlycoBlue, and 1 mL pre-chilled 100% ethanol. RNAs were resuspended in 3 μL RNase-free water. For 3′ adapter ligation, 3 μL of purified RNA was combined with 0.5 μL of 5 μM miRCat-33 3′ adapter, heated at 70 °C for 2 min, and transferred to ice. Then, 6.5 μL of 3′ ligation mix was added, and the reaction was incubated at 25 °C for 4.5 h. Ligation products (~45–57 nt) were size-selected on 15% urea–PAGE and eluted overnight in 730 μL 0.3 M NaCl. The eluted RNA was ethanol-precipitated as above and resuspended in 2.5 μL of 5′ adapter mastermix (2.14 μL RNA, 0.36 μL of 5 μM 5′ adapter). The mixture was denatured at 70 °C for 2 min and chilled on ice. Subsequently, 7.5 μL of 5′ ligation mix was added, followed by a 1-h incubation at 37 °C. For reverse transcription, 10 μL of the 5′ and 3′ adapter-ligated RNA was mixed with 1 μL of 4 μM RT primer (RTP), heated at 70 °C for 2 min, and placed on ice. A 9 μL RT mix was then added. The reaction was incubated at 50 °C for 1 h, then heat-inactivated at 70 °C for 15 min, and held at 4 °C. cDNA was amplified using RP1 and RPIX primers with 12 PCR cycles. PCR products were resolved on 8% native PAGE, and miRNA insert-sized fragments (~145 bp) were gel-excised, eluted in 0.3 M NaCl overnight, and ethanol-precipitated. Raw reads were processed and analyzed using the CLASHub Analyzer module. Reagents, buffers, adapters, and spike-in sequences used in small RNA sequencing are listed in Supplementary Data 19.

### miRNA-seq data analysis and database construction

Paired-end FASTQ files were processed using a standardized small RNA-seq pipeline implemented in CLASHub. Adapter sequences were removed with Cutadapt, and paired-end reads were merged into full-length sequences using PEAR. CLASHub supports libraries with varying UMI configurations by allowing users to specify 5′ and 3′ UMI lengths in base pairs. When either UMI length is greater than zero, redundant sequences are collapsed using fastx_collapser to minimize ligation and amplification bias, and UMI sequences of the specified lengths are trimmed from both ends using Cutadapt prior to quantification. For example, AQ-seq libraries typically contain 4-nt UMIs at both the 5′ and 3′ ends. For standard small RNA-seq datasets generated using commonly used commercial kits such as Illumina TruSeq Small RNA or NEBNext Small RNA Library Prep, which do not include UMIs, users can set both UMI lengths to zero, and CLASHub automatically skips the PCR-duplicate collapsing and UMI-trimming steps, performing only adapter trimming and read merging. Single-end FASTQ libraries were processed using the same principles: adapter sequences were trimmed with Cutadapt, and when UMI lengths greater than zero are specified, duplicate collapsing and UMI trimming are performed; otherwise, these steps are skipped. For datasets provided directly as cleaned FASTA files, no preprocessing was required, and the sequences were used as-is for quantification.

After preprocessing, miRNA abundance was quantified using a custom CLASHub Python script that determines both total miRNA levels and isoform-specific levels by mapping the first 18 nucleotides of each read to a curated miRNA reference database.

To ensure reliable miRNA quantification, CLASHub Database prioritized datasets generated via AQ-seq, which minimizes ligation and amplification bias through UMI-based deduplication. Our primary sequencing depth threshold was >1 million aligned reads per sample. A small subset of samples falling slightly below this threshold was nonetheless retained because they constitute matched Control and *ZSWIM8*-knockout pairs from the same experiments, which are critical for comparative analysis of miRNA abundance changes (Supplementary Data 3).

For each sample, CLASHub automatically generates an HTML summary report containing adapter sequences, raw read counts, trimmed/merged/collapsed read statistics, aligned read numbers, and bar-chart visualizations summarizing the complete processing workflow and miRNA quantification results.

### RNA-seq data analysis and database construction

RNA-seq data were processed in CLASHub using a standardized pipeline integrating HISAT2, StringTie, and prepDE.py. Raw paired-end FASTQ files were first trimmed using Cutadapt to remove 5′ and 3′ adapters (minimum read length: 24 nt). Trimmed reads were aligned to the corresponding reference genome (hg38, mm39, BDGP6, or WBcel235) using HISAT2. Alignment parameters were dynamically adjusted based on the library type: the default mode was used for unstranded libraries, while the --rna-strandness RF flag was applied for stranded libraries. Resulting SAM files were sorted and indexed using SAMtools. For standard gene quantification, sorted BAM files were processed by StringTie (v2.2.1) in reference-guided mode (-eB) using Ensembl gene annotations (Release 115). The --rf flag was applied for stranded libraries to ensure correct strand assignment. This step generated per-gene abundance tables (TPM) and GTF files. Gene-level raw count matrices were subsequently generated using the prepDE.py3 script.

Differential expression analysis was performed using DESeq2 to identify significant changes between control and treatment groups. To distinguish transcriptional from post-transcriptional regulation and reduce false positives, we implemented the Exon-Intron Split Analysis (EISA) strategy^52^. For EISA, custom reference annotation files were generated for each species. To ensure the unambiguous assignment of intronic reads, genes overlapping with other transcripts on the same strand (for stranded libraries) or on either strand (for unstranded libraries) were excluded. Furthermore, exon coordinates were extended by 10 bp to mask exon-intron boundaries. Exonic and intronic abundances were then quantified separately by running StringTie and prepDE.py3 against these custom exon-specific and intron-specific GTF files. EISA categorization was performed based on the log_2_ fold-changes (Δ) of exons and introns. Genes were retained for analysis only if they met a stringent expression threshold (mean counts ≥24 for both exonic and intronic regions). Regulation types were classified into three categories: (i) Post-transcriptional, defined as genes with a significant divergence between exonic and intronic changes (∣ΔExon−ΔIntron∣ > 1); (ii) Transcriptional, defined as genes showing significant changes in both exons and introns (FDR < 0.05) with concordant magnitudes (∣ΔExon−ΔIntron∣ < 1); and (iii) Ambiguous, defined as genes not meeting the above criteria due to low statistical power or insufficient magnitude of difference.

CLASHub automatically parses logs from Cutadapt and HISAT2 to generate a Data Processing Summary HTML report for each job, visualizing total, trimmed, and aligned reads. To ensure high data quality, only samples with >10 million aligned reads and a genome alignment rate ≥80% were retained for downstream integration into the CLASHub Database module.

### miRNA targets identification from CLASH and TargetScan

CLASH-derived targets were identified by filtering for interactions mapping to Ensembl-annotated mRNAs, lncRNAs, and lincRNAs (excluding rRNAs, tRNAs, mitochondrial RNAs, snRNAs, snoRNAs, and pseudogenes) that satisfied high-confidence criteria. Specifically, retained interactions were required to possess either: (1) a canonical seed match (8mer, 7mer-m8, 7mer-A1, 6mer, or offset 6mer); or (2) for non-canonical interactions, significant Piranha peak support (*p* < 0.01) combined with structural features, including a continuous seed match ≥4 nt, extensive 3′ pairing ≥10 nt, and a geometric offset between −4 and +6 nt^66^. To be classified as conserved targets, human and mouse interaction must be detected in CLASH hybrid reads across at least two cell types, contain 8mer or 7mer seed matches, and have a phyloP conservation score >0. To determine the conservation score, we used UCSC Genome Browser phyloP: hg38.phyloP100way (human), mm39.phyloP35way (mouse), dm6.phyloP124way (*Drosophila*), and ce11.phyloP135way (*C. elegans*). For *C. elegans*, targets detected at any developmental stage (embryo, L3, or L4) are considered conserved, without requiring recurrence. For *Drosophila*, where only S2 cell data exist, no recurrence criterion is applied. In parallel, TargetScan-predicted targets are extracted from Summary_Counts.txt files (Human/Mouse Release 8; *Drosophila* Release 7.2; *C. elegans* Release 6.2), including conserved and non-conserved sites. For human, mouse, and *Drosophila*, Context++ scores were also extracted for each predicted target to facilitate downstream efficacy stratification.

### Cumulative fraction curve analysis pipeline

The cumulative fraction curve analysis module evaluates global miRNA-mediated repression by integrating user-uploaded differential expression data (containing GeneName, BaseMean, and log_2_FoldChange) with pre-compiled target databases. Prior to analysis, low-abundance transcripts can be filtered using a user-defined BaseMean threshold (default: 100). The module classifies targets into Standard groups (Conserved and All targets from CLASH and TargetScan) and, for human, mouse, and *Drosophila*, optional Stringent subsets. The stringent filtering selects the top 25% of predicted targets with the most favorable Context++ scores and further identifies high-confidence intersections between these top 25% predicted targets and experimentally validated CLASH targets.

To ensure statistical robustness and visualization clarity, target groups containing fewer than 10 genes (*N* < 10) after intersection with the user’s expression dataset are automatically excluded from the analysis. The cumulative distributions of log_2_FoldChange values for valid target groups are compared against a background of non-target genes. Statistical significance of the expression shifts is determined using two-sided Mann–Whitney *U* tests, and results are visualized as cumulative fraction plots alongside a downloadable summary table annotating the specific classification of each gene.

### Reporting summary

Further information on research design is available in the Nature Portfolio Reporting Summary linked to this article.

## Supplementary information

Supplementary Information

Reporting Summary

Transparent Peer Review file

## Source data

Source Data

## Data Availability

The sequencing data generated in this study have been deposited in the NCBI Sequence Read Archive (SRA) under accession PRJNA1166120. Information for all previously published datasets used in CLASHub—including CLASH, gene expression, and miRNA expression—is summarized in Supplementary Data 1–3, and additional details are available at. Sample input files for testing the CLASHub Analyzer modules are available at https://clashub.rc.ufl.edu/more_info/more_info.html (e.g., Fig. 5a: Sample CLASH FASTQ 1 and 2). The previously published CLASH datasets used in this study are available in the NCBI SRA and GEO database under accession codes PRJNA1093144, GSE303817, PRJNA896239, GSE198250, GSE164634, GSE124687, GSE101978, GSE73057, GSE73058, GSE56180, PRJNA328816^13,26,28,32–35,58,67,68^. For users who require batch-level customized analysis, CLASHub also provides a Bulk Download feature (https://clashub.rc.ufl.edu/more_info/bulk_download.html), enabling researchers to download comprehensive processed datasets for use with external analysis tools. All newly generated materials described in this study are available from the corresponding author upon reasonable request. Source data are provided with this paper.

## Code availability

CLASHub is a custom web application built with HTML, PHP, JavaScript, CSS, and sbatch scripts, hosted on the University of Florida’s HiPerGator infrastructure (PubApps). All source code for the CLASHub web application and Analyzer modules—including database construction pipelines, sbatch scripts, PHP modules, and downstream analysis scripts (CLASH processing, miRNA-seq quantification, RNA-seq with EISA analysis, and cumulative fraction curve analysis)—is publicly available at https://github.com/UF-Xie-Lab/CLASHub, and a permanent archive of the version used in this study is deposited on Zenodo at 10.5281/zenodo.19558292.
