# Supplementary material for: CLASHub is an integrated database and analytical platform for microRNA-target interactions
Source: Nat Commun. 2026 May 8;17:6204. doi: 10.1038/s41467-026-72902-x (PMC13369745; doi:10.1038/s41467-026-72902-x)
Supplement: Supplementary file 1 — Supplementary Information [file 41467_2026_72902_MOESM1_ESM.pdf]

Supplementary Figure 1

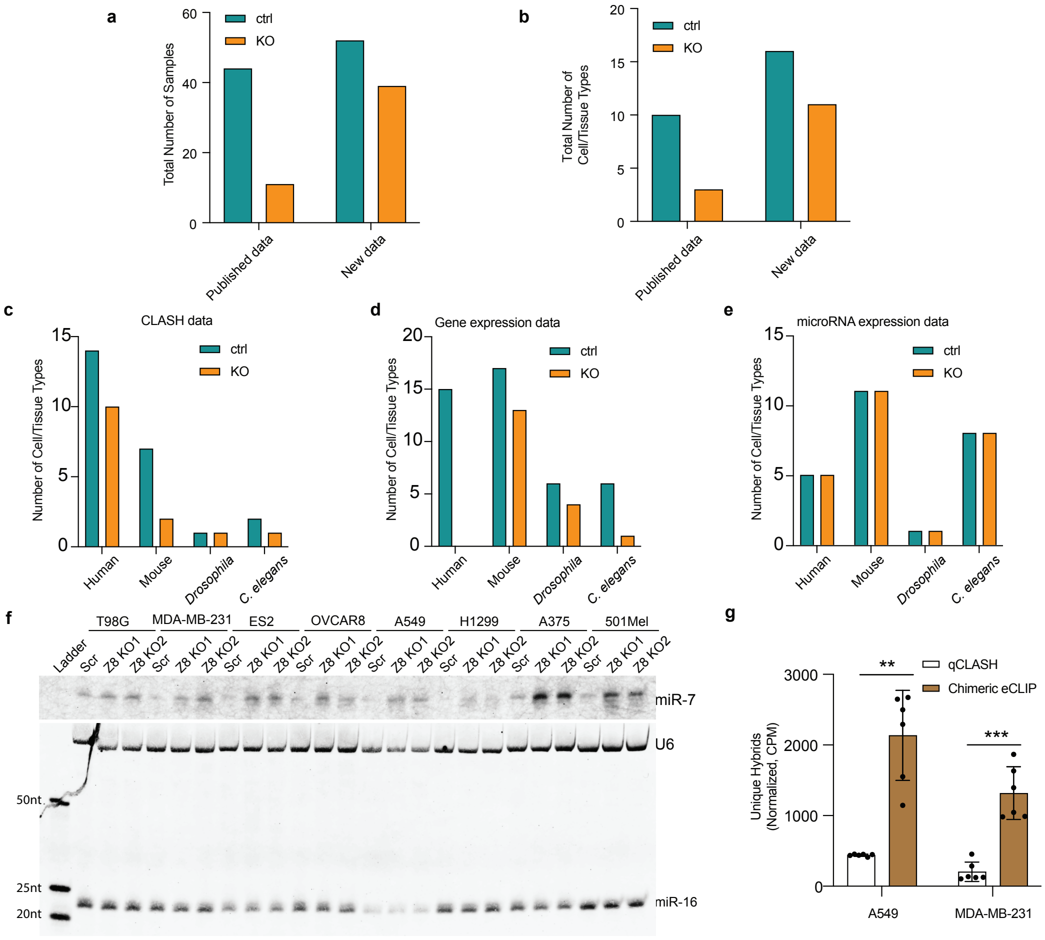

Supplementary Fig. 1 Overview of datasets in CLASHub and performance comparison of CLASH protocols.

a Bar plots summarizing the total number of samples and **b** total number of cell/tissue types derived from published and newly generated CLASH data. Bar plots showing the number of unique cell and tissue types included in the **c** CLASH, **d** gene expression and **e** miRNA expression data for human, mouse, *Drosophila*, and *C. elegans*, separated into control (Ctrl) and *ZSWIM8*-knockout (KO) conditions. **f** Functional validation of *ZSWIM8*-knockout cell lines generated in this study. Northern blot analysis of miR-7, U6 snRNA, and miR-16 abundance in control (Scr) and *ZSWIM8*-knockout (Z8 KO1, Z8 KO2) clones across the indicated cell lines. This experiment was performed once. **g** Performance comparison between qCLASH and chimeric eCLIP. To evaluate recovery efficiency, the unique hybrid yield was normalized to counts per million (CPM). The bead-based qCLASH protocol (white) was compared against the chimeric eCLIP protocol with SDS-PAGE and size selection on nitrocellulose membrane (brown) in MDA-MB-231 and A549 cells. Each data point represents one independent CLASH sequencing library; bars show mean ± SD ($n = 6$ biological replicates). Each cell line was analyzed independently by unpaired two-tailed Welch's t-test in GraphPad Prism. **$P < 0.01$, ***$P < 0.001$. Exact P values: A549, $P = 0.0013$; MDA-MB-231, $P = 0.0004$. Source data are provided as a Source Data file.

Supplementary Figure 2

CLASH: miR-7-5p in A549

Filter by Region: All Regions

Filter by Site Type: All Site Types

Download Table

<table>
<tr><th>miRNA Name</th><th>Pairing Pattern (UNAfold)</th><th>Gene Name</th><th>Gene ID</th><th>Conservation Score</th><th>Free Energy (kcal/mol)</th><th>Gene Type</th><th>Target Site Region</th><th>Genome Position</th><th></th><th>Chimeric eCLIP Control (Hybrid CPM)</th><th></th><th>Chimeric eCLIP KO (Hybrid CPM)</th><th>miRNA Exp (sgNT CPM)</th><th></th><th>Gene Exp (WT TPM)</th></tr>
<tr><td>miR-7-5p</td><td>miRNA: 5'UGGAAGACUA--GUGAUUUUGUUGUU
||||||| ||||||||||||||
target: 3'ACCUUCUGUAACCACUAAAACAACAA</td><td>OIP5-AS1</td><td>ENSG00000247556</td><td>6.53</td><td>-28.0</td><td>lncRNA</td><td></td><td>Chr15: 41300574-41300599</td><td></td><td>25.72</td><td></td><td>79.17</td><td>908.38</td><td></td><td>22.34</td></tr>
<tr><td>miR-7-5p</td><td>miRNA: 5'UGGAAGACUAGUGAUUUUGUUGUU
|||||||.|||.| |||.||
target: 3'ACCUUCUGGUCAUU--AACGACCU</td><td>LINC00632</td><td>ENSG00000203930</td><td>0.48</td><td>-23.7</td><td>lncRNA</td><td></td><td>chrX: 140783384-140783405</td><td></td><td>0</td><td></td><td>99.35</td><td>908.38</td><td></td><td>1.21</td></tr>
<tr><td>miR-7-5p</td><td>miRNA: 5'UGGAAGAC----UAGUG-AUUUUGUUGUU
||||||| .|||| |||||||||||
target: 3'CCCUUCUGUUUUGUCACAUAAAACAACAA</td><td>ARHGEF7</td><td>ENSG00000102606</td><td>-0.22</td><td>-22.9</td><td>mRNA</td><td>3' UTR</td><td>chr13: 111303922-111303950</td><td></td><td>0</td><td></td><td>8.38</td><td>908.38</td><td></td><td>18.74</td></tr>
<tr><td>miR-7-5p</td><td>miRNA: 5'UGGAAGACUAGUG-----AUUUUGUUGUU
|||||||||| |.|||.||||.
target: 3'GCCUUCUGAUCUUUUUUAUGAAAUAACAG</td><td>STRN3</td><td>ENSG00000196792</td><td>6.17</td><td>-22.9</td><td>mRNA</td><td>3' UTR</td><td>chr14: 30894050-30894078</td><td></td><td>0</td><td></td><td>0.93</td><td>908.38</td><td></td><td>13.61</td></tr>
<tr><td>miR-7-5p</td><td>miRNA: 5'UGGAAGACUA-----GUGAUUUUGUUGUU
||||||| ||||. ||||||
target: 3'ACCUUCUGUUCCACCCACUGUAACAACCG</td><td>CRTC2</td><td>ENSG00000160741</td><td>4.64</td><td>-21.7</td><td>mRNA</td><td>3' UTR/ CDS</td><td>chr1: 153951268-153951296</td><td></td><td>0</td><td></td><td>1.69</td><td>908.38</td><td></td><td>27.21</td></tr>
<tr><td>miR-7-5p</td><td>miRNA: 5' UGGAAGACUAGUGAUUUUGUUGUU
|||||| .|||.|. |||||.
target: 3' GCCUUCUUGUCAUUGU--CAACAG</td><td>TRIM8</td><td>ENSG00000171206</td><td>6.76</td><td>-21.7</td><td>mRNA</td><td>3' UTR/ CDS</td><td>chr10: 102657109-102657130</td><td>…</td><td>0</td><td>…</td><td>0.76</td><td>908.38</td><td>…</td><td>36.85</td></tr>
<tr><td>miR-7-5p</td><td>miRNA: 5' UGGAAGACUA--GUGAUUUUGUUGUU
||||||| ||| ||.|||||
target: 3' ACCUUCUGUUUACACGAAGACAACCG</td><td>RYK</td><td>ENSG00000163785</td><td>2.15</td><td>-21.5</td><td>mRNA</td><td>3' UTR</td><td>chr3: 134158040-134158065</td><td></td><td>0</td><td></td><td>8.33</td><td>908.38</td><td></td><td>25.88</td></tr>
<tr><td>miR-7-5p</td><td>miRNA: 5' UGGAAGACUAGUGAUUUUGUUGUU
|||||| |||||.||||.|
target: 3' AACUUCUG-UCACUGAAACGAACC</td><td>SOX7</td><td>ENSG00000171056</td><td>0.03</td><td>-21.4</td><td>mRNA</td><td>3' UTR</td><td>chr8: 10724529-10724551</td><td></td><td>0</td><td></td><td>0</td><td>908.38</td><td></td><td>1.2</td></tr>
<tr><td>miR-7-5p</td><td>miRNA: 5' UGGAAGACUAGUGAUUUUGUUGUU
|||||||| ||| .||.|.|.
target: 3' ACCUUCUGAG-ACUU-GACGAUAG</td><td>CWC22</td><td>ENSG00000163510</td><td>3.1</td><td>-21.4</td><td>mRNA</td><td>CDS</td><td>chr2: 179950560-179950581</td><td></td><td>1.72</td><td></td><td>0</td><td>908.38</td><td></td><td>10.24</td></tr>
<tr><td>miR-7-5p</td><td>miRNA: 5' UGG--AAGACUAG--UGAUUUUGUUGUU
|| |||||. ||||||||||||.
target: 3' GCCGGUUCUGGAGUAACUAAAACAACAG</td><td>CYP24A1</td><td>ENSG00000019186</td><td>3.66</td><td>-21.2</td><td>mRNA</td><td>CDS</td><td>chr20: 54169679-54171594</td><td></td><td>0</td><td></td><td>0.76</td><td>908.38</td><td></td><td>455.89</td></tr>
</table>

First Previous 1 2 3 4 5 ... Next Last

Supplementary Fig. 2 Column-based sorting of the CLASH table.

Example showing miR-7-5p hybrids in A549 cells sorted by free energy (kcal/mol) in ascending order. The red arrow indicates the sorted column header. Sorting by free energy ranks hybrids by predicted duplex stability, with the most thermodynamically stable interactions displayed first. Created in BioRender. Traugot, C. (2026) https://BioRender.com/tm1ake1.

Supplementary Figure 3

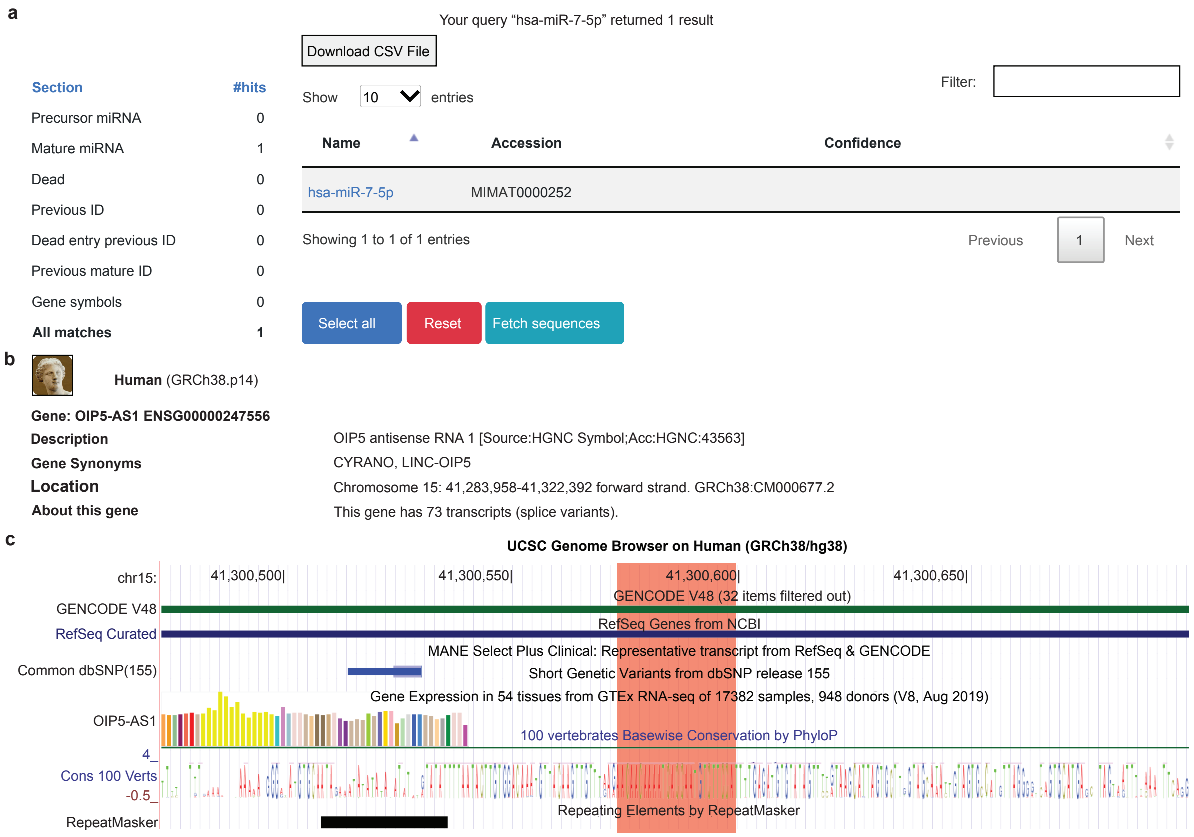

Supplementary Fig. 3 Example external resources linked from the CLASH results table.

a miRBase page opened by clicking on the miRNA name (hsa-miR-7-5p). **b** Ensembl gene summary page opened by clicking on the gene ID (*OIP5-AS1*). **c** UCSC Genome Browser view showing the genomic locus corresponding to the hybrid site.

Supplementary Figure 4

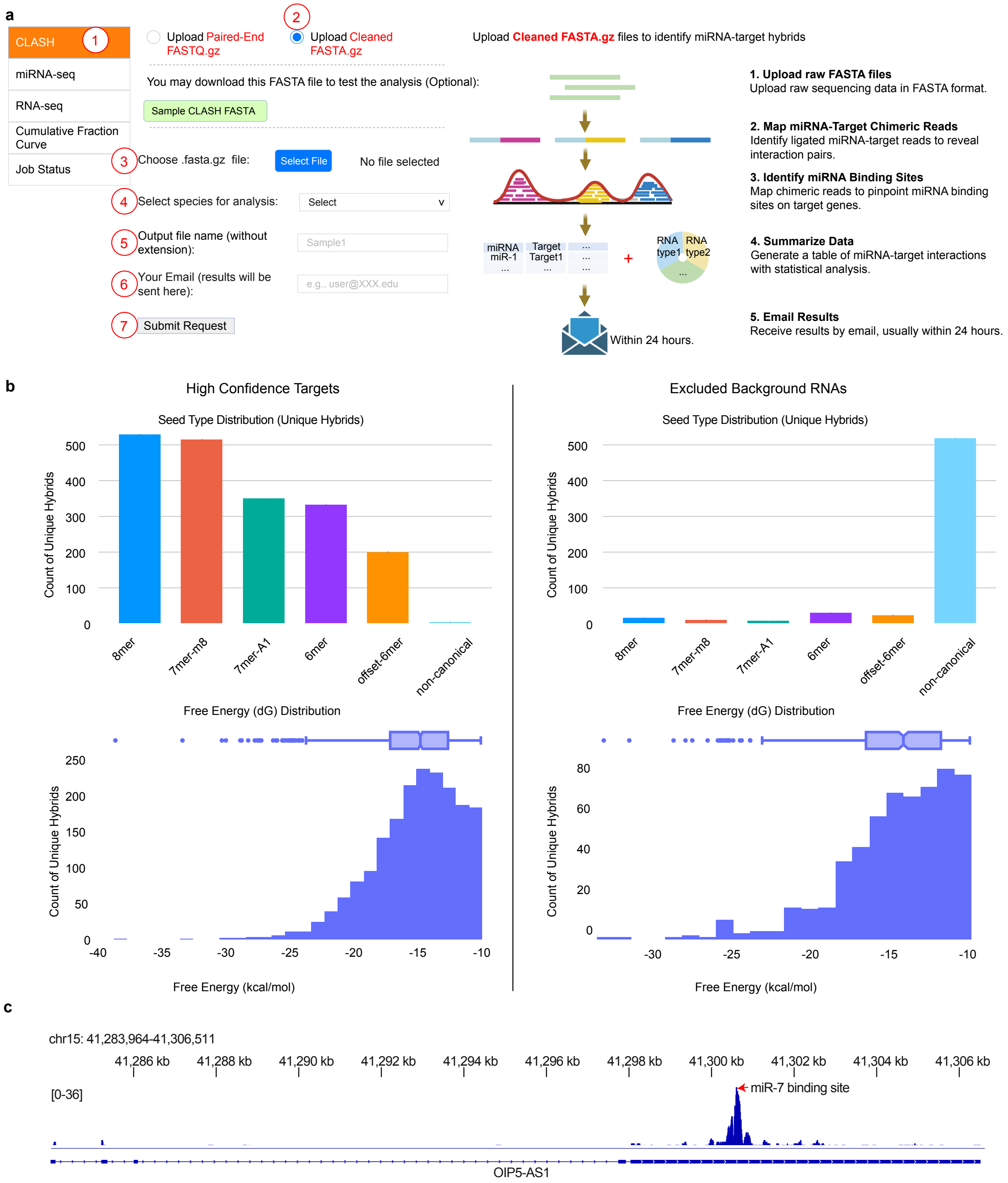

Supplementary Fig. 4 CLASH analysis workflow for pre-processed FASTA inputs and quality assessment reports.

a Interface for submitting CLASH analysis with cleaned FASTA files. Users (1) select the CLASH module, (2) choose "cleaned FASTA.gz" as input file type, (3) select a local FASTA file for upload, (4) select species, (5) specify output filename, (6) enter email address, and (7) submit the request. The backend bioinformatic pipeline to process cleaned FASTA data is shown on the right. Created in BioRender. Traugot, C. (2026) https://BioRender.com/vndweld. **b** Example of the comparative quality assessment report generated by the CLASH Analyzer module. The charts demonstrate the characteristics of unique miRNA-target hybrids by distinguishing High Confidence Targets from Excluded Background RNAs (e.g., rRNAs, tRNAs, mitochondrial RNAs, and pseudogenes). **c** Genome-level visualization of mapped non-hybrid reads in CLASH data. For each submitted FASTA file, CLASHub generates a bigWig (bw) file for inspection in IGV. Shown is an example of miR-7 binding to *OIP5-AS1* (*Cyrano*) in the human hg38 genome.

Supplementary Figure 5

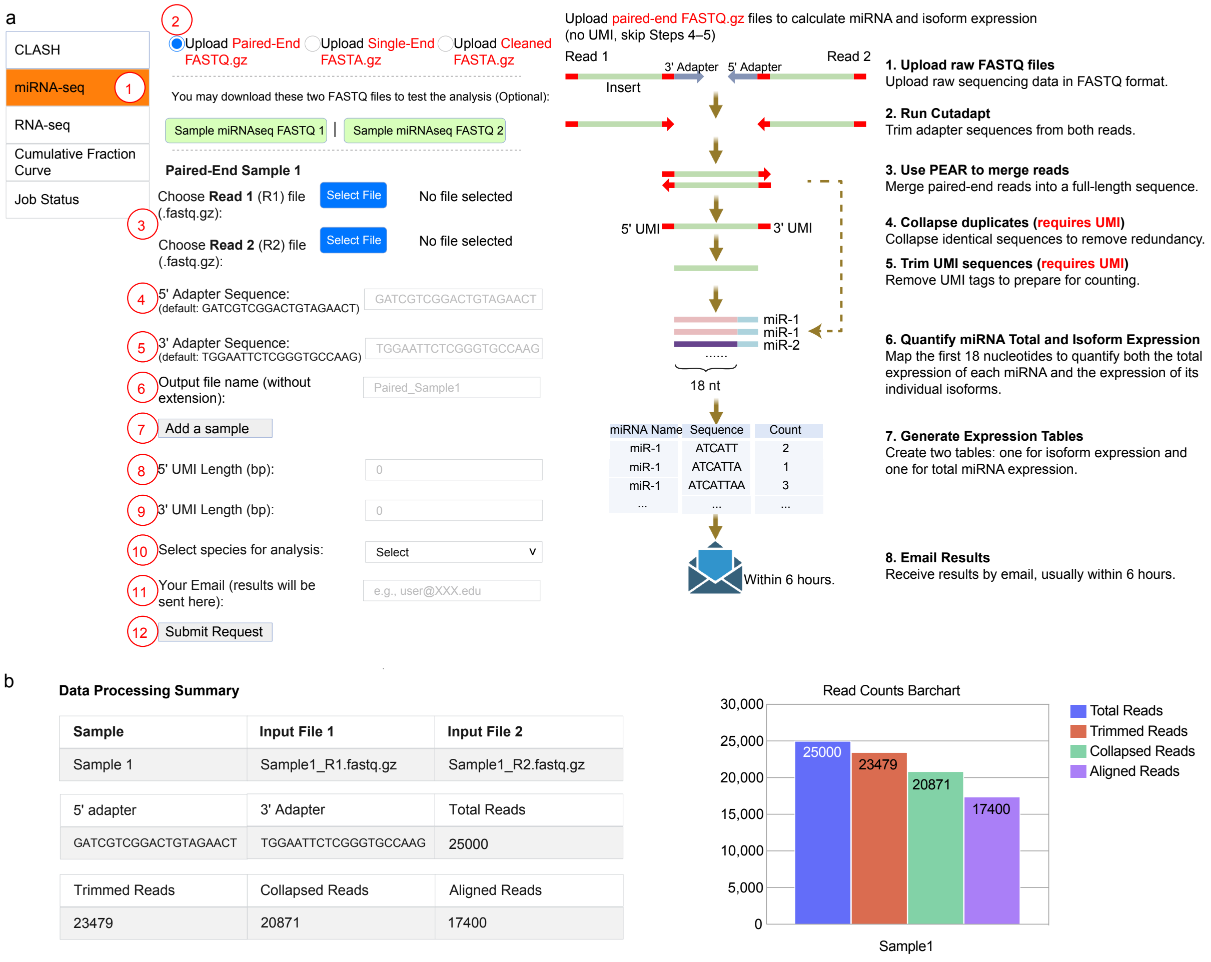

Supplementary Fig. 5 miRNA-seq analysis interface and processing overview.

a Interface for miRNA-seq analysis. Users (1) select the module, (2) choose input file type, (3) select paired-end FASTQ files to upload, (4-5) enter adapter sequences, (6) specify output filename, (7) can optionally add additional samples, (8-9) define the length of 5′ and 3′ Unique Molecular Identifiers (UMIs), (10) select species, (11) provide email address, and (12) submit the request. The backend bioinformatic pipeline to process paired-end FASTQ data is shown on the right. Created in BioRender. Traugot, C. (2026) https://BioRender.com/zntatdw. **b** Output summary and a bar chart displaying statistics of the processed reads.

Supplementary Figure 6

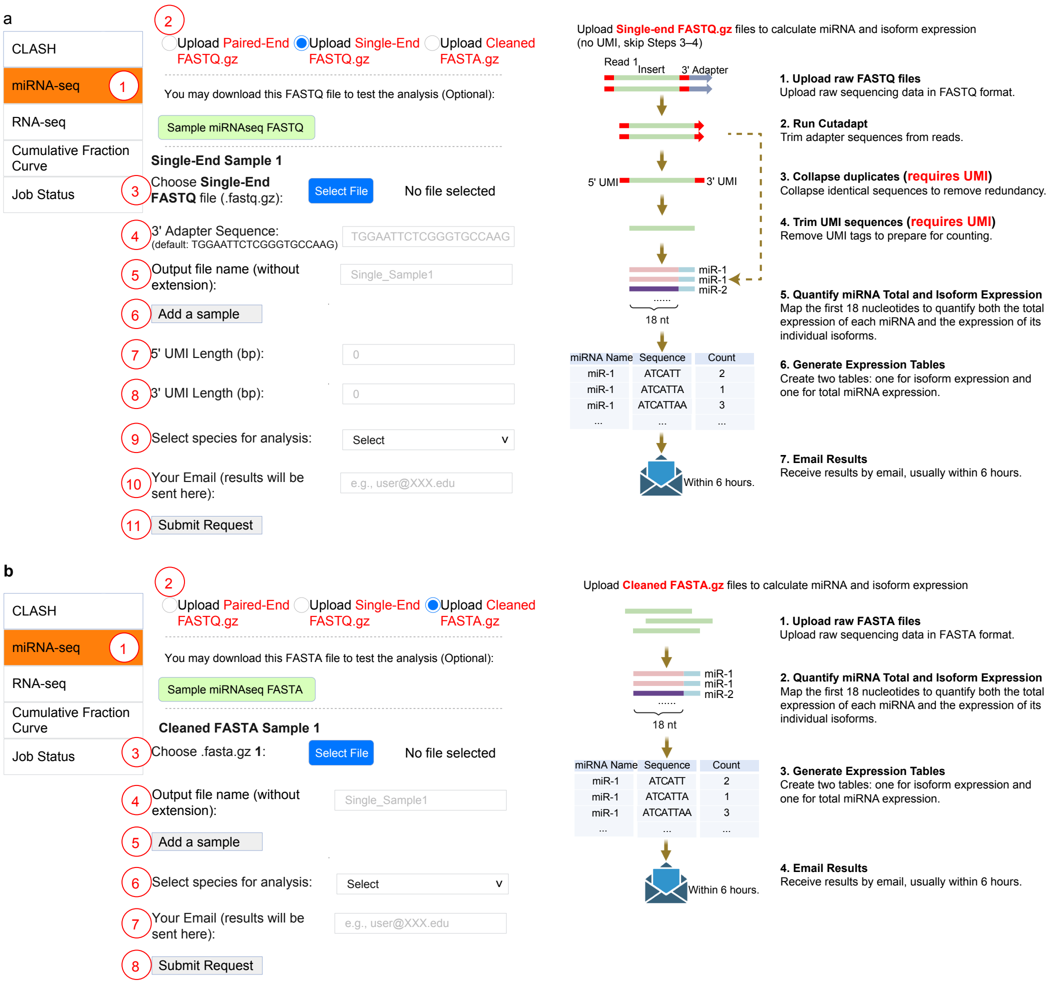

Supplementary Fig. 6 miRNA-seq analysis workflows for single-end FASTQ and cleaned FASTA inputs.

a Interface for processing single-end FASTQ files. Users (1) select the miRNA-seq module, (2) choose the "Single-End FASTQ" input, (3) choose a local file to upload, (4) enter the adapter sequence, (5) specify the output filename, (6) can optionally add additional samples, (7-8) define the length of 5′ and 3′ Unique Molecular Identifiers (UMIs), (9) select the species, (10) provide an email address, and (11) submit the request. Created in BioRender. Traugot, C. (2026) https://BioRender.com/vtbyqaw. **b** Interface for processing cleaned FASTA files. Users (1) select the miRNA-seq module, (2) choose the "Cleaned FASTA" input, (3) choose a local file to upload, (4) specify the output filename, (5) can optionally add additional samples, (6) select the species, (7) provide an email address, and (8) submit. Created in BioRender. Traugot, C. (2026) https://BioRender.com/80fy16k.

Supplementary Figure 7

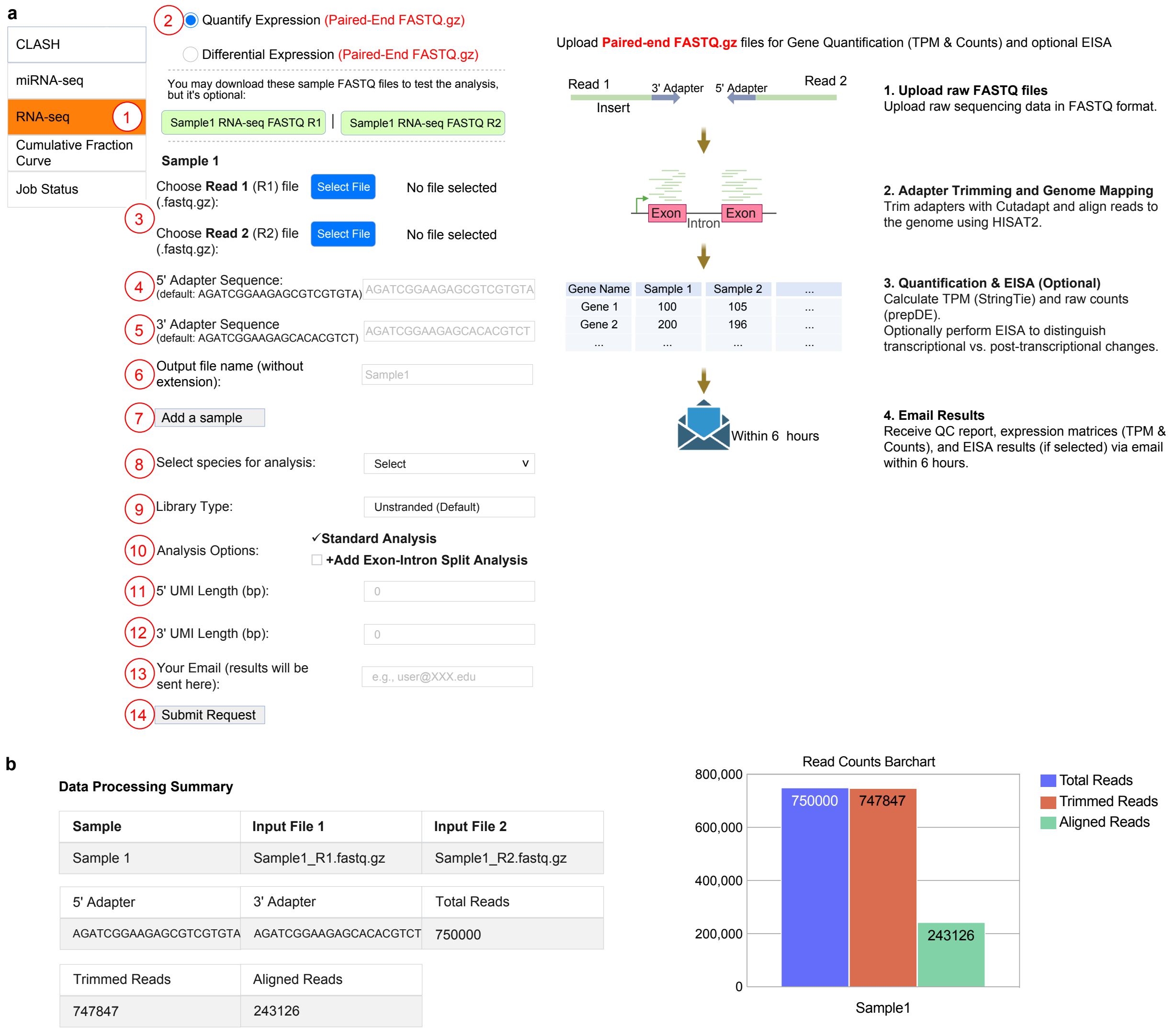

Supplementary Fig. 7 RNA-seq analysis module interface and processing summary.

a Interface for RNA-seq gene expression quantification. Users (1) select the RNA-seq module, (2) choose paired-end FASTQ input, (3) upload Read 1 and Read 2 files, (4-5) enter adapter sequences, (6) specify output filename, (7) add more samples if needed, (8) select species, (9) specify the library type (unstranded or stranded), (10) configure analysis options (standard analysis is performed by default, with an option to add Exon-Intron Split Analysis), (11-12) define the length of 5′ and 3′ UMIs, (13) provide an email address, and (14) submit the request. The backend bioinformatic pipeline to process paired-end FASTQ data is shown on the right. Created in BioRender. Traugot, C. (2026) https://BioRender.com/76j0l5h. **b** Example processing output showing adapter sequences, read counts across each step, and a bar chart summarizing total, trimmed, and aligned reads.

Supplementary Figure 8

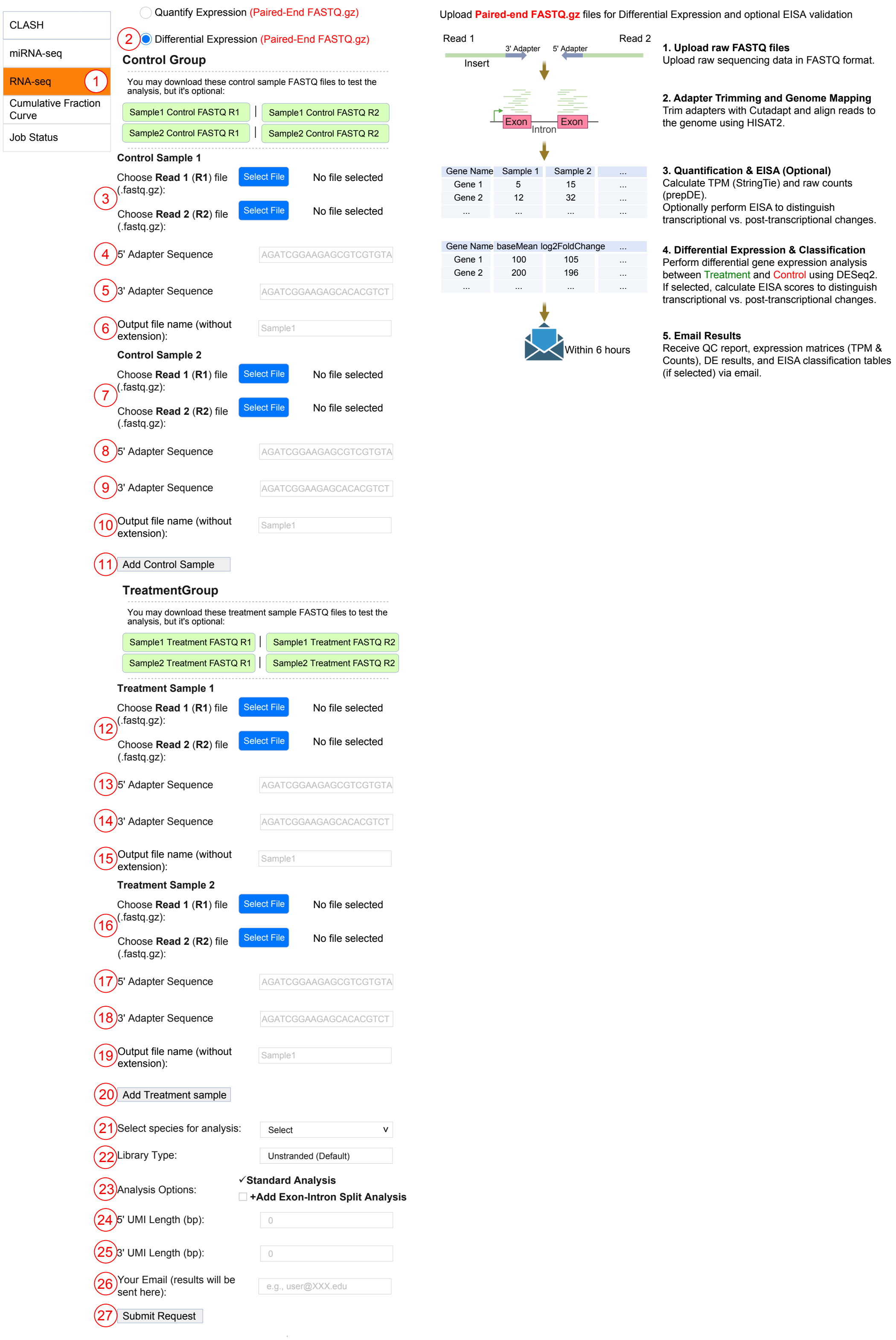

Supplementary Fig. 8 RNA-seq differential expression analysis module interface and workflow.

Interface for differential gene expression analysis. Users (1) select the RNA-seq module, (2) choose the differential expression option, (3-10) upload at least two control group paired-end FASTQ files with the option to add additional samples (11), (12–20) upload two or more treatment group files, specify adapter sequences and output filenames, (21) select species, (22) specify the library type (unstranded or stranded), (23) configure analysis options (standard differential expression is performed by default, with an option to add Exon-Intron Split Analysis), (24-25) define the length of 5′ and 3′ UMIs, (26) provide an email address, and (27) submit the request. The backend pipeline that performs adapter trimming, genome mapping, count generation, and differential expression analysis is shown on the right. Created in BioRender. Traugot, C. (2026) https://BioRender.com/7rwujql.

Supplementary Figure 9

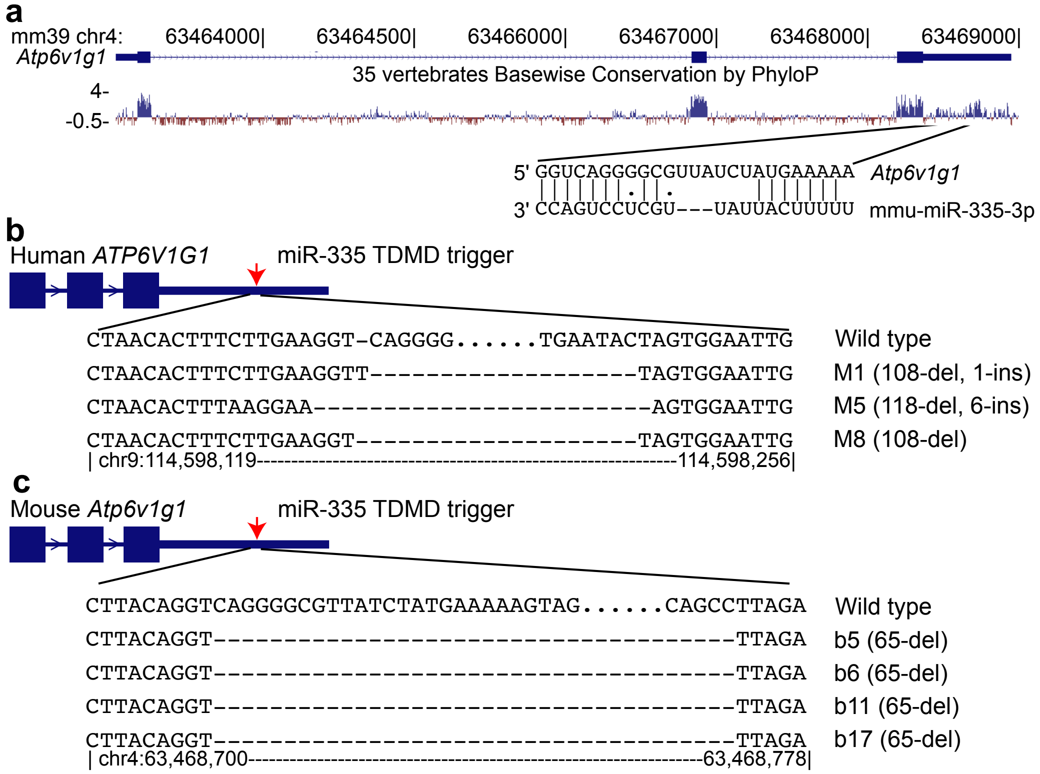

Supplementary Fig. 9 Conservation and genome editing of the putative miR-335-3p trigger within *ATP6V1G1*.

a UCSC Genome Browser view of the *Atp6v1g1* locus on mouse chromosome 4 (mm39), showing phyloP conservation scores across 35 vertebrates. The miR-335-3p binding site displays contiguous pairing at the seed and 3′ end regions. **b** Schematic of the human *ATP6V1G1* transcript with the miR-335-3p TDMD trigger indicated (red arrow). Sanger sequencing results of CRISPR/Cas9-edited MDA-MB-231 clones are shown: M1 (108 bp deletion (del) +1 bp insertion (ins)), M5 (118 bp deletion +6 bp insertion), and M8 (108 bp deletion). **c** Schematic of the mouse *Atp6v1g1* transcript with the miR-335-3p TDMD trigger (red arrow). Sanger sequencing confirms four MEF clones (b5, b6, b11, b17) carrying identical 65 bp deletions (del).

Supplementary Table 1

<table>
<tr><th>Pairing Pattern (UNAfold)</th><th>Gene Name
miRNA Name</th><th>Conservation Score</th><th>Free Energy (kcal/mol)</th><th colspan="2">Cell Lines/Tissues Detected</th><th>Mean Hybrid Abundance in control cell</th><th>Mean Hybrid Abundance in ZSWIM8 Knockout</th></tr>
<tr><td>5'GGUCAGGGGCUUUAUCUAUGAAAAA
   |||||||.|| || |||||||
3'CCAGUCCUCG--UUA-UUACUUUUU</td><td>ATP6V1G1
hsa-miR-335-3p</td><td>2.84</td><td>-23.4</td><td rowspan="2">A549/ D425/ ES2/ H1299/ HCT116/ MB002/ OVCAR8/ T98G/ mouse kidney/ MDA-MB-231</td><td rowspan="2">10</td><td rowspan="2">27.52</td><td rowspan="2">211.44</td></tr>
<tr><td>5'GGUCAGGGGCGUUAUCUAUGAAAAA
   |||||||.||. |||||||
3'CCAGUCCUCGU---UAUUACUUUUU</td><td>Atp6v1g1
mmu-miR-335-3p</td><td>2.38</td><td>-24.4</td></tr>
<tr><td>5'GGUCAGGAGCAA-AUUGAAAAA
   ||||||||||| ||||||
3'CCAGUCCUCGUUAUUACUUUUU</td><td>LPAR4
hsa-miR-335-3p</td><td>0.86</td><td>-25.8</td><td rowspan="2">MB002/ mouse striatal cell</td><td rowspan="2">2</td><td rowspan="2">0</td><td rowspan="2">15.50</td></tr>
<tr><td>5'GGUCGGGAGCA-UAUUGAAAAA
   ||||.|||||| ||||||
3'CCAGUCCUCGUUAUUACUUUUU</td><td>Lpar4
mmu-miR-335-3p</td><td>0.33</td><td>-24.9</td></tr>
<tr><td>5'GUUCAGGGGCCAUGGAAAAUGAAAAA
     |||||.|| .| ||||||||
3'CCAGUCCUCG----UUAUUACUUUUU</td><td>MRM2
hsa-miR-335-3p</td><td>-0.06</td><td>-18.4</td><td>A549/ HCT116/ MB002/ Colorectal tissue</td><td>4</td><td>23.86</td><td>0.16</td></tr>
<tr><td>5'GGUCAGGAGGGAGACACAACAUAUUUUUGAUCAUGAAAAA
  ||||||||| ..|| |||||||
3'CCAGUCCUC------------------GUUAUUACUUUUU</td><td>ARHGAP18
hsa-miR-335-3p</td><td>-0.28</td><td>-18.2</td><td>HCT116</td><td>1</td><td>0</td><td>0.66</td></tr>
<tr><td>5'UCUCAGGAGCUGUCACAGGAUUUAUUACUGUGACUUGAAAAA
    |||||||| .|.| ||||||
3'CCAGUCCUCG------------------UUAUU--ACUUUUU</td><td>Atg5
mmu-miR-335-3p</td><td>0.98</td><td>-15.2</td><td>Heart</td><td>1</td><td>11.94</td><td>N/A</td></tr>
</table>

Note: Italicized text indicates gene names.

Supplementary Table 1. Candidate miR-335-3p TDMD Hybrids Detected in Human and Mouse CLASH Data.

Pairing patterns were visualized using UNAFold; "|" denotes Watson-Crick base pairs and "." denotes G·U wobble pairs. Dashes represent unpaired nucleotides; red text highlights the miRNA seed region. Cell Lines/Tissues Detected lists all unique human and mouse cell lines or tissues in which each chimera was identified across the combined CLASH datasets analyzed in this study, with the total count shown (multiple samples from the same cell line or tissue are counted once). Mean Hybrid Abundance reports the average chimeric read abundance (CPM) of each miRNA–target pair, averaged across all cell lines/tissues in which the hybrid was detected, under control (non-targeting sgRNA) and ZSWIM8-knockout conditions. N/A indicates no available ZSWIM8-knockout dataset.

Supplementary Table 2

<table>
<tr><th>Pairing Pattern (UNAfold)</th><th>Gene Name
miRNA Name</th><th>Conservation Score</th><th>Free Energy (kcal/mol)</th><th>Detected Cell lines (n=15)</th><th>Mean Abundance in HEK293T (CPM)</th></tr>
<tr><td>5'CAGCUUGCAC----AGCACCUUA
.||||| |||||||
3'GAUAGACGUGAUCUACGUGGAAU</td><td>ATXN1L
hsa-miR-18a-5p</td><td>3.17</td><td>-15.7</td><td>5</td><td>71.99</td></tr>
<tr><td>5'AUUCAUG-AAUAAAUGCACCUUA
|| || |||||||||
3'GAUAGACGUGAUCUACGUGGAAU</td><td>FAM3C
hsa-miR-18a-5p</td><td>1.42</td><td>-15.6</td><td>11</td><td>218.3</td></tr>
<tr><td>5'AGACCUGC-CU-UAUGCACCUUA
|||| || |||||||||
3'GAUAGACGUGAUCUACGUGGAAU</td><td>NEDD4
hsa-miR-18a-5p</td><td>2.02</td><td>-19.3</td><td>12</td><td>176.25</td></tr>
<tr><td>5'UGAAUUUUACU---UGCACCUUA
.||| ||||||||
3'GAUAGACGUGAUCUACGUGGAAU</td><td>PDE4D
hsa-miR-18a-5p</td><td>3.83</td><td>-14</td><td>4</td><td>29.98</td></tr>
<tr><td>5'GAUUCUGC-----CUGCACCUUA
||||| ||||||||
3'GAUAGACGUGAUCUACGUGGAAU</td><td>RNF4
hsa-miR-18a-5p</td><td>0.05</td><td>-17.1</td><td>12</td><td>473.18</td></tr>
<tr><td>5'UUUCCCUUCAAAAUAGCACCUUA
|||||||
3'GAUAGACGUGAUCUACGUGGAAU</td><td>ZBTB4
hsa-miR-18a-5p</td><td>4.06</td><td>-12.2</td><td>5</td><td>71.72</td></tr>
<tr><td>5'CCUUGGGCAUAAGCUAAGCACCUUA
|||. || |||||||
3'GAUAGACGUGAUC--UACGUGGAAU</td><td>ZNF367
hsa-miR-18a-5p</td><td>5.74</td><td>-15.9</td><td>9</td><td>631.91</td></tr>
</table>

Note: Italicized text indicates gene names.

Supplementary Table 2. Seven miR-18a-5p candidate targets selected for experimental validation in HEK293T cells.

Pairing patterns were visualized using UNAFold, with "|" indicating Watson-Crick base pairs and "." indicating G·U wobble pairs. Dashes within sequences represent unpaired nucleotides (bulges/gaps); red text highlights the miRNA seed region. Detected Cell Lines (n=15) indicates the number of human cell lines, out of 15 CLASH datasets analyzed, in which the miRNA–target chimera was identified. Mean Abundance in HEK293T represents the average chimeric read abundance (CPM) of the miRNA–target pair across HEK293T replicates.
