# Supplementary material for: CLASHub is an integrated database and analytical platform for microRNA-target interactions
Source: Nat Commun. 2026 May 8;17:6204. doi: 10.1038/s41467-026-72902-x (PMC13369745; doi:10.1038/s41467-026-72902-x)
Supplement: Supplementary file 2 — Reporting Summary [file 41467_2026_72902_MOESM2_ESM.pdf]

nature portfolio

Corresponding author(s): *Double-anonymous peer review submissions: write DAPR and your manuscript number here instead of author names.*

Last updated by author(s): *YYYY-MM-DD*

Reporting Summary

Nature Portfolio wishes to improve the reproducibility of the work that we publish. This form provides structure for consistency and transparency in reporting. For further information on Nature Portfolio policies, see our Editorial Policies and the Editorial Policy Checklist.

Statistics

For all statistical analyses, confirm that the following items are present in the figure legend, table legend, main text, or Methods section.

n/a	Confirmed	
☐	☒	The exact sample size (*n*) for each experimental group/condition, given as a discrete number and unit of measurement
☐	☒	A statement on whether measurements were taken from distinct samples or whether the same sample was measured repeatedly
☐	☒	The statistical test(s) used AND whether they are one- or two-sided<br>*Only common tests should be described solely by name; describe more complex techniques in the Methods section.*
☒	☐	A description of all covariates tested
☐	☒	A description of any assumptions or corrections, such as tests of normality and adjustment for multiple comparisons
☐	☒	A full description of the statistical parameters including central tendency (e.g. means) or other basic estimates (e.g. regression coefficient) AND variation (e.g. standard deviation) or associated estimates of uncertainty (e.g. confidence intervals)
☐	☒	For null hypothesis testing, the test statistic (e.g. *F*, *t*, *r*) with confidence intervals, effect sizes, degrees of freedom and *P* value noted<br>*Give P values as exact values whenever suitable.*
☒	☐	For Bayesian analysis, information on the choice of priors and Markov chain Monte Carlo settings
☒	☐	For hierarchical and complex designs, identification of the appropriate level for tests and full reporting of outcomes
☒	☐	Estimates of effect sizes (e.g. Cohen's *d*, Pearson's *r*), indicating how they were calculated

Our web collection on statistics for biologists contains articles on many of the points above.

Software and code

Policy information about availability of computer code

Data collection	No custom software was used for data collection. Sequencing data were collected on Illumina NovaSeq platforms using standard Illumina software.
Data analysis	Cutadapt v2.10, PEAR v0.9.6, fastx_collapser v0.0.14, Hyb, Bowtie2 v2.5.3, UNAfold v3.8, HISAT2, SAMtools v1.21, BEDTools v2.31.1, bedGraphToBigWig v2.10, Piranha v1.2.1, StringTie v2.2.1, prepDE.py3, DESeq2, GraphPad Prism (for statistical testing). Custom scripts (CLASHub.py and the full CLASHub Analyzer pipeline) are publicly available at https://github.com/UF-Xie-Lab/CLASHub and archived at https://doi.org/10.5281/zenodo.19558292. Reference databases: Ensembl Release 115, miRBase Release 22.1, TargetScan (Human/Mouse Release 8, Drosophila Release 7.2, C. elegans Release 6.2), UCSC phyloP (hg38.phyloP100way, mm39.phyloP35way, dm6.phyloP124way, ce11.phyloP135way).

For manuscripts utilizing custom algorithms or software that are central to the research but not yet described in published literature, software must be made available to editors and reviewers. We strongly encourage code deposition in a community repository (e.g. GitHub). See the Nature Portfolio guidelines for submitting code & software for further information.

Data

Policy information about availability of data

All manuscripts must include a data availability statement. This statement should provide the following information, where applicable:

- Accession codes, unique identifiers, or web links for publicly available datasets
- A description of any restrictions on data availability
- For clinical datasets or third party data, please ensure that the statement adheres to our policy

The sequencing data generated in this study have been deposited in the NCBI Sequence Read Archive (SRA) under accession PRJNA1166120. Previously published datasets used in this study are available under accession codes PRJNA1093144, GSE303817, PRJNA896239, GSE198250, GSE164634, GSE124687, GSE101978, GSE73057, GSE73058, GSE56180, and PRJNA328816. Information for all integrated datasets is summarized in Supplementary Data 1–3 and at https://clashub.rc.ufl.edu/more_info.html. Source data are provided with this paper. The CLASHub platform is publicly accessible at https://clashub.rc.ufl.edu/.

Research involving human participants, their data, or biological material

Policy information about studies with human participants or human data. See also policy information about sex, gender (identity/presentation), and sexual orientation and race, ethnicity and racism.

Reporting on sex and gender	two human colorectal samples were used; sex and gender information were not collected.
Reporting on race, ethnicity, or other socially relevant groupings	Race, ethnicity, and other socially relevant variables were not collected for this sample.
Population characteristics	No additional population characteristics were collected beyond those reported.
Recruitment	The human colorectal samples were obtained from a single donor under an approved IRB protocol (IRB201500756). No additional recruitment was performed.
Ethics oversight	The human colorectal tissue was approved under University of Florida IRB protocol IRB201500756.

Note that full information on the approval of the study protocol must also be provided in the manuscript.

Field-specific reporting

Please select the one below that is the best fit for your research. If you are not sure, read the appropriate sections before making your selection.

☒ Life sciences ☐ Behavioural & social sciences ☐ Ecological, evolutionary & environmental sciences

For a reference copy of the document with all sections, see nature.com/documents/nr-reporting-summary-flat.pdf

Life sciences study design

All studies must disclose on these points even when the disclosure is negative.

Sample size	Sample sizes were chosen based on standard practice in the field of miRNA and CLASH research. RT-qPCR experiments used n=3 independent biological replicates; dual-luciferase reporter assays used n=4 independent biological replicates. CLASH and miRNA-seq experiments used n=2–6 biological replicates per condition as detailed in figure legends and Supplementary Data 1–3. No statistical methods were used to predetermine sample size.
Data exclusions	For database integration, CLASH datasets were required to exceed 5,000 unique miRNA–target hybrids per sample with ≥2 biological replicates per cell type/tissue. miRNA-seq datasets required >1 million aligned reads (with limited exceptions for matched control/knockout pairs as described in Methods). RNA-seq datasets required >10 million aligned reads and ≥80% genome alignment rate. These exclusion criteria were pre-established.
Replication	All experimental findings were reproduced across multiple independent biological replicates (n=3 for RT-qPCR, n=4 for luciferase assays, n=2–6 for sequencing experiments). All replication attempts were successful. Three independent homozygous ATP6V1G1 trigger knockout clones in MDA-MB-231 and four independent clones in MEF cells confirmed reproducibility.
Randomization	not applicable
Blinding	not applicable

Reporting for specific materials, systems and methods

We require information from authors about some types of materials, experimental systems and methods used in many studies. Here, indicate whether each material, system or method listed is relevant to your study. If you are not sure if a list item applies to your research, read the appropriate section before selecting a response.

Materials & experimental systems

n/a	Involved in the study
☐	☒ Antibodies
☐	☒ Eukaryotic cell lines
☒	☐ Palaeontology and archaeology
☐	☒ Animals and other organisms
☒	☐ Clinical data
☒	☐ Dual use research of concern
☒	☐ Plants

Methods

n/a	Involved in the study
☒	☐ ChIP-seq
☒	☐ Flow cytometry
☒	☐ MRI-based neuroimaging

Antibodies

Antibodies used	anti-AGO2 clone 4F9 (produced in-house by the UF ICBR Monoclonal Antibody Core; ~80 µg per IP) for human and mouse CLASH; anti-Drosophila Ago1 (Abcam, ab5070, polyclonal; 20 µg per IP) for Drosophila CLASH.
Validation	The specificity of the anti-AGO2 (4F9 serum) antibody was validated from our lab before (Li et al., Genes Dev 2021). anti-Drosophila Ago1 antibody (Abcam, ab5070) from https://www.abcam.com/en-us/products/primary-antibodies/ago1-antibody-ab5070.

Eukaryotic cell lines

Policy information about cell lines and Sex and Gender in Research

Cell line source(s)	Human cancer cell lines (A549, D425, ES2, HCT116, HEK293T, HepG2, H1299, MB002, MDA-MB-231, OVCAR8, T98G, U87MG, 501Mel) were obtained from in-house stocks or collaborating laboratories. Mouse embryonic fibroblasts (MEFs) were derived in-house. A human colorectal tissue sample was obtained under IRB approval (IRB201500756). Mouse heart and kidney tissues were collected from a collaborating laboratory.
Authentication	Not independently authenticated
Mycoplasma contamination	Not tested
Commonly misidentified lines (See ICLAC register)	None

Animals and other research organisms

Policy information about studies involving animals; ARRIVE guidelines recommended for reporting animal research, and Sex and Gender in Research

Laboratory animals	Heart and kidney tissues were collected from C57BL/6 mice.
Wild animals	n/a
Reporting on sex	n/a
Field-collected samples	n/a
Ethics oversight	n/a

Note that full information on the approval of the study protocol must also be provided in the manuscript.

Plants

Seed stocks	Not applicable
Novel plant genotypes	Not applicable
Authentication	Not applicable
