# Supplementary material for: CLASHub is an integrated database and analytical platform for microRNA-target interactions
Source: Nat Commun. 2026 May 8;17:6204. doi: 10.1038/s41467-026-72902-x (PMC13369745; doi:10.1038/s41467-026-72902-x)
Supplement: Supplementary file 3 — Transparent Peer Review file [file 41467_2026_72902_MOESM3_ESM.pdf]

nature portfolio

Peer Review File

CLASHub is an integrated database and analytical platform for microRNA-target interactions

Corresponding Author: Dr Mingyi Xie

This file contains all reviewer reports in order by version, followed by all author rebuttals in order by version.

Version 0:

Reviewer comments:

Reviewer #1

(Remarks to the Author)
The manuscript by Li et al., describes a web-based toolkit to incorporate experimental results including CLASH, miRNA-seq, mRNA-seq data from human, mouse, fly and nematode. The authors produced 90 new CLASH datasets with various cell lines. For some cell lines, small RNA data of knockouts of the TDMD factor ZSWIM8 are included. The dataset allowed the authors identify ATP6V1G1 as the TDMD trigger for miR-335-3p, which the authors previously reported to be upregulated in ZSWIM8 knockouts, demonstrating the utility of this resource. This has the potential to become a great resource, however, the manuscript and the website can be improved for clarity and broader utility. In particular, I feel that the database and tools could be more attractive to broader users by allowing them to browse results for larger numbers of genes more easily.

Major points:

1) The miR-335-ATP6V1G1 CLASH-tags are described in detail. Did the authors find any other potential triggers for other miRNAs upregulated in ZSWIM8-KOs? Even with negative or inconclusive results, I would hope to see such information as a reader. Please consider including some results of such bioinformatics analysis.

2) Currently, the utility of database section is limited because the user needs to input species, data type and gene or even cell type in the case of CLASH. This means that the user needs to know which miRNA or target gene they are interested in, and then check the miRNA page one by one to see in which cell lines or tissues the miRNA is expressed well enough. For miRNA-centric studies, researchers may want to look at many miRNAs that are expressed well and look for targets in any of the cell lines. How about showing miRNA expression in a tabular format with expression values in each sample shown. in it. A link can be embedded in each cell of the table so the user can go to the corresponding CLASH page by clicking the number. This way, miRNA centric browsing may be more efficient. For coding-gene centric studies, I imagine that allowing the user to select sets of genes using the user’s custom lists or maybe gene ontology information might be useful.

Minor points
1) I was a little confused about the origin of the ZSWIM8 knockouts. Do some of them come from their previous study? Table S1 contains a list of libraries, but the references appear to be for the CLASH libraries not for the cell lines. A list of cell lines can be provided as another supplementary table. If there are cell lines generated in this study, experimental validation results and the methods information should be provided in the paper. The method information is also missing for the ATP6V1G1 target site mutants (Figure S8B).

3) The ATP6V1G1-miR335 interaction seems consistent across many cell types (Figure 7C) but no upregulation of miR-335 is seen in HCT116 ZWISM8 knockout. If possible, please interpret this result.

4) Database tutorials: The links to both Database and Analysis parts appear only on the “Database” tab. It would be nice to have it on the “Analyzer” tab, too.

5) Not many small RNA-seq library kits use UMIs, and various patterns (locations and # of randomized nucleotides) of UMIs are also used. The analyzer route allows users to import AQ-seq data but it is unclear how flexible this tool is. A brief description is needed on the website to explain the requirements for the library design and how different types of small RNAseq data should be submitted.

6) Is the CLASH data track loaded on the UCSC genome browser? I am not sure if I just could not find it. If not provided, please consider including such a track so the users can also know which isoforms can be targets. Also, the miRNA gene search function should be tighter. When I search mir-21, I also get miR-210, etc. I even got a memory error when searching for miR-1, probably because the machine tries to load all miRNAs starting with miR-1xx.

7) What is the main difference between qCLASH and chimeric eCLIP? Authors performed both qCLASH and chimeric eCLIP sometimes even for a single cell line (e.g. A549, TableS1)? Direct comparisons of these datasets may be useful for readers to know the differences better.

8) For the cumulative fraction curve generator, the “404 Not Found” message shows up when no data is available. Instead of this, indicating reasons of no data (such as a page just saying "No CLASH data available") would be helpful for users. In addition, would it be possible to analyze this for targets of all miRNA genes and provide a link to the chart for each miRNA gene? (I do not know how computationally expensive such analysis would be, though.)

(Remarks on code availability)

Reviewer #2

(Remarks to the Author)
This manuscript presents the development of CLASHub, a comprehensive database that integrates both previously available and newly generated CLASH datasets together with matched miRNA and gene expression profiles. The platform enables systematic interrogation of miRNA–target interactions, providing resources to study not only how specific miRNAs regulate their targets, but also how certain targets can promote miRNA degradation through the target RNA-directed miRNA degradation (TDMD) pathway. A key strength of CLASHub is the inclusion of datasets from ZSWIM8 knockout models, in which the TDMD pathway is abolished, thereby facilitating the identification of candidate TDMD triggers for individual miRNAs. In addition, the database features a user-friendly Analyzer interface, which enables both exploration of existing datasets and standardized analysis of user-submitted CLASH datasets, including RNA-seq, miRNA-seq, and cumulative fraction curve visualization. To highlight its utility, the authors used CLASHub to uncover and experimentally validate a novel TDMD trigger for miR-335-3p within the 3′ UTR of ATP6V1G1.
A major strength of this study is the scale and comprehensiveness of the datasets. The authors generated 90 new CLASH datasets from 17 cell types and tissues and integrated them with existing data to create a resource spanning 27 cell types across human, mouse, Drosophila, and C. elegans. Importantly, the database aggregates matched miRNA expression, gene expression, and CLASH hybrid reads, and uniquely includes paired wild-type and ZSWIM8 knockout samples, which greatly enhances its power for identifying TDMD triggers. By automating the full analytical workflow through the Analyzer interface, CLASHub advances beyond prior tools, making miRNA–target analyses both robust and broadly accessible.
Despite its value, there are limitations that, if addressed, could further enhance the impact of CLASHub. First, the database does not fully exploit its paired datasets, which combine CLASH hybrid reads with miRNA and gene expression data. Currently, these data layers are presented in parallel rather than integrated. A more powerful approach would be to use expression data to prioritize and validate hybrid interactions. For example, highlighting target sequences that are enriched in hybrid reads despite relatively low expression, which would provide stronger evidence of authentic repression by a miRNA. A similar strategy could aid in identifying bona fide TDMD miRNA–trigger pairs, and implementing a ranking system for hybrid reads based on such integrative metrics would significantly strengthen the platform. Second, while hybrid reads can be used both to identify repressed targets of miRNAs and to identify TDMD triggers, CLASHub is currently more optimized for the latter. Since most users will be primarily interested in discovering authentic miRNA targets, the platform would benefit from additional features supporting this task, such as tools to identify conserved hybrid reads across models or to highlight cell type–specific interactions.
Other Suggestions
1. The authors demonstrate the utility of CLASHub through the identification of a novel TDMD trigger for miR-335-3p. It would further strengthen the platform if they also provided an example showcasing how CLASHub can be used to identify novel miRNA targets.
2. As noted above, the Analyzer tab would benefit from integrating multiple data layers and balancing functionality between identifying miRNA targets and TDMD triggers, thereby expanding its utility for a broader range of use cases.
3. In the current Database tab, results are displayed as long tables split across pages. Implementing a filtering system (e.g., by target site type or genomic region) would improve usability and allow users to narrow results to features of interest.
4. Incorporating visual outputs, such as coverage plots for hybrid target sites, would facilitate the identification of enrichment patterns relative to background noise and better guide experimental validation.
5. A deeper analysis of the aggregated CLASH datasets would be valuable for evaluating and optimizing the method. Addressing known biases, such as miRNA–target binding stability, seed sequence preferences, or other determinants, across cell lines could provide a more robust assessment of dataset quality.
6. Finally, the authors might consider discussing future applications of large language models (LLMs) to interact with aggregated datasets, which could open new opportunities for exploratory analyses.

(Remarks on code availability)

Reviewer #3

(Remarks to the Author)
I co-reviewed this manuscript with one of the reviewers who provided the listed reports. This is part of the Nature Communications initiative to facilitate training in peer review and to provide appropriate recognition for Early Career Researchers who co-review manuscripts.

(Remarks on code availability)

Reviewer #4

(Remarks to the Author)
The authors present CLASHub, an online platform that integrates CLASH, miRNA-seq, and RNA-seq data to study miRNA activity. Different cell and tissue types from multiple model organisms are represented, using both already published and newly generated data, including ZSWIM8-knockout data to facilitate study of target RNA-directed miRNA degradation. CLASHub aims to characterize miRNA activity, including distinguishing between isoforms. The method uses integration of validated miRNA-mRNA association data, miRNA expression levels, and expression levels of targets. CLASHub is useful both for scientists who do not have a background in bioinformatics as well as to streamline studies of miRNA activity.

CLASHub has potential to enable robust assessment of miRNA activity, but the manuscript currently lacks clarity regarding how the platform was constructed, expected false positives and negatives, and reproducibility. Thus, as currently presented, the efficacy of the method is difficult to assess. The utility of CLASHub could also increase with further customizability. Below, are areas that require attention.

1. An explanation of how the data was integrated and standardized would increase confidence for potential users, especially those that are familiar with bioinformatics and doing similar processes to those performed by CLASHub on their own. Similarly, how were the CLASH, RNA-seq, and miRNA-seq datasets selected to ensure quality and avoid bias?
2. Is it necessary for the method to rely on AQ-seq data? How does the exclusion of standard smRNA-seq impact the pipeline? While limiting to AQ-seq data could be argued to increase accuracy, the plethora of smRNA-seq datasets that are not AQ-seq may add insights missed by this limitation.
3. How would CLASHub handle variability and discrepancies in datasets used for input? Were datasets excluded to avoid this?
4. Some researchers may want further specificity in the model – could CLASHub enable different metrics from TargetScan, such as context score thresholds, rather than simply conserved versus nonconserved targets?
5. Indicated “limited reproducibility across samples” in line 322 – but this could just be differences in cell lines.
6. In Figure 7B and S8A, one nucleotide is not complementary – how is this indicated as 10 consecutive nucleotides in the text?
7. In figures 7D and E, showing significance testing would be useful for more than just one miRNA, especially since the lack of upregulation in passenger strands is less clear for the miRNAs where significance testing not shown for.
8. Adding the option to process the RNA-seq input with exon-intron split analysis (Gaidatzis et al Nature Biotechnology, 2015) could substantially decrease false positives.

(Remarks on code availability)

Version 1:

Reviewer comments:

Reviewer #1

(Remarks to the Author)
The authors addressed all the points I raised in my previous review.
With the improved interface, new findings powered by it and more detailed materials information, I believe this manuscript is suitable for publication in Nature Communications.

(Remarks on code availability)

Reviewer #2

(Remarks to the Author)
The authors have addressed the concerns raised in the initial review satisfactorily. The revisions and responses are clear and appropriate, and I have no further major concerns. I support publication of the manuscript in its current form.

(Remarks on code availability)

Reviewer #3

(Remarks to the Author)
I co-reviewed this manuscript with one of the reviewers who provided the listed reports. This is part of the Nature Communications initiative to facilitate training in peer review and to provide appropriate recognition for Early Career Researchers who co-review manuscripts.

(Remarks on code availability)

Reviewer #4

(Remarks to the Author)
The authors have responded effectively, and reasonably comprehensively to the original reviews, and their detailed responses are appropriate. The revised manuscript will be of some interest to the miRNA research community (and others). I recommend that the paper be accepted for publication.

(Remarks on code availability)

Open Access This Peer Review File is licensed under a Creative Commons Attribution 4.0 International License, which permits use, sharing, adaptation, distribution and reproduction in any medium or format, as long as you give appropriate credit to the original author(s) and the source, provide a link to the Creative Commons license, and indicate if changes were made.
In cases where reviewers are anonymous, credit should be given to 'Anonymous Referee' and the source.
The images or other third party material in this Peer Review File are included in the article’s Creative Commons license, unless indicated otherwise in a credit line to the material. If material is not included in the article’s Creative Commons license and your intended use is not permitted by statutory regulation or exceeds the permitted use, you will need to obtain permission directly from the copyright holder.
To view a copy of this license, visit https://creativecommons.org/licenses/by/4.0/

REVIEWER COMMENTS

Reviewer #1 (Remarks to the Author):

The manuscript by Li et al., describes a web-based toolkit to incorporate experimental results including CLASH, miRNA-seq, mRNA-seq data from human, mouse, fly and nematode. The authors produced 90 new CLASH datasets with various cell lines. For some cell lines, small RNA data of knockouts of the TDMD factor ZSWIM8 are included. The dataset allowed the authors identify ATP6V1G1 as the TDMD trigger for miR-335-3p, which the authors previously reported to be upregulated in ZSWIM8 knockouts, demonstrating the utility of this resource. This has the potential to become a great resource, however, the manuscript and the website can be improved for clarity and broader utility. In particular, I feel that the database and tools could be more attractive to broader users by allowing them to browse results for larger numbers of genes more easily.

We thank the reviewer for the positive comments and constructive suggestions.

Major points:

1) The miR-335-ATP6V1G1 CLASH-tags are described in detail. Did the authors find any other potential triggers for other miRNAs upregulated in ZSWIM8-KOs? Even with negative or inconclusive results, I would hope to see such information as a reader. Please consider including some results of such bioinformatics analysis.

This is a valuable point. In addition to the experimentally validated TDMD trigger of miR-335 for *ATP6V1G1*/*Atp6v1g1*, we now have systematically explored potential TDMD triggers across available CLASH datasets.

Focusing on the list of known ZSWIM8-sensitive miRNAs (Donnelly et al., 2022; Jones et al., 2023; Lu Li et al., 2021; Shi et al., 2023; Shi et al., 2020), we extracted all miRNA-target hybrids that met potential TDMD structural criteria: (1) a canonical seed match (6mer, 7mer, or 8mer) and (2) at least eight contiguous base pairs located within the last 10 nucleotides of the miRNA 3′ end. The results of this search are provided in Table S12 and are described in lines 338-342.

This analysis successfully recovered several known TDMD interactions, including the recently reported: miR-322-5p:*Plagl1*, miR-503:*Malat1*, and miR-335:*Lpar4* in mouse (LaVigne et al., 2026; Lin et al., 2025), as well as the miR-37/38:*tts-2* interaction in *C. elegans* (Grimme et al., 2025). Furthermore, the table includes all other candidate TDMD interactions, providing readers with a complete dataset of potential TDMD triggers—including those with lower hybrid counts that may represent rare or cell-type-specific events. We also add this description in the Discussion (lines 461-467).

2) Currently, the utility of database section is limited because the user needs to input species, data type and gene or even cell type in the case of CLASH. This means that the user needs to know which miRNA or target gene they are interested in, and then check the miRNA page one by one to see in which cell lines or tissues the miRNA is expressed well enough. For miRNA-centric

studies, researchers may want to look at many miRNAs that are expressed well and look for targets in any of the cell lines.

A link can be embedded in each cell of the table so the user can go to the corresponding CLASH page by clicking the number. This way, miRNA centric browsing may be more efficient. For coding-gene centric studies, I imagine that allowing the user to select sets of genes using the user's custom lists or maybe gene ontology information might be useful.

To address the need for broader searching, we have generated CLASH tables that merge all available cell lines for a given species into a single searchable dataset. This allows users to search for a specific miRNA (e.g., miR-7) or a specific target gene and immediately see if the interaction exists across multiple cell lines, without navigating to each cell line individually (screenshot below — dashed-line area). All of these improvements are now incorporated into the updated CLASHub interface and are described in the revised manuscript (see lines 141-144).

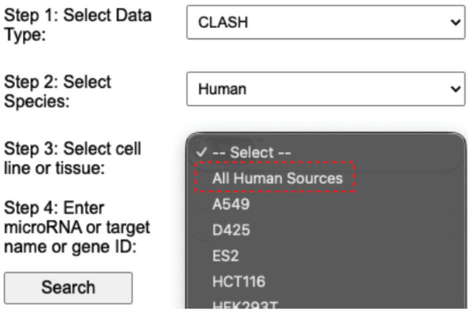

CLASH: miR-7-5p in All Human Sources

Filter by Region: All Regions | Filter by Site Type: All Site Types | Download Table

miRNA Name	Pairing Pattern (UNAfold)	Gene Name	Gene ID	Conservation Score	Free Energy (kcal/mol)	Gene Type	Target Site Region	Genome Position	Target Site Type	Total Cell Lines Out of 15 Tissues/Lines	Specific Cell Lines (Click to view details)
miR-7-5p	miRNA: 5' UGGAAGACUA--GUGAUUUUGUUGUU<br>||||||| |||||||||||||| target: 3' ACCUUCUGUAACCACUAAAACAACAA	OIP5-AS1	ENSG00000247556	6.53	-27.6	lncRNA	NULL	chr15:41300574-41300599	8mer	15	501Mel, A549, Colorectal, D425, ES2, H1299, HCT116, HEK293T, HepG2, MB002, MDA-MB-231, Ovcar8, T98G, TIVE, U87MG

The reviewer also suggested allowing users to input "custom lists" or "sets of genes" directly on the web. Because our search bar uses an auto-fill feature that strictly matches user input to existing miRNA or gene names to ensure data accuracy, allowing "batch searches" or custom lists often leads to input errors (e.g., typos, gene synonyms, or formatting issues) that return false negatives. We therefore decided to maintain a single-query interface with auto-fill functionality for the web tool. As an alternative solution, users who need to analyze "sets of genes" or perform Gene Ontology (GO) analysis could use the "Bulk Download" feature on the CLASHub website we have provided (https://clashub.rc.ufl.edu/more_info/bulk_download.html) (See the screenshot below — dashed-line area). This allows researchers to download the comprehensive, processed data and analyze them with their own pipelines (e.g., DAVID, clusterProfiler) without restrictions. (Methods section, lines 1031-1034).

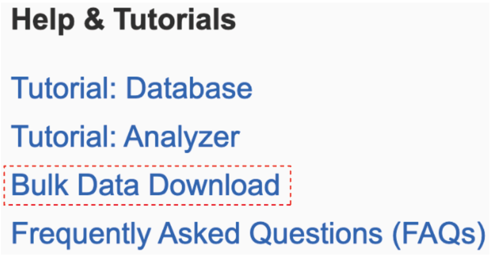

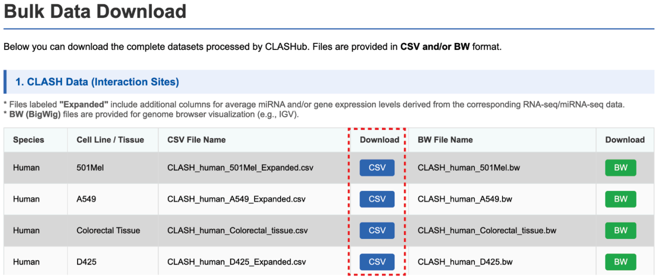

Minor points

1) I was a little confused about the origin of the ZSWIM8 knockouts. Do some of them come from their previous study? Table S1 contains a list of libraries, but the references appear to be for the CLASH libraries not for the cell lines. A list of cell lines can be provided as another supplementary table. If there are cell lines generated in this study, experimental validation results and the methods information should be provided in the paper. The method information is also missing for the ATP6V1G1 target site mutants (Figure S8B).

We have addressed this comment by providing a comprehensive list of resources and adding experimental validation data.

To clarify cell line origins, we have added Supplementary Table S4, which explicitly lists the source, genotype, and validation method for all cell lines and tissues used in this study, including *ZSWIM8* KO lines and *ATP6V1G1*/*Atp6v1g1* TDMD trigger deletion lines. Some lines were obtained from previous studies, including MEFs (Han et al., 2020) and U87MG (Li et al., 2021). The majority of *ZSWIM8*-knockout cell lines (A549, H1299, MDA-MB-231, ES2, OVCAR8, T98G, 501Mel, MB002, and HCT116) and colorectal tissue were generated in this study. For each cell line, two independent *ZSWIM8*-targeting sgRNAs (sgRNA1 and sgRNA2) and one non-targeting control sgRNA were used, yielding three stable lines per parental cell line (sgRNA1 KO, sgRNA2 KO, and non-targeting control). We confirmed the loss of *ZSWIM8* function by analyzing miR-7 (a known ZSWIM8/TDMD substrate) abundance via Northern blotting. As shown in Figure S1F, we observed consistent upregulation of miR-7 in both sgRNA1 and sgRNA2 knockout clones compared to controls (see Results, lines 132-135; Methods, lines 734–745).

For the *ATP6V1G1*/*Atp6v1g1* TDMD trigger deletions, two sgRNAs flanking the trigger region were co-delivered simultaneously to delete the genomic segment in MDA-MB-231 (human) and MEF (mouse) cells. Single-cell clones were isolated and expanded, and homozygous deletions were confirmed by genotyping PCR and Sanger sequencing (Figure S9B-S9C). Detailed methods, including sgRNA sequences and genotyping primers, are now provided in the manuscript (see Methods, lines 746–763).

3) The ATP6V1G1-miR335 interaction seems consistent across many cell types (Figure 7C) but no upregulation of miR-335 is seen in HCT116 ZWISM8 knockout. If possible, please interpret this result.

The reviewer is correct that the upregulation of miR-335 hybrids in HCT116 ZSWIM8-knockout cells is less pronounced compared to the trends observed in other cell lines. For this revision, we made minor modifications to the CLASHub pipeline code and performed CPM normalization of all miRNA hybrids in each cell line. For the revised Figure 7C, we also performed multiple unpaired t-tests to evaluate in which cell lines the miR-335-3p:*ATP6V1G1* interaction showed statistically significant changes. Compared to the previous version, we now detected miR-335-3p:*ATP6V1G1* hybrids in a total of nine human cell lines and one mouse tissue (kidney). Significant increases upon ZSWIM8 knockout were observed only in A549 (qCLASH), H1299, and MB002. In HCT116 and several other cell lines, although the miR-335-3p:*ATP6V1G1* hybrid was detected, its absolute abundance was low, and the difference between control and knockout conditions did not reach statistical significance. This is a known limitation of CLASH-based approaches: chimeric reads are inherently rare, and low baseline abundance limits the statistical power to detect significant changes, even when a directional trend may be present. We have revised the manuscript text and Figure 7C to include the statistical analysis and to more precisely describe these results (see revised manuscript, lines 350–360).

4) Database tutorials: The links to both Database and Analysis parts appear only on the "Database" tab. It would be nice to have it on the "Analyzer" tab, too.

We have updated the "Analyzer" interface to include a dedicated "Help & Tutorials" section in the left-hand navigation sidebar. This ensures consistency with the Database page and makes it significantly easier for users to locate the necessary tutorials. See screenshot below — dashed-line area. (https://clashub.rc.ufl.edu/Analyzer.html):

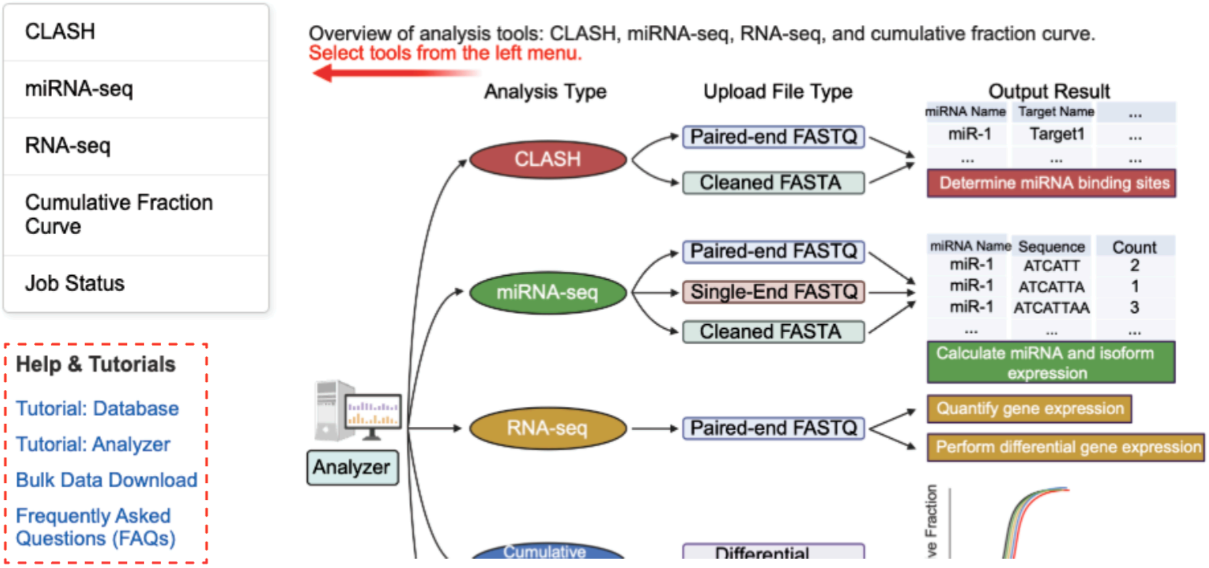

5) Not many small RNA-seq library kits use UMIs, and various patterns (locations and # of randomized nucleotides) of UMIs are also used. The analyzer route allows users to import AQ-seq data but it is unclear how flexible this tool is. A brief description is needed on the website to explain the requirements for the library design and how different types of small RNAseq data should be submitted.

Based on this comment and Reviewer 4's comment #2, we improved the miRNA-seq Analyzer to accommodate diverse UMI configurations across different library preparation protocols. We have updated the miRNA-seq Analyzer interface to allow users to specify 5′ and 3′ UMI lengths. For AQ-seq data, users enter the appropriate UMI lengths (e.g., 4 bp at each end), and CLASHub performs PCR duplicate collapsing followed by UMI removal. For standard small RNA-seq libraries that do not utilize UMIs (e.g., Illumina TruSeq, NEBNext), users can set both values to 0, which triggers CLASHub to automatically skip the deduplication and UMI-trimming steps. This design also accommodates kits with non-standard or asymmetric UMI patterns.

As suggested, we have also updated the schematic diagram and description on the submission page (Figures S5 and S6). In "miRNA-Seq Data Analysis" section (lines 263-268) and in Methods (lines 923-939), we describe the processing workflow and how user input of UMI lengths control which steps are applied.

6) Is the CLASH data track loaded on the UCSC genome browser? I am not sure if I just could not find it. If not provided, please consider including such a track so the users can also know which isoforms can be targets. Also, the miRNA gene search function should be tighter. When I search mir-21, I also get miR-210, etc. I even got a memory error when searching for miR-1, probably because the machine tries to load all miRNAs starting with miR-1xx.

We appreciate the reviewer's suggestion regarding data visualization. We would like to clarify that the platform already supports isoform identification without the need to load raw data tracks. The "Genome Position" links in our result tables utilize the UCSC "Highlight" function. Clicking the link directs users to the specific locus with the miRNA binding site highlighted in red against reference gene models (e.g., Ensembl/GENCODE). This visual cue allows users to immediately discern which specific exon or 3' UTR region—and consequently which isoform—is targeted (Figure 2B and S3C) (lines 188-195).

To further address the reviewer's request for inspecting raw data tracks, we have established a "BigWig Data Download Page" (https://clashub.rc.ufl.edu/more_info/bulk_download.html) which provides BigWig files for each cell line/tissue for users (See the screenshot below — dashed-line area). Since CLASHub hosts a large volume of datasets (often >6 replicates per condition), automatically loading all tracks into UCSC would cause significant latency. Therefore, providing one processed bigWig file from each cell line for download is the optimal solution, enabling users to selectively load and visualize coverage peaks in their preferred browsers (e.g., IGV or UCSC) for detailed inspection (track example in Figure S4C) (lines 247-255). A related point was brought up in Reviewer 2's minor point #4, if interested, detailed description of how "peak-calling" is performed is described there.

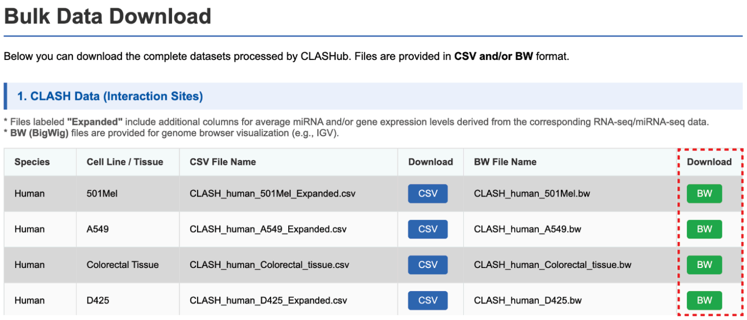

Bulk Data Download

Below you can download the complete datasets processed by CLASHub. Files are provided in **CSV and/or BW** format.

1. CLASH Data (Interaction Sites)

* Files labeled **"Expanded"** include additional columns for average miRNA and/or gene expression levels derived from the corresponding RNA-seq/miRNA-seq data.
* **BW (BigWig)** files are provided for genome browser visualization (e.g., IGV).

Species	Cell Line / Tissue	CSV File Name	Download	BW File Name	Download
Human	501Mel	CLASH_human_501Mel_Expanded.csv	CSV	CLASH_human_501Mel.bw	BW
Human	A549	CLASH_human_A549_Expanded.csv	CSV	CLASH_human_A549.bw	BW
Human	Colorectal Tissue	CLASH_human_Colorectal_tissue.csv	CSV	CLASH_human_Colorectal_tissue.bw	BW
Human	D425	CLASH_human_D425_Expanded.csv	CSV	CLASH_human_D425.bw	BW

Regarding the search function, we have fully addressed the issue regarding loose search results (e.g., miR-21 vs. miR-210) and potential memory errors. We have modified both the frontend (JavaScript) and backend (PHP) logic of CLASHub to enforce "Exact Matching**"**. The search interface now requires users to select a specific, complete miRNA or gene name from the dropdown menu. We have removed the previous "fuzzy search" (relaxed format) capability. (See the screenshot below — dashed-line area).

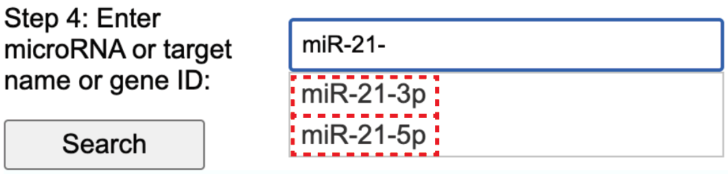

7) What is the main difference between qCLASH and chimeric eCLIP? Authors performed both qCLASH and chimeric eCLIP sometimes even for a single cell line (e.g. A549, TableS1)? Direct comparisons of these datasets may be useful for readers to know the differences better.

We thank the reviewer for this important question. The key distinction between qCLASH and chimeric eCLIP lies in two aspects: adapter ligation strategy and RNA size-selection.

In qCLASH, both the 3′ and 5′ adapters are ligated to RNA prior to reverse transcription. This all-RNA-level ligation workflow is streamlined and well-suited for samples with limited starting material, as it does not require gel electrophoresis and transfer to membrane to size-select hybrids. However, the absence of size-selection means that AGO-bound complexes are not separated from non-specific RNA–protein complexes or free RNA, resulting in a higher proportion of non-hybrid background reads (Gay et al., 2018).

In chimeric eCLIP, the 3′ adapter is ligated at the RNA level, but the 5′ adapter is ligated to cDNA after reverse transcription. This DNA-level ligation is inherently more efficient than RNA ligation according to Van Nostrand et. al (Nat. Methods, 2016), yielding higher library complexity. Furthermore, chimeric eCLIP can incorporate an SDS-PAGE and nitrocellulose membrane transfer step to isolate AGO–RNA complexes within a defined molecular weight range (100–250 kDa), effectively enriching for on-target chimeric reads. In this study, gel size-selection was applied to the majority of our chimeric eCLIP samples (A549, HCT116, HEK293T, MDA-MB-

231, mouse heart, kidney, and MEF), with the exception of human colorectal tissue, which was processed without size-selection due to limited material availability (Table S1). Both protocols are now described in full procedural detail in the revised Methods section (Lines 764–842).

A549 and MDA-MB-231 cells were each subjected to 6 replicates of qCLASH and 6 replicates of chimeric eCLIP under matched conditions. This comparison revealed that chimeric eCLIP recovers significantly more unique chimeric reads than qCLASH in both cell lines (Figure S1G), consistent with the advantages of DNA-level adapter ligation and gel-based size-selection described above. Despite these quantitative differences, the two methods identify largely overlapping sets of miRNA–target interactions, supporting the reliability of both approaches. We have added this comparison to the Results section (Lines 149–159) to help readers evaluate the relative performance of each method.

8) For the cumulative fraction curve generator, the “404 Not Found” message shows up when no data is available. Instead of this, indicating reasons of no data (such as a page just saying "No CLASH data available") would be helpful for users. In addition, would it be possible to analyze this for targets of all miRNA genes and provide a link to the chart for each miRNA gene? (I do not know how computationally expensive such analysis would be, though.)

To address this, we have significantly updated our backend code to ensure robust error handling and transparent user feedback.

Automated Input Validation: The system now automatically verifies the presence of essential columns (e.g., GeneName, baseMean, log2FoldChange) in the input file. If required data is missing, instead of triggering a system error (which previously caused the “404 message”), the tool now generates an informative SVG image that explicitly alerts the user to the specific missing columns (see screenshot below).

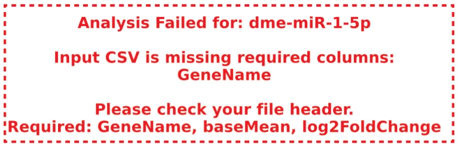

Handling of Insufficient Data: We have implemented a statistical threshold (n<10) for curve generation. If a specific dataset (e.g., CLASH targets or TargetScan conserved targets) contains too few data points, the system no longer fails. Instead, it generates the plot using the remaining valid data and appends an explanatory note at the bottom of the figure (e.g., *"Exclusion Note: CLASH targets excluded due to low count"*). This ensures users understand exactly why certain data lines may be absent.

Error Reporting via CSV: We have ensured that the CSV download link remains functional under all conditions. In cases of input errors or insufficient data, the downloadable CSV now contains specific error details or status reports instead of a broken link (404 error), allowing users to diagnose issues immediately (see screenshot below).

Analysis_Status	Error_Reason	Missing_Columns
Failed	Input CSV missing required columns	{'GeneName'}

Regarding the suggestion to extend this analysis to all miRNA genes simultaneously, we note that the cumulative fraction curve analysis is intentionally designed to evaluate the effect of a single miRNA perturbation on its predicted targets. As described in the Methods, the module requires users to upload differential gene expression data derived from a specific experimental condition — such as a miRNA knockout or overexpression — in which one miRNA is perturbed at a time. Analyzing multiple miRNAs simultaneously would confound the interpretation, as distinct miRNA target sets may overlap or exert opposing regulatory effects. Therefore, the single-miRNA framework is retained by design, as it best reflects the biological question being addressed.

Reviewer #2 (Remarks to the Author):

This manuscript presents the development of CLASHub, a comprehensive database that integrates both previously available and newly generated CLASH datasets together with matched miRNA and gene expression profiles. The platform enables systematic interrogation of miRNA–target interactions, providing resources to study not only how specific miRNAs regulate their targets, but also how certain targets can promote miRNA degradation through the target RNA-directed miRNA degradation (TDMD) pathway. A key strength of CLASHub is the inclusion of datasets from ZSWIM8 knockout models, in which the TDMD pathway is abolished, thereby facilitating the identification of candidate TDMD triggers for individual miRNAs. In addition, the database features a user-friendly Analyzer interface, which enables both exploration of existing datasets and standardized analysis of user-submitted CLASH datasets, including RNA-seq, miRNA-seq, and cumulative fraction curve visualization. To highlight its utility, the authors used CLASHub to uncover and experimentally validate a novel TDMD trigger for miR-335-3p within the 3′ UTR of *ATP6V1G1*.

A major strength of this study is the scale and comprehensiveness of the datasets. The authors generated 90 new CLASH datasets from 17 cell types and tissues and integrated them with existing data to create a resource spanning 27 cell types across human, mouse, Drosophila, and C. elegans. Importantly, the database aggregates matched miRNA expression, gene expression, and CLASH hybrid reads, and uniquely includes paired wild-type and ZSWIM8 knockout samples, which greatly enhances its power for identifying TDMD triggers. By automating the full analytical workflow through the Analyzer interface, CLASHub advances beyond prior tools, making miRNA–target analyses both robust and broadly accessible.

Despite its value, there are limitations that, if addressed, could further enhance the impact of CLASHub.

We thank the reviewer for the positive feedback and the suggestion to further enhance the utility of CLASHub.

First, the database does not fully exploit its paired datasets, which combine CLASH hybrid reads with miRNA and gene expression data. Currently, these data layers are presented in parallel rather than integrated. A more powerful approach would be to use expression data to prioritize and validate hybrid interactions. For example, highlighting target sequences that are enriched in hybrid reads despite relatively low expression, which would provide stronger evidence of authentic repression by a miRNA. A similar strategy could aid in identifying bona fide TDMD miRNA–trigger pairs, and implementing a ranking system for hybrid reads based on such integrative metrics would significantly strengthen the platform.

We have now incorporated matched miRNA and gene expression profiles directly into the database. For each dataset, we calculated the average abundance separately for control samples (Wild Type or sgNT) and ZSWIM8-knockout samples. These values are then presented together with the corresponding hybrid reads, as illustrated in the updated Figure 2 (lines 167-169). For example, in the A549 dataset, users can now view miRNA expression (CPM) and target gene expression (TPM) directly within the results table, enabling immediate cross-referencing of interaction evidence with expression context.

In the current version, users can sort the results table online by multiple columns, including normalized miRNA or gene abundance, target site type, gene type, free energy, and conservation score, among others (Figure S2, lines 171-173). Regarding reviewer's comment, we considered implementing a single "integrative ranking metric", such as the ratio between hybrid read counts and miRNA or gene abundance. However, because researchers approach CLASH data with diverse analytical goals — for instance, identifying candidate TDMD triggers versus validating canonical miRNA-target repression — a single ratio-based ranking could be misleading or inapplicable across different use cases. For TDMD discovery specifically, the most informative metric would involve comparing miRNA abundance between wild-type and ZSWIM8-knockout conditions, whereas for target validation, hybrid enrichment relative to gene expression would be more relevant.

To accommodate this diversity, CLASHub offers two download options. First, users can click the 'Download Table' button within any search result (Figure 2B, #9) to export the displayed CLASH table for a specific miRNA or gene query, including hybrid reads, target gene expression, and miRNA abundance. Second, CLASHub provides a Bulk Download page (screenshots below, lines 1031-1034, https://clashub.rc.ufl.edu/more_info/bulk_download.html) where users can download complete processed datasets for an entire cell line or tissue, covering all miRNAs and all target genes. Users can then apply custom ranking metrics or filtering thresholds best suited to their specific research questions.

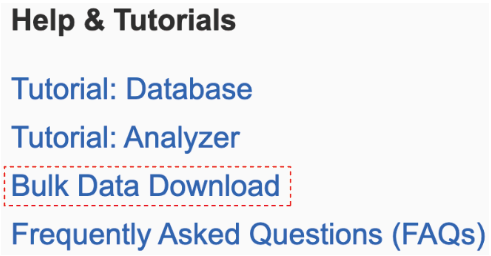

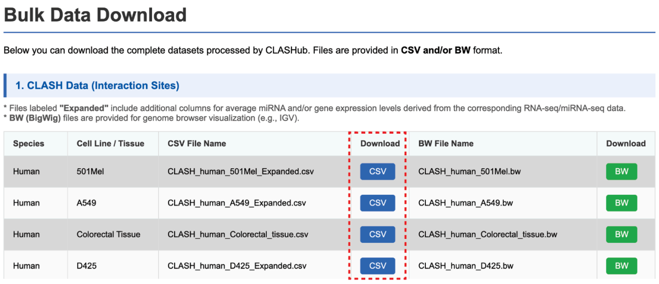

Second, while hybrid reads can be used both to identify repressed targets of miRNAs and to identify TDMD triggers, CLASHub is currently more optimized for the latter. Since most users will be primarily interested in discovering authentic miRNA targets, the platform would benefit from additional features supporting this task, such as tools to identify conserved hybrid reads across models or to highlight cell type–specific interactions.

To better support users interested in identifying authentic miRNA targets, we have implemented species-level aggregate tables. For each species with multiple datasets — Human (15 datasets), Mouse (7 datasets), and C. elegans (2 life stages). As one example, users can now select "All Human Sources" in the cell line/tissue dropdown menu. These aggregate tables report the total number of distinct cell lines or tissues in which each interaction was detected, along with the specific sample names, which are fully interactive — users can click any sample name to navigate directly to the detailed results for that individual dataset (screenshot below — dashed-line area) (lines 141-144).

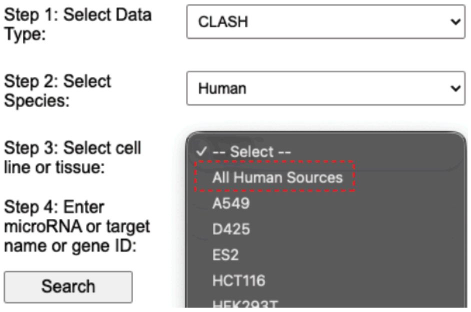

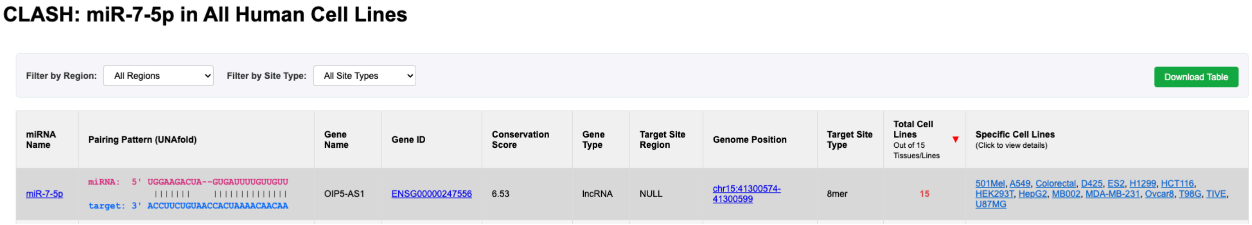

As the reviewer pointed out, interactions detected in limited numbers of cell lines may reflect genuine cell type-specific regulation rather than false positives. The biological specificity of miRNA-target interactions is informative, and users can evaluate such cases by integrating additional evidence from the matched expression data and pairing features (e.g., site type, free energy, conservation score) provided in the table.

Regarding cross-species conservation of hybrid reads, we note that while the same miRNA family may be conserved across human, mouse, *Drosophila* and *C. elegans*, the target sequences often differ due to evolutionary divergence in 3' UTR sequences and gene orthologs, making direct cross-species merging of hybrid reads technically challenging and potentially misleading. Nonetheless, using the "download" function for specific miRNAs or "bulk download" of comprehensive hybrid lists from different species (see also response to Review 1 major point #2), users can query the same miRNA across species and compare target gene orthologs to assess conservation of interactions,

Other Suggestions

1. The authors demonstrate the utility of CLASHub through the identification of a novel TDMD trigger for miR-335-3p. It would further strengthen the platform if they also provided an example showcasing how CLASHub can be used to identify novel miRNA targets.

To address this, we leveraged CLASHub to screen for high-confidence canonical repression targets of miR-18a-5p by systematically filtering CLASH hybrids based on seed match type, 3'UTR localization, cross-cell-line reproducibility, and binding site conservation (Table S15). We then selected seven targets (*ATXN1L, FAM3C, NEDD4, PDE4D, RNF4, ZBTB4, and ZNF367*) that have not been reported previously for validation (Table 2). Introduction of miR-18a-5p mimic significantly repressed all seven targets at the mRNA level, while inhibition of endogenous miR-18a-5p led to significant upregulation of six targets, with PDE4D showing a similar trend that did not reach statistical significance (Figures 8A and 8B). To confirm direct binding between miRNA and the target site identified by the hybrid reads, we performed dual-luciferase assays by cloning the 3'UTR of three representative targets (*FAM3C*, *ZBTB4* and *ZNF367*) downstream of the luciferase reporter. The results showed that miR-18a-5p repression is dependent on the specific miRNA seed-binding sites identified by CLASHub, as mutation of these sites abolished the repressive effect (Figures 8C and 8D). These new findings confirm that CLASHub is highly effective at identifying *bona fide* canonical miRNA targets. We have included these data as a new Figure 8 in the revised manuscript and have updated the Abstract (line 37), Introduction (line 115), Results (lines 382-412), and Discussion (lines 468-473) sections to highlight this dual capability of the platform.

2. As noted above, the Analyzer tab would benefit from integrating multiple data layers and balancing functionality between identifying miRNA targets and TDMD triggers, thereby expanding its utility for a broader range of use cases.

We agree that integrating multiple data layers in the Analyzer — such as hybrid reads, miRNA expression, and gene expression — would further enhance the platform's utility. However, due to the inherent differences in data types and processing requirements, these analyses are better implemented as separate modules. A combination of CLASH hybrid, miRNA abundance and RNA-seq require mapping to separate reference databases, which is computationally intensive and time-consuming. Therefore, we currently kept these analyses separate within the Analyzer module to provide greater flexibility. We plan to monitor user feedback and will consider further integration of these data types in future versions to improve usability and support broader analytical goals.

3. In the current Database tab, results are displayed as long tables split across pages. Implementing a filtering system (e.g., by target site type or genomic region) would improve usability and allow users to narrow results to features of interest.

We agree that navigating extensive datasets can be challenging without efficient filtering tools. We have implemented a filtering system in the "Database" tab. Users can now filter the results

table by "Target Site Region" (e.g., 3' UTR, CDS, 5' UTR, Intron, Non-coding) and "Target Site Type" (e.g., 8mer, 7mer-m8, etc.) using drop-down menus. We have updated Figure 2B to illustrate these new filtering controls. We have revised the manuscript (lines 169-171) to describe this new functionality.

Additionally, all data in the database can be downloaded as .csv files (https://clashub.rc.ufl.edu/more_info/bulk_download.html). It allows users to locally perform in depth ranking and filtering using Excel according to different research questions.

4. Incorporating visual outputs, such as coverage plots for hybrid target sites, would facilitate the identification of enrichment patterns relative to background noise and better guide experimental validation.

To address this, we have enhanced the CLASHub data processing and visualization pipeline. The pipeline now automatically generates genome-wide coverage tracks in bigWig (.bw) format. These tracks allow users to visualize non-hybrid RNA reads from CLASH in genome browsers (e.g., IGV), providing an intuitive validation of enrichment patterns relative to the background (Figure S4C). Furthermore, we perform peak calling using Piranha to identify statistically significant miRNA-binding hotspots ($p < 0.01$) (Uren et al., 2012).

To better guide experimental validation, the CLASHub output table now includes two new columns: "Piranha peak p-value" and "Confidence" (Table S5). We have implemented a rigorous classification strategy to stratify interactions: “High Confidence interactions” are mapped to Ensembl-annotated mRNAs, lncRNAs or lincRNAs that satisfy one of the following criteria: 1. Canonical Seed Match**:** 8mer, 7mer, 6mer, or offset-6mer. or 2. Non-canonical**:** Interactions lacking canonical seeds must possess both significant peak (Piranha $p < 0.01$) and meet the strict structural geometry defined by Hall et al. (2025) [continuous seed match ≥ 4 nt, extensive 3′ pairing ≥ 10 nt, and offset (nt difference in unpair region in target and miRNA) -4 to +6 nt]. “Low Confidence Interactions” are mapped to potential target genes (mRNA/lncRNA/lincRNA) that lack canonical seeds and fail to meet the statistical or structural requirements for high confidence. “Background Interactions” are mapped to noise-prone RNA classes (e.g., rRNA, tRNA, pseudogenes) that are not mRNAs, lncRNAs or lincRNAs. An example of target sequence distribution after “Analyzer” processing is given in Figure S4B.

These updates provide users with both quantitative statistical support and qualitative visual validation for each identified hybrid. The Results section (lines 247–255) and the Methods section (lines 858-882) have been updated.

5. A deeper analysis of the aggregated CLASH datasets would be valuable for evaluating and optimizing the method. Addressing known biases, such as miRNA–target binding stability, seed sequence preferences, or other determinants, across cell lines could provide a more robust assessment of dataset quality.

In the original CLASHub construction, we have included all publicly available CLASH datasets without screening. We reasoned that a comprehensive list of hybrid reads would allow users to unbiasedly screen for *bona fide* miRNA-target interactions. Seeing this comment and Reviewer

4's comments #1 and #3, we recognize that datasets of variable quality may introduce noise and reduce user confidence. We therefore performed a comprehensive update to the CLASHub analysis pipeline and re-analyzed all aggregated datasets.

A. Quality screen of CLASH datasets

To systematically evaluate dataset quality, we compared the number of unique miRNA–target hybrids across both our newly generated CLASH data and publicly available datasets. We found that 98% of our newly generated samples yielded more than 5,000 unique hybrids, suggesting that this benchmark is reliably achievable under optimized experimental conditions. In contrast, 33.7% of publicly available datasets fell below this threshold, likely reflecting variable library preparation quality in previous studies (figure below).

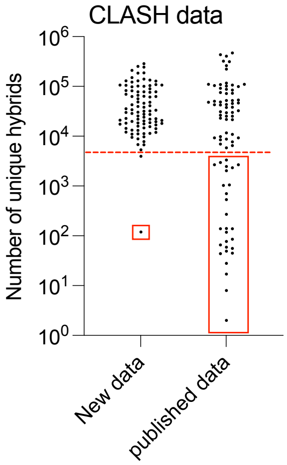

To validate that >5000 unique hybrid is sufficiently stringent to capture biologically meaningful interactions, we examined two well-established ubiquitous miRNA–target pairs: miR-7:OIP5-AS1 (Cyrano) and miR-92a:OIP5-AS1, both of which have been experimentally validated in prior studies (Kleaveland et al., 2018; Li et al., 2020 PMID:32497496; Shi et al., 2023). Reassuringly, both interactions were detectable across all 15 human cell lines and tissues retained in CLASHub after applying the quality cutoff of >5000 unique hybrids (screenshot below). While we acknowledge that not every *bona fide* interaction will be captured in every dataset, these results demonstrate that the current quality cutoff strikes a balance between data inclusivity and reliability, and is sufficient for users to identify interactions of interest.

CLASH: OIP5-AS1 in All Human Sources

Filter by Region: All Regions | Filter by Site Type: All Site Types | Download Table

miRNA Name	Pairing Pattern (UNAfold)	Gene Name	Gene ID	Conservation Score	Free Energy (kcal/mol)	Gene Type	Target Site Region	Genome Position	Target Site Type	Total Cell Lines Out of 15 Tissues/Lines	Specific Cell Lines (Click to view details)
miR-7-5p	miRNA: 5' UGGAAGACUA--GUGAUUUUGUUGUU<br>target: 3' ACCUUCUGUAACCACUAAAACAACAA	OIP5-AS1	ENSG00000247556	6.53	-27.6	lncRNA	NULL	chr15:41300574-41300599	8mer	15	501Mel, A549, Colorectal tissue, D425, ES2, H1299, HCT116, HEK293T, HepG2, MB002, MDA-MB-231, Ovcar8, T98G, TIVE, U87MG
miR-92a-3p	miRNA: 5' UAUUGCACUUGUCCCGGCCUGU<br>target: 3' AUAACGUGUAUAGGGCCAAAAU	OIP5-AS1	ENSG00000247556	5.01	-23.6	lncRNA	NULL	chr15:41300490-41300511	8mer	15	501Mel, A549, Colorectal tissue, D425, ES2, H1299, HCT116, HEK293T, HepG2, MB002, MDA-MB-231, Ovcar8, T98G, TIVE, U87MG

Based on the quality cutoff of >5,000 unique miRNA–target hybrids per CLASH sample, datasets falling below this threshold—indicated by the red dashed line and red boxes—were excluded from CLASHub (Figure below). Excluded datasets include Huh7.5 samples (Moore et al., 2015), liver tissue samples, and *C. elegans* L3 stage libraries (Grosswendt et al., 2014). One exception was made for a single HepG2 sample that fell marginally below the cutoff; this dataset was retained to preserve a complete set of three biological replicates for this cell line.

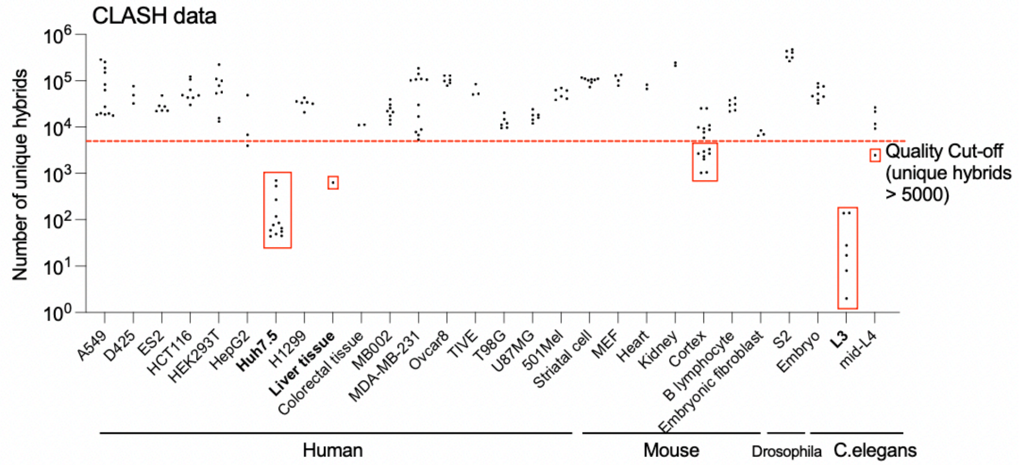

To further minimize potential bias, we required that each cell line or tissue represented in CLASHub be supported by at least two independent biological samples, with a mean of 5.84 samples per tissue/cell line across the 25 included cell lines and tissues (Table S1). We have expanded the Methods section to detail the dataset selection and construction of the CLASH database (lines 883-897).

If the users desire an unbiased view of all miRNA target interactions, a full list of un-filtered miRNA-target hybrids is available by processing the raw data through the "Analyzer" module.

B. Quality screen of RNA-seq datasets

To retain high-quality for RNA-seq data, we implemented filtering criteria that require >10 million aligned reads and a genome alignment rate >80% (See Methods lines 983-987).

In our initial dataset, several mouse RNA-seq samples originated from previous studies (e.g., Li et al., 2017) did not meet our sequencing depth requirements, with most falling between 1 million and 10 million aligned reads (Figure below, indicated by the red box in the "Aligned Reads" figure). Consequently, we excluded these and replaced them with higher-depth, recently published mouse RNA-seq data (e.g., Shi CY et al., 2023) that satisfied both the read count and alignment rate thresholds (Figures below, indicated by the green box). Beyond the mouse samples, additional datasets were removed based on specific quality failures: two MB002 samples were excluded for insufficient read depth; two T98G, two *Drosophila*, and three *C. elegans* samples were removed due to poor alignment rates; and two *C. elegans* samples were excluded for lacking sufficient biological replication. In total, 73 new mouse samples were added to replace the excluded data (Table S2).

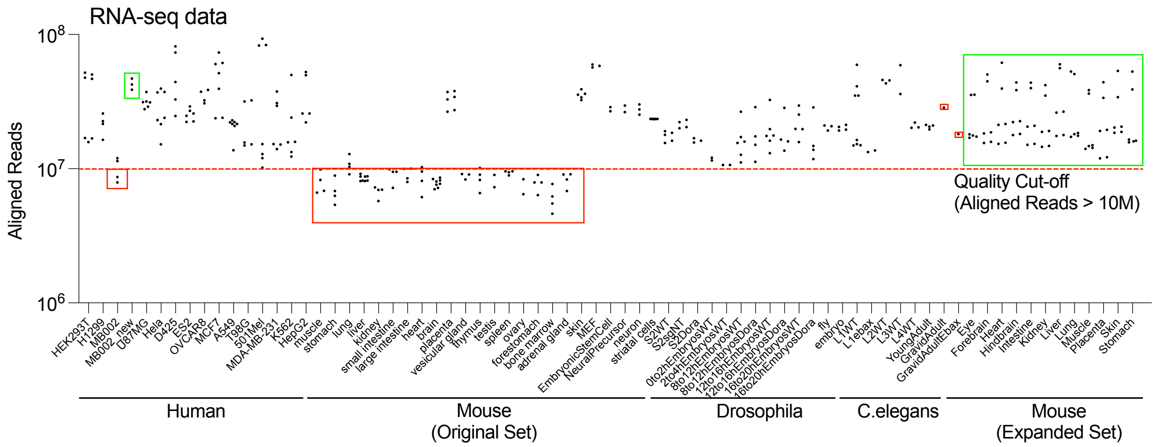

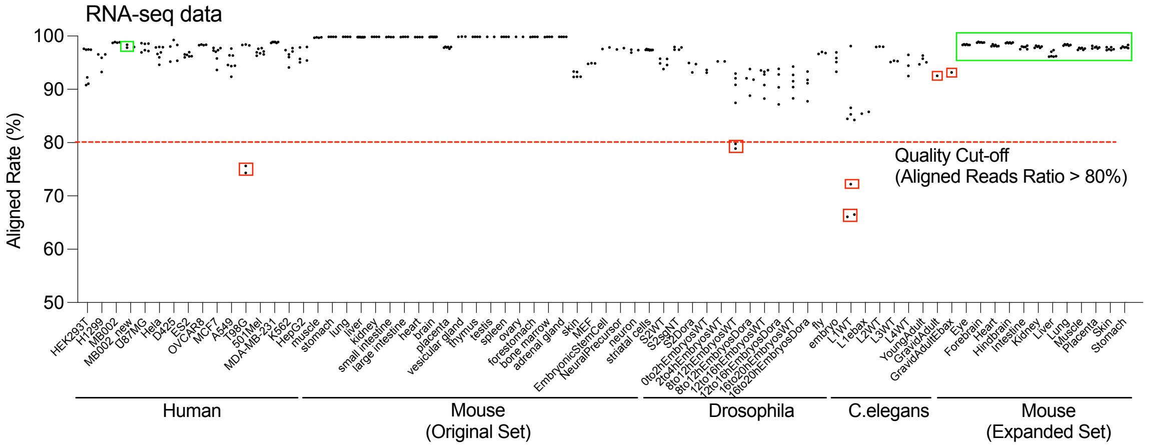

C. Quality screen of miRNA-seq datasets

To ensure high-quality quantification of miRNA abundance, we prioritized data generated via AQ-seq (Accurate Quantification by Sequencing). We selected datasets from Kim et al. (*Nucleic Acids Research*, 2020), Shi et al. and Han et al. (*Science*, 2020), Jones et al. (*Genes & Development*, 2023), Stubna et al. (*RNA*, 2024), and Li et al. (*Nature Communications*, 2025).

Our primary quality standard was a sequencing depth of >1 million aligned reads (Figure below, red dashed line). However, some samples fall below 1 million aligned reads. We chose to retain these samples because they represent highly valuable experimental sets—containing essential matched pairs of Control and ZSWIM8 knockout samples—that are critical for our comparative analysis.

We strictly excluded only those datasets with extremely low coverage. Specifically, two samples with fewer than 0.01 million aligned reads (highlighted by the red box) were removed. To minimize sampling bias, the final miRNA-seq dataset encompasses 25 distinct cell lines and tissues, with an average of 5.92 samples per cell line/tissue (See Methods lines 943-948).

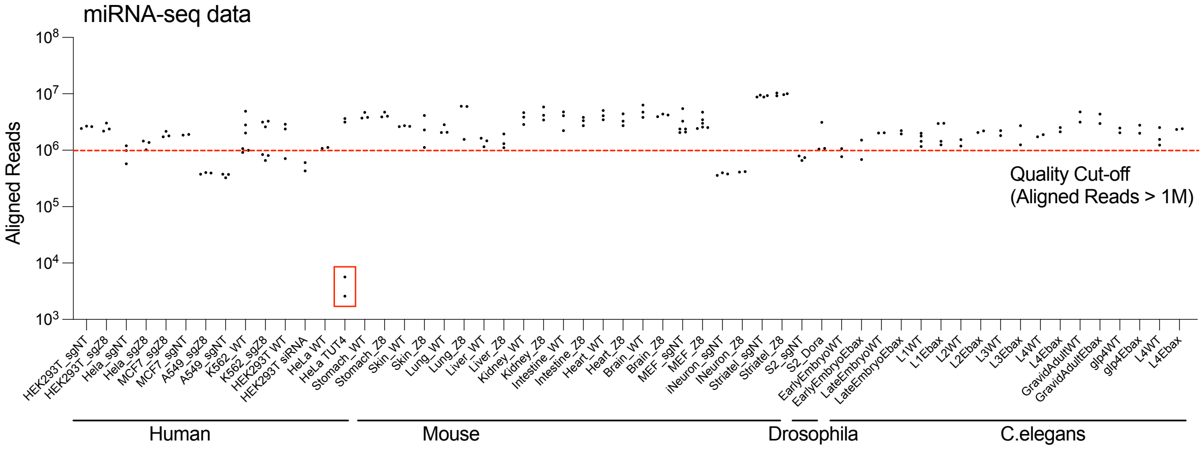

D. Classification of hybrid confidence

In addition to the dataset-level quality controls described above, we also implemented interaction-level filters to address biases related to seed preferences and binding stability. As stated in response to "Other suggestions" #4, H=high-confidence interactions must possess either canonical seed pairing (8mer, 7mer, 6mer, or offset 6mer) or significant Piranha peak support ($p < 0.01$) coupled with structural features defined by Hall et al. (2025), including continuous seed match ≥4 nt, extensive 3' pairing ≥10 nt, and geometric offset between -4 and +6 nucleotides.

As shown in the comparative assessment (Figure S4B), our filtering effectively separates signal from noise. The high-confidence dataset is dominated by canonical seed matches (8mer, 7mer-m8, 7mer-A1), whereas the excluded background is composed almost entirely of non-canonical interactions. High-confidence interactions also exhibit more favorable thermodynamic stability (median $\Delta G \sim -15.7$ kcal/mol) compared to background (median ~ -15.0 kcal/mol), with the seed type composition representing the primary distinguishing feature between the two groups.

By combining strict dataset exclusion criteria with these rigorous interaction-level filters, CLASHub database presents biologically relevant miRNA-target interaction data. These updates are described in the revised Result (lines 245-251), Methods (lines 863-882, 991-995).

6. Finally, the authors might consider discussing future applications of large language models (LLMs) to interact with aggregated datasets, which could open new opportunities for exploratory analyses.

We agree that Large Language Models (LLMs) hold great promise for enhancing data accessibility and enabling intuitive exploration of complex, multi-layered datasets. In the revised Discussion (lines 478-485), we have added a paragraph outlining our future roadmap to incorporate LLM-based approaches. This will facilitate natural language–based interaction with CLASHub, helping users generate hypotheses and discover patterns within the aggregated data more efficiently.

Reviewer #4 (Remarks to the Author):

The authors present CLASHub, an online platform that integrates CLASH, miRNA-seq, and RNA-seq data to study miRNA activity. Different cell and tissue types from multiple model organisms are represented, using both already published and newly generated data, including ZSWIM8-knockout data to facilitate study of target RNA-directed miRNA degradation. CLASHub aims to characterize miRNA activity, including distinguishing between isoforms. The method uses integration of validated miRNA-mRNA association data, miRNA expression levels, and expression levels of targets. CLASHub is useful both for scientists who do not have a background in bioinformatics as well as to streamline studies of miRNA activity.

CLASHub has potential to enable robust assessment of miRNA activity, but the manuscript currently lacks clarity regarding how the platform was constructed, expected false positives and negatives, and reproducibility. Thus, as currently presented, the efficacy of the method is difficult to assess. The utility of CLASHub could also increase with further customizability. Below, are areas that require attention.

We thank the reviewer for the suggestions that are fundamental to increasing users' confidence on CLASHub.

1. An explanation of how the data was integrated and standardized would increase confidence for potential users, especially those that are familiar with bioinformatics and doing similar processes to those performed by CLASHub on their own. Similarly, how were the CLASH, RNA-seq, and miRNA-seq datasets selected to ensure quality and avoid bias?

To handle variability and discrepancies in the input datasets, CLASHub employs a standardized "Analyzer" pipeline to process all CLASH, RNA-seq, and miRNA-seq analyses. We have expanded the Methods section to detail the construction of analysis pipelines for CLASH (lines 843-897), miRNA-seq (lines 923-953), RNA-seq (lines 954-987), and cumulative fraction curve (lines 1007-1023).

We also provided detailed schematic diagrams in Figures 5 and S4 for CLASH, S5 and S6 for miRNA-seq, S7 and S8 for RNA-seq, and Figure 6 for the cumulative fraction curve to illustrate the step-by-step logic of each pipeline. As mentioned in the Methods section, the full source code is publicly accessible at https://github.com/UF-Xie-Lab/CLASHub.

To ensure high data quality and reduce potential bias, CLASHub only includes datasets with more than 5,000 unique miRNA-target hybrids and ensured that each cell line or tissue contains at least two biological replicates (lines 883-897). Furthermore, only 'high-confidence' miRNA-target

hybrids that satisfy stringent filtering criteria are displayed for the database, minimizing false positives. Specifically, the CLASHub output table now includes two new columns: "Piranha peak p-value" (Uren et al., 2012) and "Confidence" (Table S5). We have implemented a rigorous classification strategy to stratify interactions: “High Confidence interactions” are mapped to Ensembl-annotated mRNAs, lncRNAs or lincRNAs that satisfy one of the following criteria: 1. Canonical Seed Match**:** 8mer, 7mer, 6mer, or offset-6mer. or 2. Non-canonical**:** Interactions lacking canonical seeds must possess both significant peak (Piranha $p < 0.01$) and meet the strict structural geometry defined by Hall et al. (2025) [continuous seed match ≥ 4 nt, extensive 3′ pairing ≥ 10 nt, and offset (nt difference in unpair region in target and miRNA) -4 to +6 nt]. “Low Confidence Interactions” are mapped to potential target genes (mRNA/lncRNA/lincRNA) that lack canonical seeds and fail to meet the statistical or structural requirements for high confidence. “Background Interactions” are mapped to noise-prone RNA classes (e.g., rRNA, tRNA, pseudogenes) that are not mRNAs, lncRNAs or lincRNAs. An example of target sequence distribution after “Analyzer” processing is given in Figure S4B. The Results section (lines 245–255) and the Methods section (lines 863-882) have been updated.

If the users desire an unbiased view of all miRNA target interactions, a full list of un-filtered miRNA-target hybrids is available by processing the raw data through the “Analyzer” module.

More details about dataset selection are provided in responses to comment #3 below.

2. Is it necessary for the method to rely on AQ-seq data? How does the exclusion of standard smRNA-seq impact the pipeline? While limiting to AQ-seq data could be argued to increase accuracy, the plethora of smRNA-seq datasets that are not AQ-seq may add insights missed by this limitation.

We agree with the reviewer that relying solely on AQ-seq data could limit the applicability of CLASHub. For the “Database”, we only included AQ-seq data to ensure accurate representation of miRNA abundance. However, we have expanded the functionality of the “Analyzer” module so that users can analyze data from any small RNA-seq library preparation protocol (Figures S5 and S6).

Specifically, the “Analyzer” module now supports any 5′ and 3′ UMI length (in base pairs). For AQ-seq data, users enter the appropriate UMI lengths (e.g., 4 bp at each end), and CLASHub performs PCR duplicate collapsing followed by UMI removal prior to miRNA mapping. For standard small RNA-seq libraries lacking UMIs, such as those prepared with the Illumina TruSeq Small RNA Kit or the NEBNext Small RNA Library Prep Kit, users can set both UMI lengths to 0, and CLASHub automatically skips the collapsing and UMI-trimming steps, proceeding directly to mapping after adapter trimming. This flexible design also accommodates libraries with non-standard or asymmetric UMI configurations. The same UMI length configuration has been added to the CLASH analysis and RNA-seq analysis module as well, enabling users to process CLASH and RNA-seq libraries with or without UMIs.

We have updated Figures 5A, S5, S6, S7, and S8, the Results section (Lines 237-239 and lines 263-268), and the Methods section (CLASH Analysis, lines 847-852; miRNA-Seq Data Analysis, lines 926-938) to describe these new options.

3. How would CLASHub handle variability and discrepancies in datasets used for input? Were datasets excluded to avoid this?

In the original CLASHub construction, we included all publicly available CLASH datasets without screening. We reasoned that a comprehensive list of hybrid reads would allow users to unbiasedly screen for *bona fide* miRNA-target interactions. Considering this comment and Reviewer 2's "Other suggestions" comment #5, we recognize that datasets of variable quality may introduce noise and reduce user confidence. We have therefore implemented a dataset quality control framework, as described below.

A. Quality screen of CLASH datasets

To systematically evaluate CLASH dataset quality, we compared the number of unique miRNA–target hybrids across both our newly generated CLASH data and publicly available datasets. We found that 98% of our newly generated samples yielded more than 5,000 unique hybrids, suggesting that this benchmark is reliably achievable under optimized experimental conditions. In contrast, 33.7% of publicly available datasets fell below this threshold, likely reflecting variable library preparation quality in previous studies (figure below).

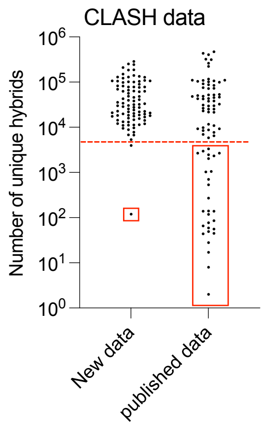

To validate that >5000 unique hybrid is sufficiently stringent to capture biologically meaningful interactions, we examined two well-established ubiquitous miRNA–target pairs: miR-7:OIP5-AS1 (Cyrano) and miR-92a:OIP5-AS1, both of which have been experimentally validated in prior studies (Kleaveland et al., 2018; Li et al., 2020 PMID:32497496; Shi et al., 2023). Reassuringly, both interactions were detectable across all 15 human cell lines and tissues retained in CLASHub after applying the quality cutoff of >5000 unique hybrids (screenshot below). While we

acknowledge that not every *bona fide* interaction will be captured in every dataset, these results demonstrate that the current quality cutoff strikes a balance between data inclusivity and reliability, and is sufficient for users to identify interactions of interest.

CLASH: OIP5-AS1 in All Human Sources

Filter by Region: All Regions   Filter by Site Type: All Site Types   Download Table

miRNA Name	Pairing Pattern (UNAfold)	Gene Name	Gene ID	Conservation Score	Free Energy (kcal/mol)	Gene Type	Target Site Region	Genome Position	Target Site Type	Total Cell Lines Out of 15 Tissues/Lines	Specific Cell Lines (Click to view details)
miR-7-5p	miRNA: 5' UGGAAGACUA--GUGAUUUUGUUGUU ||||||| |||||||||||||| target: 3' ACCUUCUGUAACCACUAAAACAACAA	OIP5-AS1	ENSG00000247556	6.53	-27.6	lncRNA	NULL	chr15:41300574-41300599	8mer	15	501Mel, A549, Colorectal tissue, D425, ES2, H1299, HCT116, HEK293T, HepG2, MB002, MDA-MB-231, Ovcar8, T98G, TIVE, U87MG
miR-92a-3p	miRNA: 5' UAUUGCACUUGUCCCGGCCUGU ||||||| |.|||||| target: 3' AUAACGUGUAUAGGGCCAAAAU	OIP5-AS1	ENSG00000247556	5.01	-23.6	lncRNA	NULL	chr15:41300490-41300511	8mer	15	501Mel, A549, Colorectal tissue, D425, ES2, H1299, HCT116, HEK293T, HepG2, MB002, MDA-MB-231, Ovcar8, T98G, TIVE, U87MG

Based on the quality cutoff of >5,000 unique miRNA–target hybrids per CLASH sample, datasets falling below this threshold—indicated by the red dashed line and red boxes—were excluded from CLASHub (Figure below), as low hybrid counts reduce the likelihood that *bona fide* miRNA–target interactions are adequately represented. Excluded datasets include Huh7.5 samples (Moore et al., 2015), liver tissue sample, and *C. elegans* L3 stage libraries (Grosswendt et al., 2014). One exception was made for a single HepG2 sample that fell marginally below the cutoff; this dataset was retained to preserve a complete set of three biological replicates for this cell line.

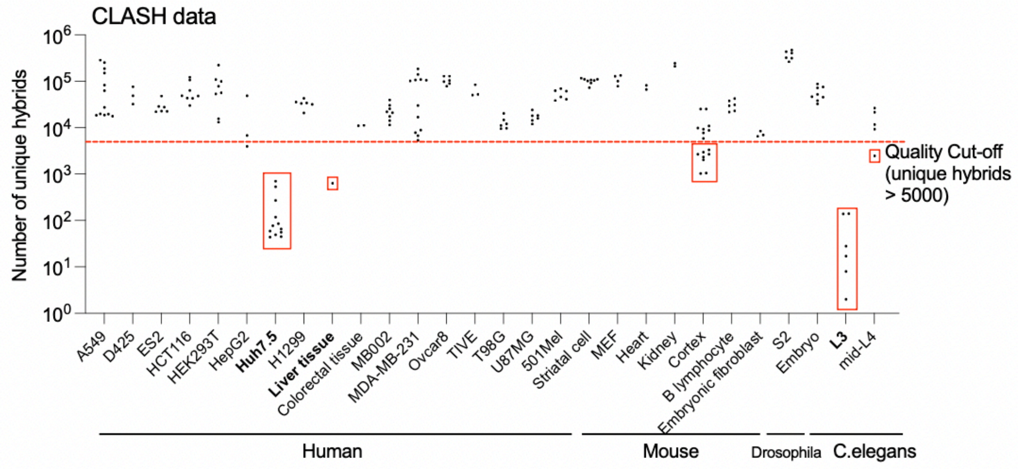

To further minimize potential bias, we required that each cell line or tissue represented in CLASHub be supported by at least two independent biological samples, with a mean of 5.84 samples per tissue/cell line across the 25 included cell lines and tissues (Table S1).

We have expanded the Methods section to detail the construction of the CLASH database (lines 883-897).

B. Quality screen of RNA-seq datasets

To retain high-quality for RNA-seq data, we implemented filtering criteria that require >10 million aligned reads and a genome alignment rate >80% (See Methods lines 983-987).

In our initial dataset, several mouse RNA-seq samples originated from previous studies (e.g., Li et al., 2017) did not meet our sequencing depth requirements, with most falling between 1 million and 10 million aligned reads (Figure below, indicated by the red box in the "Aligned Reads" figure). Consequently, we excluded these and replaced them with higher-depth, recently published mouse RNA-seq data (e.g., Shi CY et al., 2023) that satisfied both the read count and alignment rate thresholds (Figures below, indicated by the green box). Beyond the mouse samples, additional datasets were removed based on specific quality failures: two MB002 samples were excluded for insufficient read depth; two T98G, two *Drosophila*, and three *C. elegans* samples were removed due to poor alignment rates; and two *C. elegans* samples were excluded for lacking sufficient biological replication. In total, 73 new mouse samples were added to replace the excluded data (Table S2).

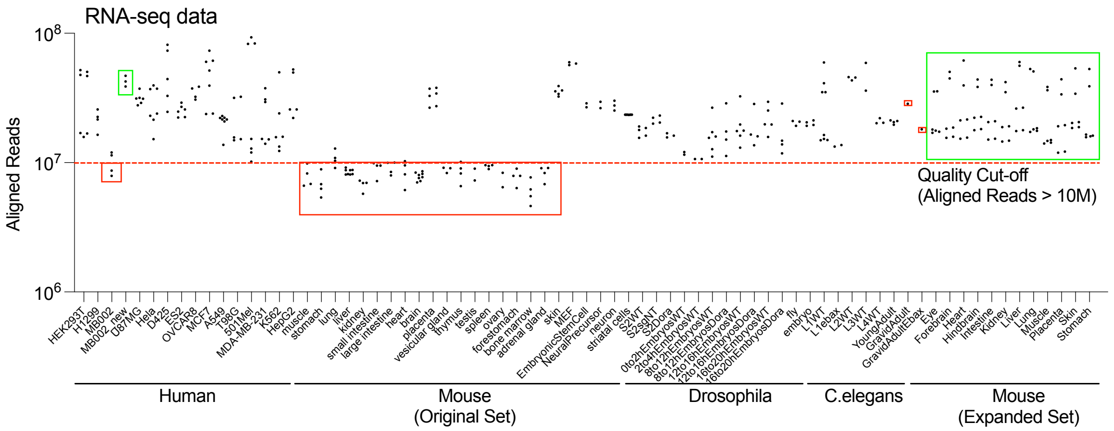

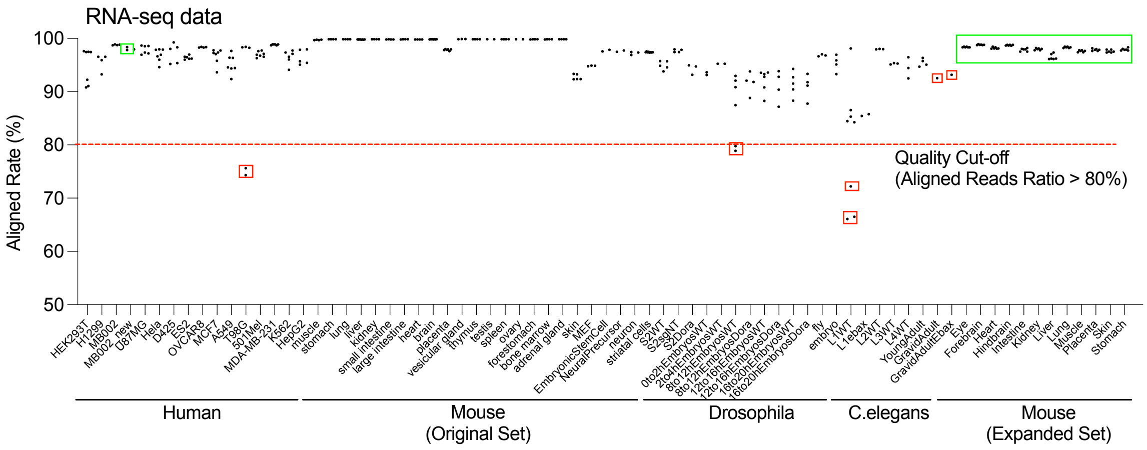

C. Quality screen of miRNA-seq datasets

To ensure high-quality quantification of miRNA abundance, we prioritized data generated via AQ-seq (Accurate Quantification by Sequencing). We selected datasets from Kim et al. (*Nucleic Acids Research*, 2020), Shi et al. and Han et al. (*Science*, 2020), Jones et al. (*Genes & Development*, 2023), Stubna et al. (*RNA*, 2024), and Li et al. (*Nature Communications*, 2025).

Our primary quality standard was a sequencing depth of >1 million aligned reads (Figure below, red dashed line, figure below). However, some samples fall below 1 million aligned reads. We chose to retain these samples because they represent highly valuable experimental sets—containing essential matched pairs of Control and ZSWIM8 knockout samples—that are critical for our comparative analysis.

We strictly excluded only those datasets with extremely low coverage. Specifically, two samples with fewer than 0.01 million aligned reads (highlighted by the red box) were removed. To minimize sampling bias, the final miRNA-seq dataset encompasses 25 distinct cell lines and tissues, with an average of 5.92 samples per cell line/tissue (See Methods lines 943-948).

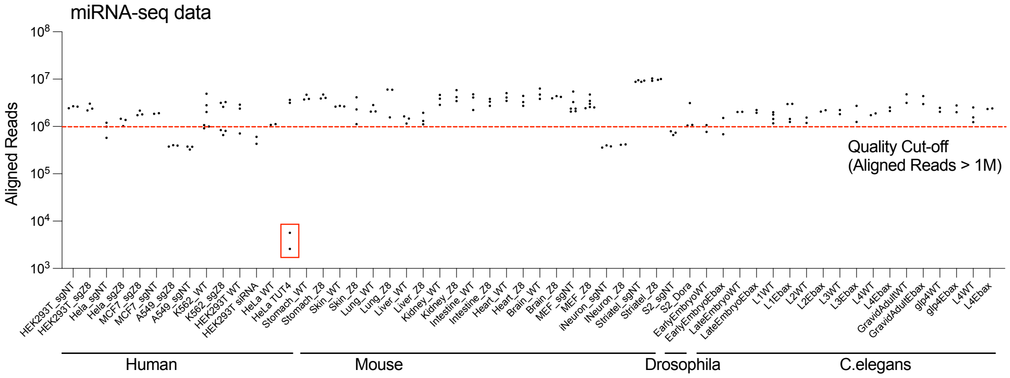

4. Some researchers may want further specificity in the model – could CLASHub enable different metrics from TargetScan, such as context score thresholds, rather than simply conserved versus nonconserved targets?

We agree that incorporating Context++ score thresholds from TargetScan would significantly enhance the specificity of the cumulative fraction curve analysis. We have integrated Context++ scores from TargetScan (Agarwal et al., *eLife*, 2015) into the updated version of CLASHub for available species (*Drosophila* Release 7.2, Human Release 8, and Mouse Release 8). We also have revised the Methods section (lines 1004-1006) and updated Figure 6A to reflect this new functionality (lines 306-309). While we retained the original conservation-based approach as the "Standard Analysis," users can now select the "Stringent Filtering" option. When selected,

CLASHub automatically identifies the top 25% of transcripts with the most favorable Context++ scores, allowing users to specifically focus on potential high-efficacy targets.

As shown in the new Figure 6C (lines 315-317), we validated this stringent analysis using dme-miR-999-3p. The result demonstrates the difference between the broad target definition (Standard Analysis) and the high-confidence targets extracted via Context++ scores, which typically exhibit more pronounced repression.

5. Indicated "limited reproducibility across samples" in line 322 – but this could just be differences in cell lines.

Agreed, we revised the original sentence (lines 350-353) in the manuscript to:

"Third, *ATP6V1G1* hybrids were broadly detected across 9 cell lines and 1 tissue, whereas other candidate hybrids were observed in only one to four cell lines/tissues, suggesting that *ATP6V1G1* represents a more robust and broadly relevant TDMD trigger for miR-335-3p."

6. In Figure 7B and S8A, one nucleotide is not complementary – how is this indicated as 10 consecutive nucleotides in the text?

To improve clarity, we have revised the original sentence (lines 346-348) "First, its base-pairing pattern includes a contiguous 7-nucleotide seed match and 10 consecutive nucleotides of complementarity at the miRNA 3′ end (including G:U wobble pairings), a configuration characteristic of TDMD-inducing sites (Figure 7B and S9A)."

7. In figures 7D and E, showing significance testing would be useful for more than just one miRNA, especially since the lack of upregulation in passenger strands is less clear for the miRNAs where significance testing not shown for.

To clarify the statistical significance of miRNA expression changes beyond just one example, we have revised Figures 7D and 7E to include adjusted p-values for several representative guide and passenger strands, including miR-335-3p and miR-335-5p. In these scatter plots, upregulated guide strands with statistical significance (adjusted $p < 0.02$) are highlighted in red, while corresponding passenger strands that are not significantly changed are shown in blue.

As stated in the original manuscript, the complete differential expression results for all detected miRNAs—including baseMean, log2 fold change, and adjusted p-values—are available in Supplementary Tables S13 and S14, which allow readers to evaluate the significance of changes across all miRNAs.

8. Adding the option to process the RNA-seq input with exon-intron split analysis (Gaidatzis et al Nature Biotechnology, 2015) could substantially decrease false positives.

We thank the reviewer for the suggestion of incorporating Exon-Intron Split Analysis (EISA), which would significantly enhance the ability to distinguish transcriptional from post-transcriptional regulation and effectively reduces false positives.

Accordingly, we have upgraded the RNA-seq analysis module in CLASHub to support a dual-pipeline approach (Figure S7A, step 10; Figure S8, step 23; Results, lines 291–296; Methods, lines 966–982). By default, CLASHub performs standard gene-level quantification (Counts and TPM) using full Ensembl annotations to ensure comprehensive expression profiling. In the updated version, we added an optional EISA module based on Gaidatzis et al. 2015. When enabled, CLASHub generates custom exon- and intron-specific GTF files (excluding overlapping genes to ensure unambiguous read assignment). It then quantifies exonic and intronic changes separately using DESeq2. In this mode, genes are retained only if they show sufficient expression (mean counts ≥ 24) in both exonic and intronic regions. Each gene is then classified into one of three regulation types — Post-transcriptional, Transcriptional, or Ambiguous — in the EISA output table. A gene is classified as "Post-transcriptional" only if there is a positive divergence between exonic and intronic changes ($|\Delta Exon - \Delta Intron| > 1$).
